# Integrated Full-Length Transcriptomics, RNA-Seq and Peptidomics Define an Isoform-Resolved Skin Defense Repertoire in the Chinese Spiny Frog (*Quasipaa spinosa*)

**DOI:** 10.3390/biology15201802

**Published:** 2026-10-09

**Authors:** Jie Chen, Yikai Feng, Xinyi Qian, Zhihua Lin

**Affiliations:** 1 Industrial College of Traditional Chinese Medicine and Health, Lishui University, Lishui 323000, China; linzhihua1015@126.com; 2 College of Agriculture and Biotechnology, Lishui University, Lishui 323000, China; lsufyikai@126.com (Y.F.); lsxyqianxinyi@126.com (X.Q.)

**Keywords:** *Quasipaa spinosa*, amphibian skin immunity, full-length transcriptomics, isoform diversity, antimicrobial peptides, host defense peptides, peptidomics, multi-omics integration

## Abstract

Amphibian skin relies on secreted effectors and inducible immune pathways, but short-read RNA sequencing cannot reliably resolve the transcript isoforms and precursor structures underlying this defense system. We generated the first full-length skin transcriptome for the Chinese spiny frog (*Quasipaa spinosa*) by integrating pooled PacBio Iso-Seq data with six Illumina RNA-seq libraries and six skin peptidomic libraries from control and *Proteus mirabilis*-challenged frogs. Because Iso-Seq RNA was pooled across conditions, the long-read data were used to define transcript structure rather than condition-specific isoform changes. The reference contained 58,388 transcripts from 34,247 genes, including 32,215 non-reference models, and 5907 of 19,143 reconstructed loci had multiple isoforms. Among 108,147 predicted ORFs, 17,169 carried an N-terminal secretion signal, and 72 secretory and immune-effector ORFs from 46 genes were prioritized. Gene-level RNA-seq identified 72 differentially expressed genes at Benjamini–Hochberg-adjusted *p* < 0.05 and |log_2_FC| ≥ 1; 27 had higher expression in challenged skin and 45 in control skin. No GO term remained significant after correction, whereas three KEGG pathways did. Among these 72 genes, 60 (83.3%) had at least two full-length models, 53 (73.6%) contained at least one complete ORF, and 4 (5.6%) had peptide-sequence support. Reported peptide sequences matched 1029 entries across the full-length catalog, but shared peptide matches were not treated as ORF-specific evidence. Together, these data establish an isoform-resolved skin-defense resource for *Q. spinosa* and a framework for prioritizing secretory and immune candidates for isoform-specific and functional validation.

## Simple Summary

Amphibian skin contains many molecules that help protect the animal from microbes, but standard short-read sequencing cannot show the complete RNA transcripts that encode these products. We combined full-length RNA sequencing, conventional RNA sequencing, and mass spectrometry of skin peptides to build a detailed skin-defense reference for the Chinese spiny frog (*Quasipaa spinosa*). To our knowledge, this is the first full-length skin transcriptome reported for this species. The long-read data revealed complete transcript structures, alternative forms, and a broad set of candidate secreted defense molecules. Short-read data placed this resource in the context of a 24 h *Proteus mirabilis* challenge, where the gene-level response was modest after strict statistical correction rather than broadly activated. Peptide detections provided additional sequence-level support for a subset of predicted products. Together, the study establishes a high-resolution resource for studying frog skin defense and prioritizes candidates for future testing of specific transcript forms, peptide processing, and biological function, including work relevant to disease resistance in aquaculture.

## 1. Introduction

Amphibian skin is both a physical barrier and an active immune surface. Its mucus layer and antimicrobial peptides help limit microbial attachment and invasion [1,2]. Skin-derived peptides can also shape inflammation, leukocyte recruitment, barrier repair, and local microbial communities [3,4,5,6]. Classic studies established frog skin peptides as major components of amphibian chemical defense [7,8,9,10,11]. These constitutive and inducible defenses make skin a useful tissue in which to examine responses to infection.

The Chinese spiny frog, *Quasipaa spinosa*, is an economically important aquaculture species in which bacterial skin disease is a practical concern. Several peptides from this species already have documented antimicrobial or immunomodulatory activity. A β-defensin and the cathelicidin QS-CATH showed antimicrobial and macrophage-related effects [12,13], while QsLEAP2, hepcidin, and QsCATH were also linked to bacterial control and macrophage protection [14,15,16]. These studies, however, focused on individual molecules. They did not resolve the broader transcript architecture from which the skin defense repertoire is produced.

A previous short-read RNA-seq study of *Q. spinosa* challenged with *Proteus mirabilis* identified challenge-associated genes and immune pathways [17]. Short reads are well suited to gene-level quantification, but they are less reliable for defining complete transcript boundaries, exon combinations, and coding sequences. This is especially important for secreted products because alternative splicing can alter precursor coding structure and, in amphibian antimicrobial-peptide genes, has been shown to generate both prepro- and mature-peptide sequence diversity [18]. Peptide-first searches offer complementary evidence, but they also depend on the reference database and on whether relevant peptide families are already represented. Divergent or previously unannotated amphibian products can therefore be missed [18,19].

Long-read transcriptomics helps resolve these structural questions by preserving transcript connectivity within individual molecules. It can recover exon organization, alternative isoforms, untranslated regions, and complete or near-complete open reading frames that are difficult to distinguish with short reads alone [20,21,22,23,24,25]. Full-length and hybrid sequencing have already expanded amphibian skin transcript resources, including those of the Chinese giant salamander [26] and the secretory-peptide repertoire of *Odorrana grahami* [27]. Short-read studies in other frogs have also shown the value of tissue-specific immune and stress profiling [28]. A species-specific full-length reference is therefore useful not only for genome annotation but also for interpreting precursor structures.

These approaches answer different questions. A full-length transcript catalog defines which isoforms and precursor arrangements are present, short-read RNA-seq measures gene-level abundance changes after challenge, and mass spectrometry asks whether peptide sequences from the translated catalog can be detected in skin. Recent amphibian studies show the value of combining these layers, while also highlighting the effects of sample preparation, reference completeness, and detection sensitivity on peptide discovery [27,29]. We therefore analyzed transcript structure, challenge-associated expression, and peptide support as distinct forms of evidence.

*Q. spinosa* still lacks a species-specific full-length skin transcriptome. Without such a reference, it is difficult to interpret known defense genes at the isoform level or to distinguish conventional antimicrobial-peptide precursors from other secreted immune effectors. It also remains unclear how recently characterized *Q. spinosa* peptides fit within the broader challenge-responsive skin transcriptome [14,15,16,17].

We expected *P. mirabilis* challenge to alter a subset of genes involved in innate immune signaling and secreted skin defense. We also expected full-length sequencing to uncover multiple isoforms and complete precursor ORFs at defense-related loci. Finally, we asked whether at least some of these reconstructed ORFs would be supported by peptide sequences detected in skin.

To address these questions, we combined one pooled PacBio Iso-Seq skin library with six independent Illumina RNA-seq libraries and six independent skin peptidomic libraries from control and *P. mirabilis*-challenged frogs. We built and annotated a full-length skin transcript reference, measured the 24 h gene-level response, prioritized secretory and immune-effector candidates, examined representative isoform structures, and mapped detected peptide sequences back to translated full-length ORFs. Defense-peptide candidates received additional precursor-based screening. Throughout the analysis, sequence prediction, expression evidence, and peptide support were kept separate so that each candidate could be interpreted according to the evidence actually available.

## 2. Materials and Methods

### 2.1. Animals, Bacterial Culture and Challenge

Twenty adult male Chinese spiny frogs, approximately one year old and weighing 150 ± 10 g, were obtained from Longquan Fuxi Agricultural Development Co., Ltd. (Lishui, China). Frogs were randomly assigned to challenge and control groups (*n* = 10 per group) and acclimated in 100 L plastic enclosures at 18–20 °C for 2 weeks. Nine frogs per group were allocated to the three assay cohorts described below; the remaining frog was retained as a reserve, housed separately, and was not used for sampling or downstream analysis. The bacterial-challenge protocol was adapted from Liu et al. [17], who documented *P. mirabilis* infection and pathological changes after a comparable 24 h immersion exposure. The challenge suspension was adjusted to OD600 = 0.7; three independent vessels per condition each contained 1000 mL of medium and three frogs, with controls arranged identically. All animal procedures followed the Lishui University Guidelines for the Care and Use of Laboratory Animals and were approved by the institutional authority (Approval No. LSU-AREC-202512016).

### 2.2. Experimental Design and Skin Sampling

Skin was collected 24 h after exposure from three independent assay cohorts, each containing three control frogs and three *P. mirabilis*-challenged frogs. Frogs were anesthetized with 100 mg/L tricaine methanesulfonate (MS-222; Sigma-Aldrich, St. Louis, MO, USA). Dorsal skin was collected from the same anatomical region, rinsed with 0.9% saline, snap-frozen in liquid nitrogen, and stored at −80 °C. For the PacBio cohort, RNA was extracted separately from three control and three challenged frogs and pooled in equal amounts before full-length library construction. The RNA-seq and peptidomics cohorts retained three independent biological replicates per condition. The three assays used different frogs; no animal contributed to more than one assay. After skin sampling, the frogs were euthanized by prolonged immersion in an overdose of MS-222.

### 2.3. RNA Extraction and Quality Assessment

Total RNA was extracted from dorsal skin with TRIzol Reagent (Invitrogen, Thermo Fisher Scientific, Waltham, MA, USA). RNA quantity was measured with a NanoDrop 2000 spectrophotometer (Thermo Fisher Scientific, Waltham, MA, USA) and a Qubit 4 fluorometer (Thermo Fisher Scientific, Waltham, MA, USA), and RNA integrity was assessed by agarose-gel electrophoresis and an Agilent 2100 Bioanalyzer (Agilent Technologies, Santa Clara, CA, USA). Samples with A260/A280 ratios of 1.8–2.2 and RNA integrity numbers above 7.0 were used for library preparation. For PacBio sequencing, RNA from three control and three challenged frogs was pooled in equal amounts. The six RNA-seq libraries were prepared separately from three control and three challenged frogs.

### 2.4. PacBio Iso-Seq Library Preparation and Full-Length Sequencing

Full-length library preparation and sequencing were carried out by Shanghai Majorbio Bio-Pharm Technology Co., Ltd. (Shanghai, China). Pooled total RNA was used directly for full-length cDNA synthesis and amplification with the Iso-Seq Express 2.0 and Kinnex Full-Length RNA workflows, without a separate poly(A)+ RNA-enrichment step. Amplified cDNA was purified with SMRTbell Cleanup Beads (Pacific Biosciences, Menlo Park, CA, USA), converted into SMRTbell libraries, and sequenced on the PacBio Revio platform (Pacific Biosciences, Menlo Park, CA, USA). Primary signal processing was performed with SMRT Link v13.5.0.286960. HiFi circular consensus sequence (CCS) reads were generated with CCS v6.2.0, followed by adapter removal and demultiplexing with Lima v2.1.0 before full-length transcript reconstruction.

### 2.5. Full-Length Transcript Reconstruction, Structural Classification and Annotation

After CCS generation and adapter processing, full-length non-chimeric reads were identified from reads containing both 5′ and 3′ primer sequences and, when detected, a poly(A) tail. Full-length reads were clustered and polished to generate isoform consensus sequences. The reference genome was the chromosome-level *Quasipaa spinosa* assembly of Hu et al. [30] (Dryad DOI: 10.5061/dryad.ghx3ffbpw), spanning approximately 2.84 Gb with 99.30% anchored to 13 chromosomes and 26,173 predicted protein-coding genes. The consensus sequences were aligned with pbmm2 v1.8.0 and then collapsed to reduce redundancy. To further assess full-length read support, CCS-derived reads were filtered by requiring both 5′ and 3′ primer support and a ≥20-nt poly(A) tail and were then remapped to the final transcript catalog to quantify full-model read support. Transcript models were further evaluated with SQANTI3 using splice-junction evidence from the six RNA-seq libraries. Detailed QC statistics are provided in Table S1, and the rarefaction analysis is shown in Figure S1. Transcript completeness was assessed with BUSCO v5.4.7 [31], and transcript structures were compared with the reference annotation using GffCompare v0.11.2 [32]. Models were assigned class codes, including complete intron-chain matches (=), junction-sharing models (j), and intergenic models (u). Functional annotation was performed against the NCBI non-redundant protein database (NR, release 2026.02), UniProt (release 2026.02), Swiss-Prot (release 2026.02), Gene Ontology (release 2026.0211), KEGG (release 2026.02), Pfam (release 38.0), eggNOG (release 2020.06), and Rfam (release 15.1) [33,34,35,36,37,38]. Protein-sequence similarity searches used DIAMOND v2.1.9 [39], and profile hidden Markov model searches used HMMER v3.2.1 [40]. The most informative supported annotation was retained for each transcript or predicted ORF.

### 2.6. ORF Prediction and Prioritization of Secretory and Immune-Effector Candidates

ORFs were identified using a combined homology- and sequence-based workflow. Transcript sequences were first searched against NR and Swiss-Prot, and coding regions supported by homology were retained. Transcripts without a supported coding region were then evaluated with TransDecoder v5.5.0 using its default minimum ORF length of 100 amino acids. Pfam domain evidence was also used to support coding-region prediction, and ORF completeness followed the TransDecoder classification. Secretory and immune-effector candidates were prioritized from ORFs with an N-terminal signal peptide predicted by SignalP 6.0 [41] and compatible functional annotations. Candidate ORFs were classified as defense-peptide precursors, chemokines, interferons, interleukin/cytokine-related effectors, or other immune-related effectors. Ambiguous assignments were reviewed manually; immune-relevant entries without direct class-specific annotation were placed in the other immune-related category, whereas entries lacking independent immune-effector support were excluded. Defense-peptide precursors were screened further for plausible monobasic or dibasic processing sites, similarity to curated amphibian host-defense peptide families, and predicted mature-peptide properties, including length, net charge, hydrophobicity, and amphipathicity. Homology to known antimicrobial peptides was assessed against APD3 (2016 release), DBAASP v2 (2016 release), and CAMPR3 (2016 release) [42,43,44]. All candidates were treated as sequence-based predictions and not as evidence of secretion, processing, or biological activity. Direct defense-peptide annotation and the broader immune-effector candidate catalog were generated independently and were not treated as sequential parent–subset sets. Representative loci were selected from candidates with at least two reconstructed full-length isoforms and one complete ORF, followed by within-class ranking based on isoform number and PacBio/RNA-seq support (Tables S2 and S3).

### 2.7. Illumina RNA-Seq Library Construction, Sequencing and Quantification

Polyadenylated RNA was enriched from each skin RNA sample with oligo(dT)-conjugated magnetic beads, fragmented, and reverse-transcribed to generate first- and second-strand cDNA. After adapter ligation, size selection, and PCR amplification, six short-read transcriptome libraries were sequenced by Shanghai Majorbio Bio-Pharm Technology Co., Ltd. (Shanghai, China) on an Illumina NovaSeq platform (Illumina, San Diego, CA, USA) to generate paired-end 151 bp reads. Raw reads were processed with fastp v0.23.4 [45] to remove adapters and low-quality bases. Clean reads were aligned to the *Quasipaa spinosa* reference genome with HISAT2 v2.2.1 [46]. Gene- and transcript-level abundances were quantified with RSEM v1.3.3 [47]. Reference-guided transcript reconstruction was performed with StringTie v2.2.1 [48], using the same reference assembly for alignment, reconstruction, and downstream annotation.

### 2.8. Differential Expression and Functional Enrichment

Gene-level expected counts from RSEM were used as input to DESeq2 v1.42.0 [49], with three biological replicates per group. TPM values were used only for descriptive abundance summaries and visualization and were not used as input to DESeq2. Differential expression was tested for the challenged-versus-control contrast, and genes with a Benjamini–Hochberg-adjusted *p* value (padj) < 0.05 and |log_2_FC| ≥ 1 were considered differentially expressed. GO enrichment analysis was performed with GOATOOLS v1.4.4 [50] using 11,959 unique GO-annotated genes as the background set. GO terms were propagated to ancestor terms using the Gene Ontology go-basic.obo release of 23 January 2026, and enrichment was tested with Fisher’s exact test followed by Benjamini–Hochberg correction. KEGG enrichment used 16,477 KEGG-annotated background genes and the same Fisher/BH procedure. Enrichment terms with padj < 0.05 were considered significant. Benjamini–Hochberg correction was applied consistently to differential-expression and enrichment analyses. For the descriptive candidate-expression ratios, TPM values were averaged across the three biological replicates in each group and calculated as log_2_[(infection mean TPM + 0.1)/(control mean TPM + 0.1)]. A pseudocount of 0.1 TPM was added to both group means to avoid undefined values when a group mean was zero. This descriptive ratio was used only for visualization and is distinct from the DESeq2-derived log_2_ fold change.

### 2.9. Peptidomics Sample Preparation and LC-MS/MS Acquisition

Skin peptidomics was performed by Shanghai Majorbio Bio-Pharm Technology Co., Ltd. (Shanghai, China) using six independent samples: three control and three challenge samples. Methanol (LC-MS grade, ≥99.8%) and chloroform (≥99.0%) were obtained from Sinopharm Chemical Reagent Co., Ltd. (Shanghai, China). LC-MS-grade water, acetonitrile, formic acid, and trifluoroacetic acid (TFA) were obtained from Thermo Fisher Scientific (Waltham, MA, USA). Peptides were extracted with methanol/chloroform/water (3:1:4, *v*/*v*/*v*), dried, reconstituted in water, quantified with a NanoDrop One spectrophotometer (Thermo Fisher Scientific, Waltham, MA, USA), and desalted on C18 disks. No enzymatic digestion, reduction, or alkylation was performed. The C18 material was activated with acetonitrile and equilibrated with aqueous acetonitrile/TFA solutions. Acidified samples (pH < 3) were loaded, washed, and eluted with 40% acetonitrile containing 0.1% TFA. Eluates were freeze-dried and reconstituted in 0.1% formic acid.

Peptides were separated on an in-house packed C18 column (15 cm × 100 μm internal diameter, 1.7-μm particle size) using a Vanquish Neo UHPLC system (Thermo Fisher Scientific, Waltham, MA, USA) and analyzed on a timsTOF HT mass spectrometer (Bruker Daltonics, Bremen, Germany) in data-dependent acquisition parallel accumulation-serial fragmentation (DDA-PASEF) mode [51]. Mobile phase A was 2% acetonitrile containing 0.1% formic acid, and mobile phase B was 80% acetonitrile containing 0.1% formic acid. The 14.4 min gradient was 4–17% B from 0 to 1.8 min, 17–35% B from 1.8 to 7.9 min, 35–45% B from 7.9 to 10.7 min, and 45–99% B from 10.7 to 13.4 min, followed by a high-organic wash and re-equilibration. The ion-source voltage was 1600 V. MS1 and MS2 spectra were acquired over 180–1800 *m*/*z*. Six PASEF ramps were collected across a 1/K0 range of 0.75–1.30 V s cm^−2^, with a cycle time of 0.39 s.

Raw Bruker .d files were searched against a protein database generated from the predicted full-length ORFs using PEAKS Studio v12 (Bioinformatics Solutions Inc., Waterloo, ON, Canada) [52]. Database searching was performed without enzyme specificity because the samples contained endogenous peptides and were not enzymatically digested. Methionine oxidation was included as a variable modification. The precursor mass tolerance was 20 ppm, and the fragment mass tolerance was 0.05 Da. Peptide/PSM identifications were filtered at 1% FDR. Fixed cysteine carbamidomethylation was not specified because reduction and alkylation were not performed.

### 2.10. Peptidomics Data Processing and Cross-Omics Integration

A peptide feature was considered detected when its quantitative area was greater than zero; features with zero area in all six libraries were excluded. Because the feature matrix contained many missing values, differential peptide abundance was not tested. Instead, peptide evidence was summarized by detection frequency and repeatability across samples. A feature was considered repeatably detected within a condition when it occurred in at least two of three biological replicates. A feature met the repeatability criterion in only one condition when it reached the ≥2/3 threshold in that condition but not in the other; complete absence from all replicates of the other condition was not required. These descriptive detection measures were not used for candidate selection. For prioritized candidate ORFs, peptide support was counted only when a matching peptide was repeatably detected (≥2/3 biological replicates in at least one condition) and was ORF-specific.

Accession identifiers from the peptide, protein-peptide, protein, and PSM tables were matched to the translated full-length ORF catalog. An ORF was considered sequence-matched when at least one identified peptide occurred within its translated sequence. Shared peptides were not interpreted as ORF-specific translation evidence. Peptide support was therefore evaluated from peptide-to-ORF sequence correspondence together with recurrence across samples. Peptide matches assigned to incomplete ORFs were also cross-checked against complete ORFs, and the resulting peptide/PSM evidence is summarized in Table S4.

## 3. Results

### 3.1. PacBio Iso-Seq Revealed Extensive Skin Transcript Diversity

**Figure 1:**
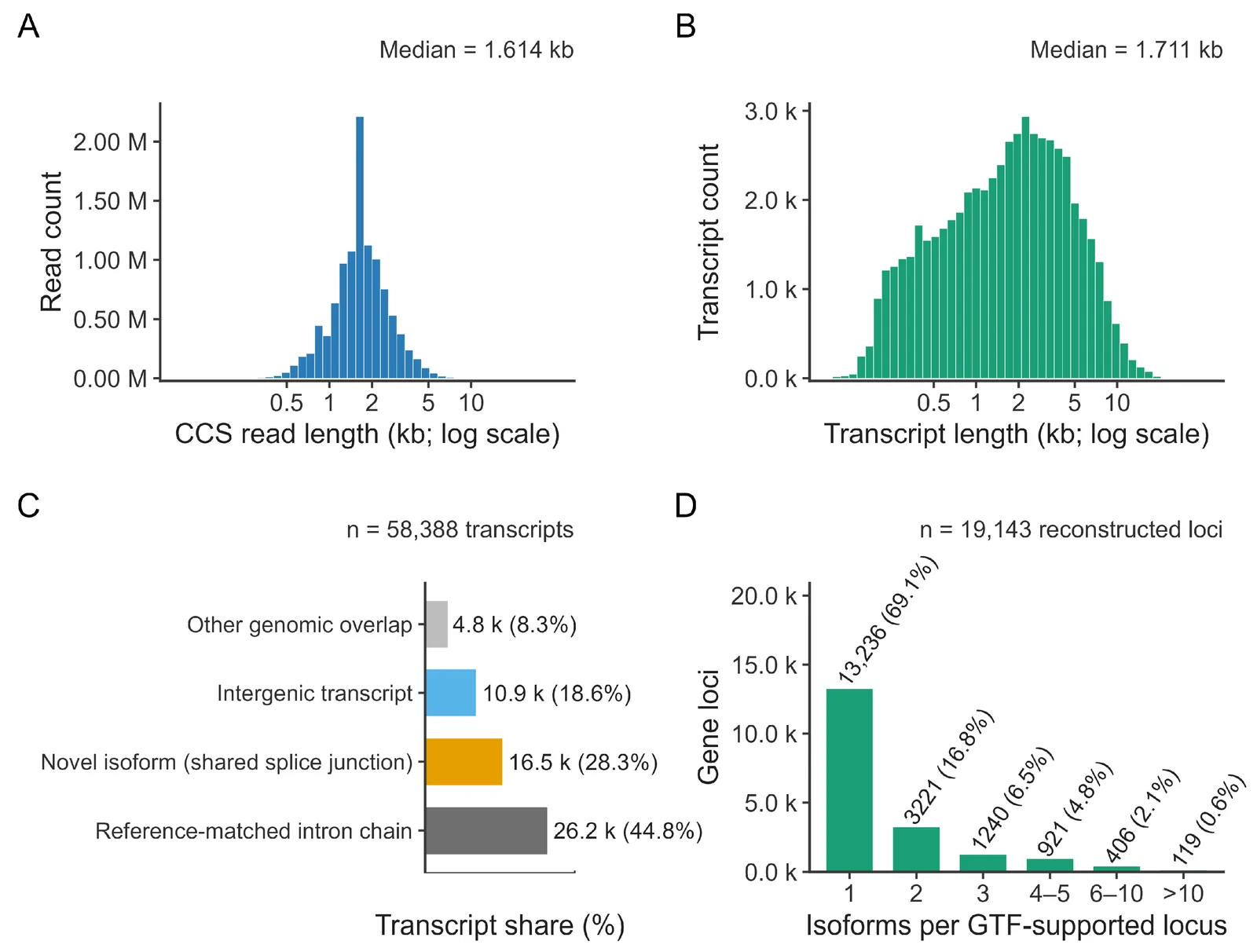
Full-length transcriptome reconstruction quality in *Quasipaa spinosa*. (**A**) Length distribution of PacBio Iso-Seq CCS reads (median, 1.614 kb). (**B**) Length distribution of reconstructed transcripts (median, 1.711 kb). (**C**) Structural classes of the final transcript catalog relative to the reference genome annotation; counts and percentages are based on all 58,388 transcripts. (**D**) Distribution of isoforms per GTF-supported genomic locus (*n* = 19,143). Here, a GTF-supported locus refers to a reconstructed genomic locus represented by at least one of the 32,215 non-reference transcript models in the final GTF.

PacBio Iso-Seq generated 10,660,747 circular consensus sequence (CCS) reads from pooled *Q. spinosa* skin (median length, 1614 bp; N50, 1926 bp; Figure 1A). Of these, 10,622,861 were classified as full-length non-chimeric (FLNC) reads, yielding 599,697 polished consensus sequences. Full-length reconstruction yielded 58,388 transcripts assigned to 34,247 genes (median length, 1711 bp; N50, 4221 bp; Figure 1B). Relative to the reference annotation, 26,173 transcripts (44.8%) were complete intron-chain matches, 16,521 (28.3%) were candidate novel isoforms sharing at least one splice junction, 10,853 (18.6%) were intergenic models, and 4841 (8.3%) had other overlap configurations (Figure 1C). Of 19,143 reconstructed loci, 5907 (30.9%) contained more than one full-length isoform (Figure 1D). Here, the 34,247 gene identifiers refer to the full catalog, whereas the 19,143 reconstructed loci refer to the 32,215 non-reference models. Additional Iso-Seq QC and rarefaction results are provided in Table S1 and Figure S1.

### 3.2. Non-Reference Transcripts Increased Isoform Diversity and Showed RNA-Seq Support

**Figure 2:**
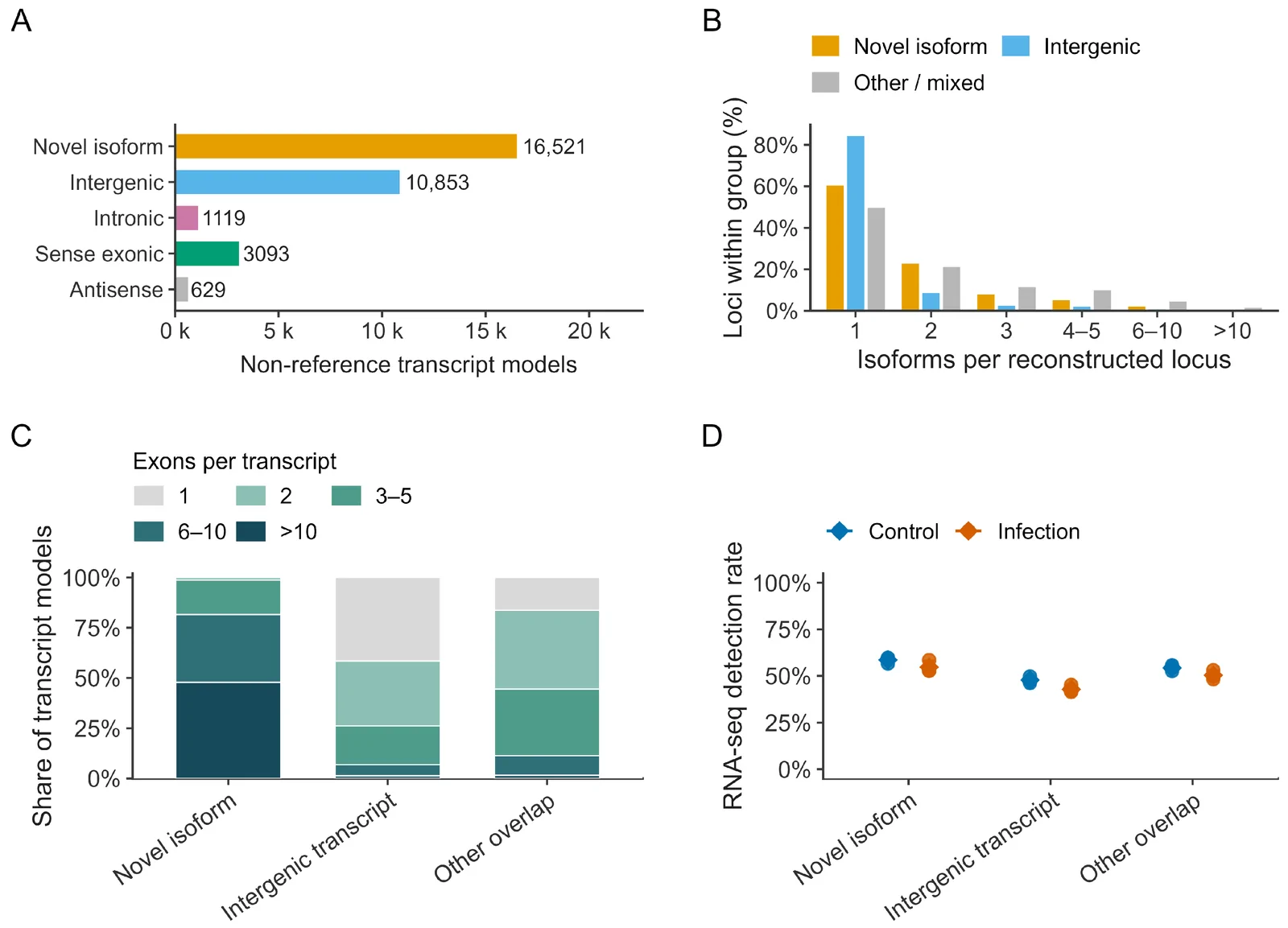
Isoform complexity and non-reference transcript architecture in *Quasipaa spinosa* skin. (**A**) Detailed class-code distribution of non-reference transcript models. (**B**) Distribution of reconstructed loci across discrete isoform-count categories (1, 2, 3, 4–5, 6–10 and >10 isoforms per locus) within each structural group; bars show the percentage of loci in each category. (**C**) Exon-count composition by structural group. (**D**) Short-read RNA-seq detection rate (TPM ≥ 1) by condition; circles show individual samples, diamonds show means and bars show SD (*n* = 3 per condition).

Non-reference models increased transcript diversity (Figure 2A–C). Multiple isoforms were common, and 81.5% of candidate novel isoforms contained at least six exons. Among the 10,853 class-u intergenic models, 4514 were single-exon, 3493 had two exons, and 2846 had three or more exons. Of the single-exon models, 1997 had TPM ≥ 1 in at least three of the six RNA-seq libraries, whereas 2517 had weaker short-read support and were retained as provisional models. The non-reference catalog comprised 32,215 models: 16,521 junction-sharing candidate novel isoforms, 10,853 intergenic models, and 4841 models with other overlap configurations. RNA-seq abundance estimates were available for 31,559 models; 13,476 junction-sharing and 9000 intergenic models reached TPM ≥ 1 in at least one sample, and the remaining 9083 consisted of other-overlap models and junction-sharing/intergenic models below TPM 1 (Figure 2D).

### 3.3. ORF Analysis Identified 72 Secretory and Immune-Effector Candidates

**Figure 3:**
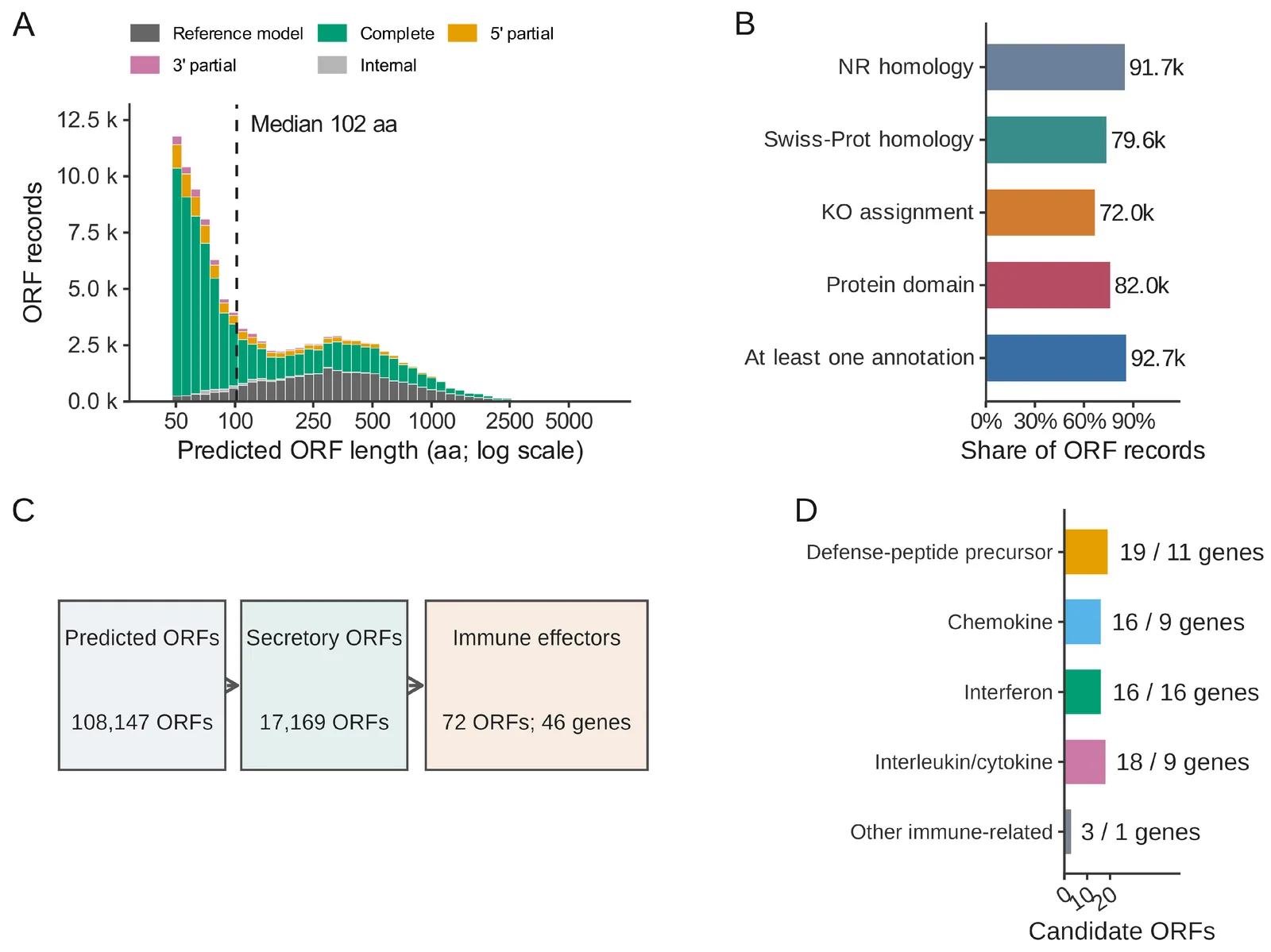
Coding potential, annotation coverage and secretory and immune-effector candidate landscape of the full-length *Quasipaa spinosa* skin transcript reference. (**A**) Predicted ORF-length distribution and ORF status. (**B**) Annotation-source coverage across ORFs; bars are not additive. (**C**) N-terminal secretory-proxy screening and manually reviewed immune-effector prioritization. (**D**) Class composition of 72 candidate ORFs from 46 genes.

The ORF catalog comprised 108,147 records from 55,258 transcript IDs. The remaining 3130 reconstructed transcripts had no retained ORF record. Median ORF length was 102 amino acids across all records, 298 amino acids when only the longest ORF per transcript was retained, and 314 amino acids for reference-matched transcripts. Of 53,350 ORFs shorter than 100 amino acids, 42,120 (79.0%) had NR, Swiss-Prot, or Pfam support and 50,883 (95.4%) were linked to transcripts detected by RNA-seq. Complete ORFs accounted for 68,517 records (63.4%), and 92,729 (85.7%) had at least one database annotation (Figure 3A,B). ORF records were linked to 26,173 reference-matched transcript models.

SignalP screening identified 17,169 ORFs with N-terminal secretion signals. Functional annotation and manual review prioritized 72 secretory and immune-effector ORFs from 46 genes: 19 defense-peptide precursors, 16 chemokines, 16 interferons, 18 interleukin/cytokine-related effectors, and 3 other immune-related ORFs (Figure 3C,D). A separate direct defense-peptide annotation screen identified 21 ORF records from 18 transcripts.

### 3.4. RNA-Seq Quality Was Consistent, with Appreciable Inter-Individual Variation

**Figure 4:**
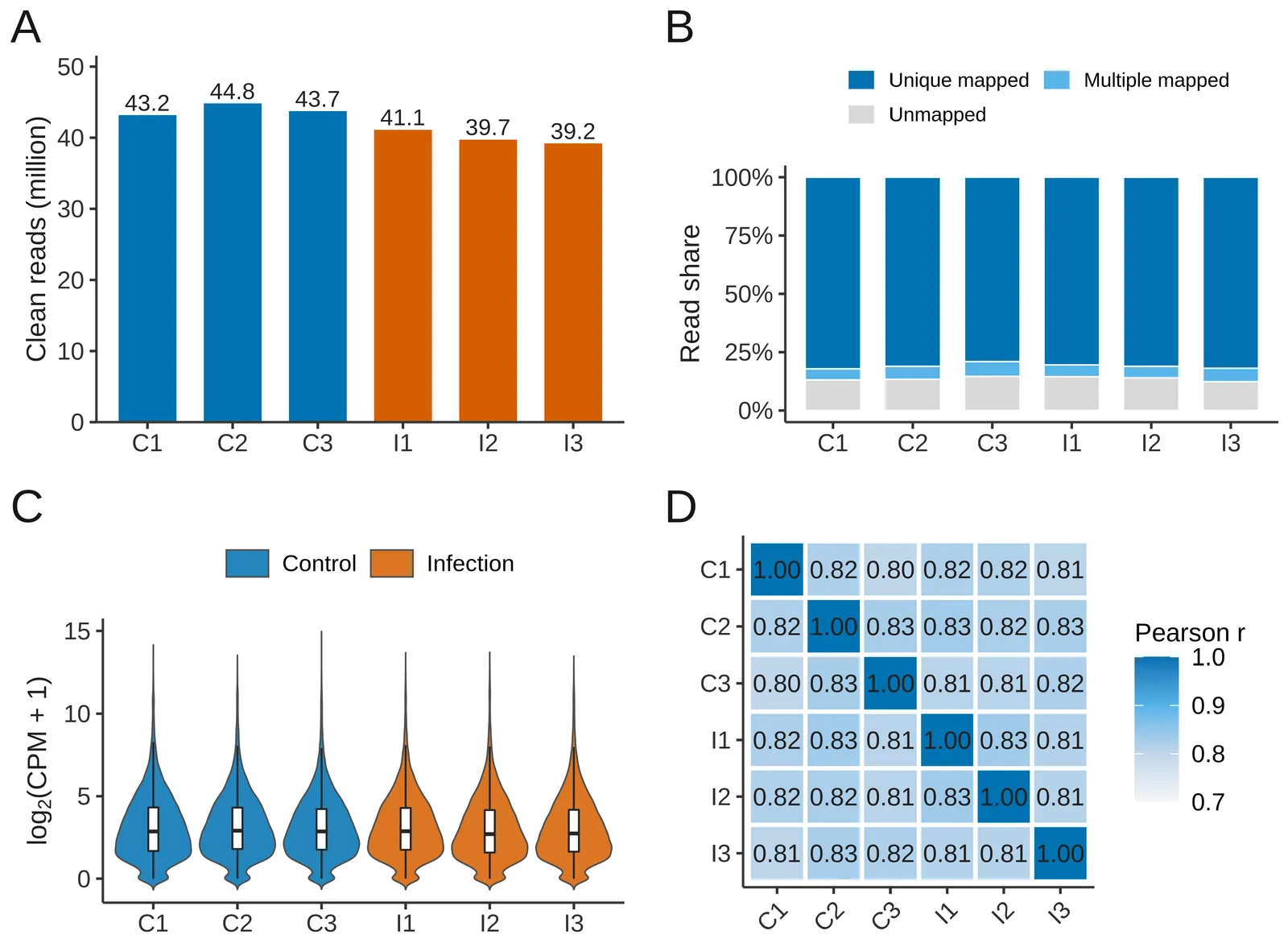
Quality control and sample concordance of the skin RNA-seq dataset. (**A**) Per-sample clean-read yield. (**B**) Proportions of uniquely mapped, multiply mapped and unmapped reads. (**C**) Distribution of log_2_(CPM + 1) values after library-size normalization for transcript entries retained for sample-level analysis. (**D**) Pearson correlation matrix calculated from the same filtered transcript-level log_2_(CPM + 1) matrix. Transcript entries with CPM ≥ 1 in at least three samples were retained, yielding 29,124 entries. Gene-level PCA is shown in Figure S2.

Each RNA-seq library yielded 39.2–44.8 million clean reads, with Q30 values of 95.72–96.20%, GC contents of 44.74–45.14%, total mapping rates of 85.39–87.69%, and unique mapping rates of 79.07–82.15% (Figure 4A,B). Transcript-level Pearson correlations, calculated from 29,124 entries with CPM ≥ 1 in at least three libraries, ranged from 0.800 to 0.835 (Figure 4C,D). Gene-level RSEM correlations ranged from 0.744 to 0.982, with QS_C_3 showing lower concordance with several samples. In the gene-level PCA, PC1 and PC2 explained 26.33% and 21.14% of the variance, respectively, and control and challenged samples overlapped substantially (Figure S2).

### 3.5. FDR-Controlled Analysis Identified 72 Differentially Expressed Genes

**Figure 5:**
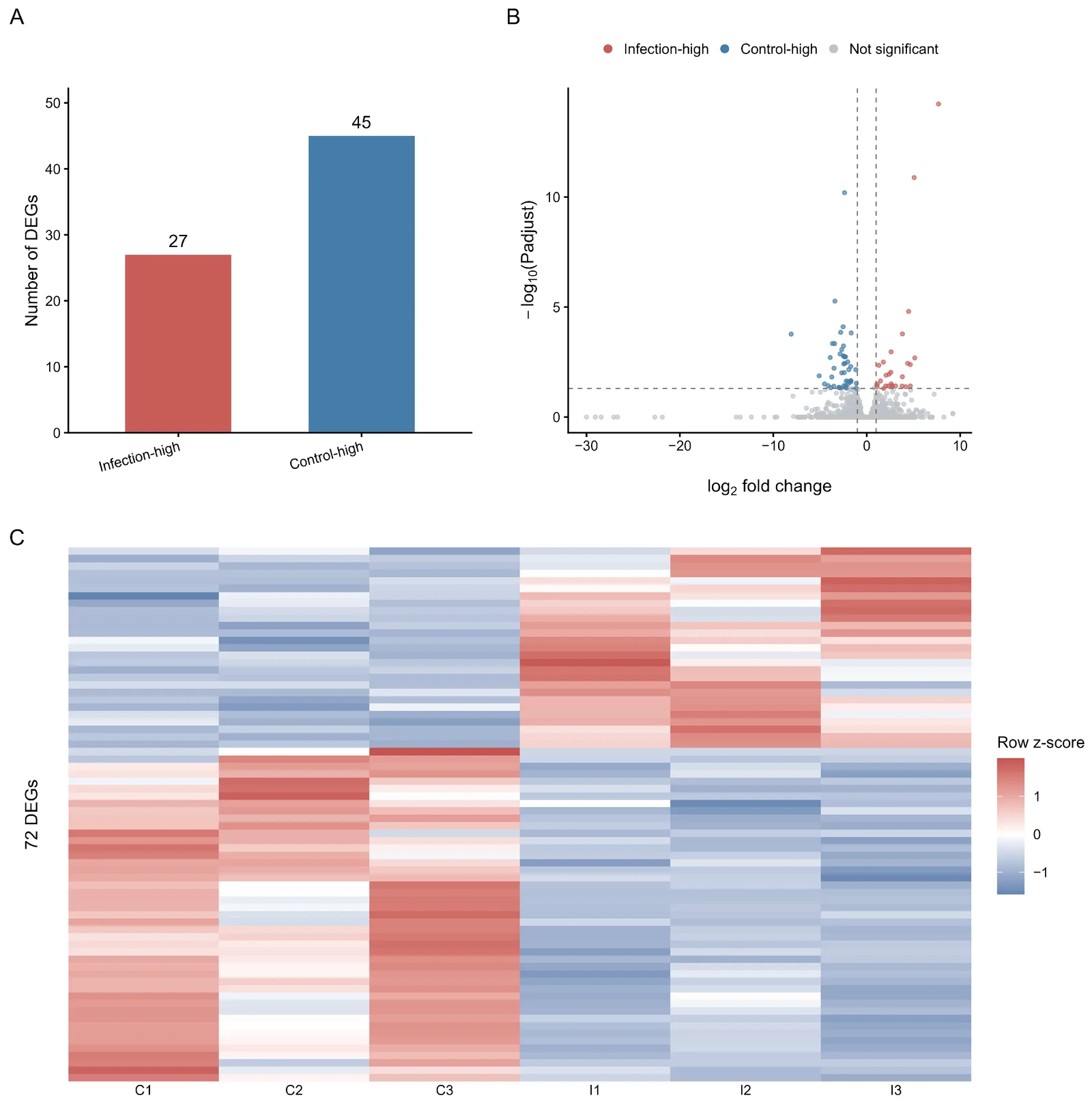
Global differential-expression profile of challenged and control *Quasipaa spinosa* skin after FDR correction. (**A**) Numbers of the 72 DEGs with higher expression in challenged or control skin. (**B**) Volcano plot of the challenged-versus-control comparison; positive log_2_ fold changes indicate higher expression in challenged skin and the y-axis shows −log_10_(padj). (**C**) Row-standardized heatmap of the 72 FDR-defined DEGs. DEGs were defined as padj < 0.05 and |log_2_FC| ≥ 1. In panel B, the two vertical dashed lines indicate log_2_FC thresholds of −1 and +1, while the horizontal dashed line indicates the statistical significance threshold of padj = 0.05. Genes meeting both criteria were considered differentially expressed.

Using padj < 0.05 and |log_2_FC| ≥ 1, 72 genes were differentially expressed between challenged and control skin: 27 had higher expression in challenged skin and 45 in control skin (Figure 5A,B). Figure 5C shows the row-standardized expression profiles of these 72 genes across the six RNA-seq libraries.

### 3.6. Functional Enrichment Remained Limited After FDR Correction

Of the 72 DEGs, 16 had GO annotations and 52 had KEGG annotations in the enrichment analysis. No GO term remained significant after Benjamini–Hochberg correction. Three KEGG annotations remained significant at padj < 0.05 and were driven by six unique DEGs: an *IRF3*-like gene and three *Mx*/*Mx2*-like genes grouped under “Innate Antiviral Response”, and *c-Fos* plus an immunoglobulin heavy-variable-like gene grouped under “Immune Signaling” (Figure S3).

### 3.7. Secretory and Immune-Effector Candidates Showed Heterogeneous Expression

**Figure 6:**
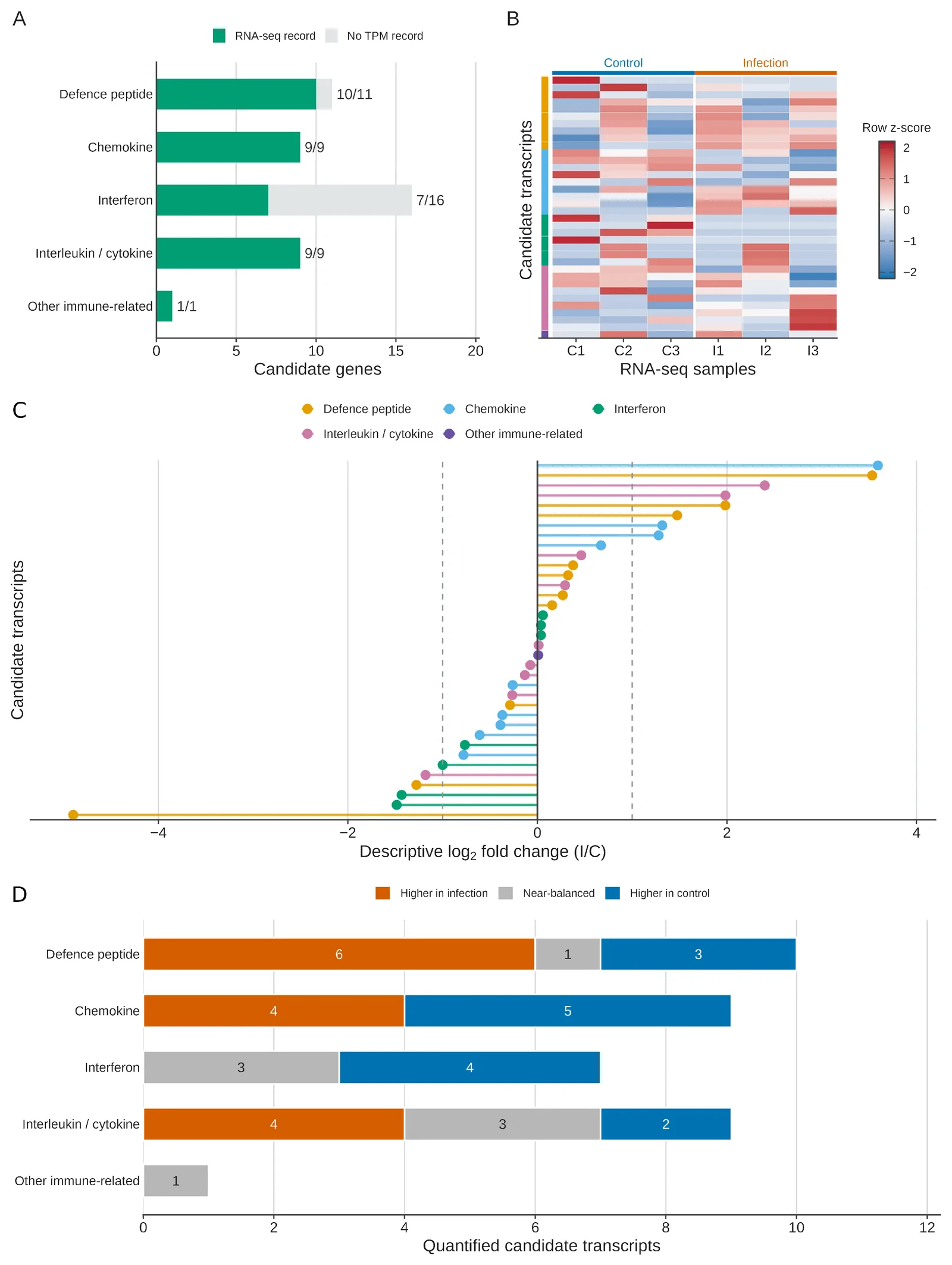
RNA-seq expression profiles of full-length-defined secretory and immune-effector candidates. (**A**) Candidate genes by functional class; green bars show gene representatives with transcript-level TPM records and gray segments show candidate genes without such records, with labels giving RNA-seq-record/total genes. (**B**) Row-standardized log_2_(TPM + 1) heatmap for the 36 gene representatives with transcript-level TPM values. (**C**) Descriptive log_2_ ratio of mean TPM between infection and control groups, calculated as log_2_[(infection mean TPM + 0.1)/(control mean TPM + 0.1)]; The dashed vertical lines indicate descriptive log_2_ ratio values of −1 and +1 and serve as visual guides. (**D**) Candidate counts classified by expression direction. Gene-level differential-expression statistics were available for 29 of these 36 representatives; none of these 29 candidate genes belonged to the genome-wide set of 72 FDR-defined DEGs. Individual representative transcript IDs, annotations, and mean TPM values for panel C are listed in Table S5.

The full-length reference contained 46 secretory and immune-effector genes represented by 66 transcript models and 72 candidate ORFs. One representative transcript–ORF record per gene was used for expression summaries; 36 representatives had transcript-level TPM values, comprising 10 defense-peptide precursors, 9 chemokines, 7 interferons, 9 interleukin/cytokine candidates, and 1 other immune-related candidate (Figure 6A,B; Table 1). Mean TPM was higher in challenged skin for 14 candidates, higher in control skin for 14, and near-balanced (|log_2_ ratio| < 0.25) for 8 (Figure 6C,D).

Gene-level differential-expression statistics were available for 29 of the 36 representatives. The remaining seven were MSTRG loci with gene-level TPM and count data but no corresponding entries in the differential-expression output. None of these 29 candidate genes belonged to the genome-wide set of 72 FDR-defined DEGs identified in Section 3.5. Representative IDs, annotations, and group mean TPM values are provided in Table S5.

### 3.8. Representative Candidate Loci Showed Isoform-Dependent Coding Diversity

**Figure 7:**
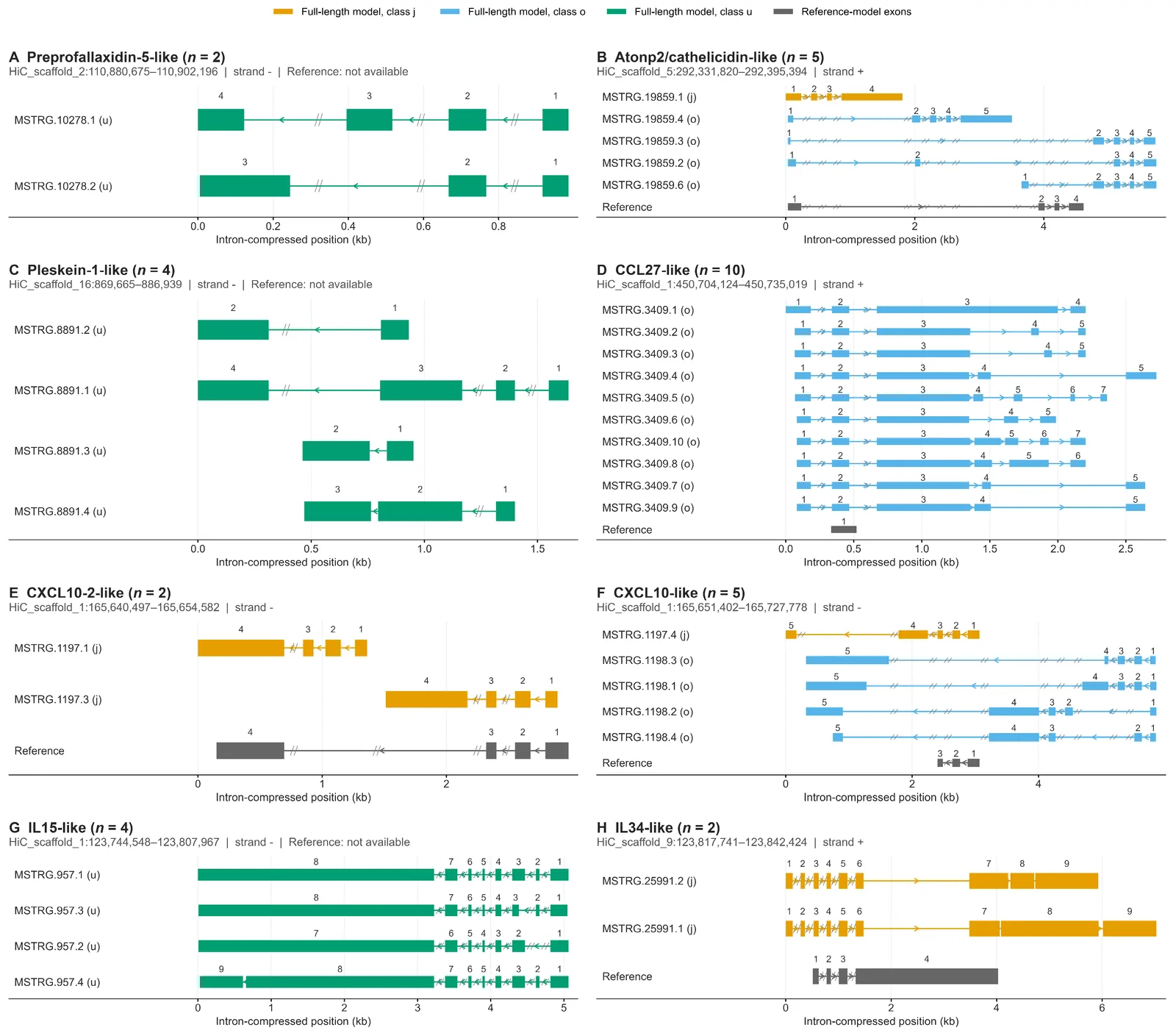
Intron-compressed isoform structures at eight representative candidate loci. (**A**) Preprofallaxidin-5-like (*n* = 2), (**B**) Atonp2/cathelicidin-like (*n* = 5), (**C**) Pleskein-1-like (*n* = 4), (**D**) CCL27-like (*n* = 10), (**E**) CXCL10-2-like (*n* = 2), (**F**) CXCL10-like (*n* = 5), (**G**) IL15-like (*n* = 4), and (**H**) IL34-like (*n* = 2). Here, *n* indicates the number of reconstructed full-length isoforms per locus. Genomic coordinates, strand, exon numbering, stable transcript IDs, and reference models (when available) are shown. Arrows indicate transcription direction, and double slashes indicate compressed intronic regions. Supporting evidence is summarized in Tables S2 and S3.

Eight multi-isoform loci selected using the criteria described in Methods represented defense-peptide, chemokine, and cytokine-related candidates (Figure 7A–H). Together, these loci contained 34 reconstructed isoforms with variable PacBio full-model read and RNA-seq splice-junction support (Table S2). Several loci showed isoform-associated ORF/protein differences, including changes in predicted signal-peptide status (Table S3). At the Atonp2/cathelicidin-like locus (Figure 7B), five reconstructed isoforms were associated with eight ORFs that differed in predicted protein sequence and signal-peptide status; the CXCL10-2-like locus (Figure 7E) contained two reconstructed isoforms with RNA-seq support for all three splice junctions and three ORFs differing in length and SignalP prediction.

### 3.9. Peptidomics Revealed Variable but Recurrent Peptide Detection

**Figure 8:**
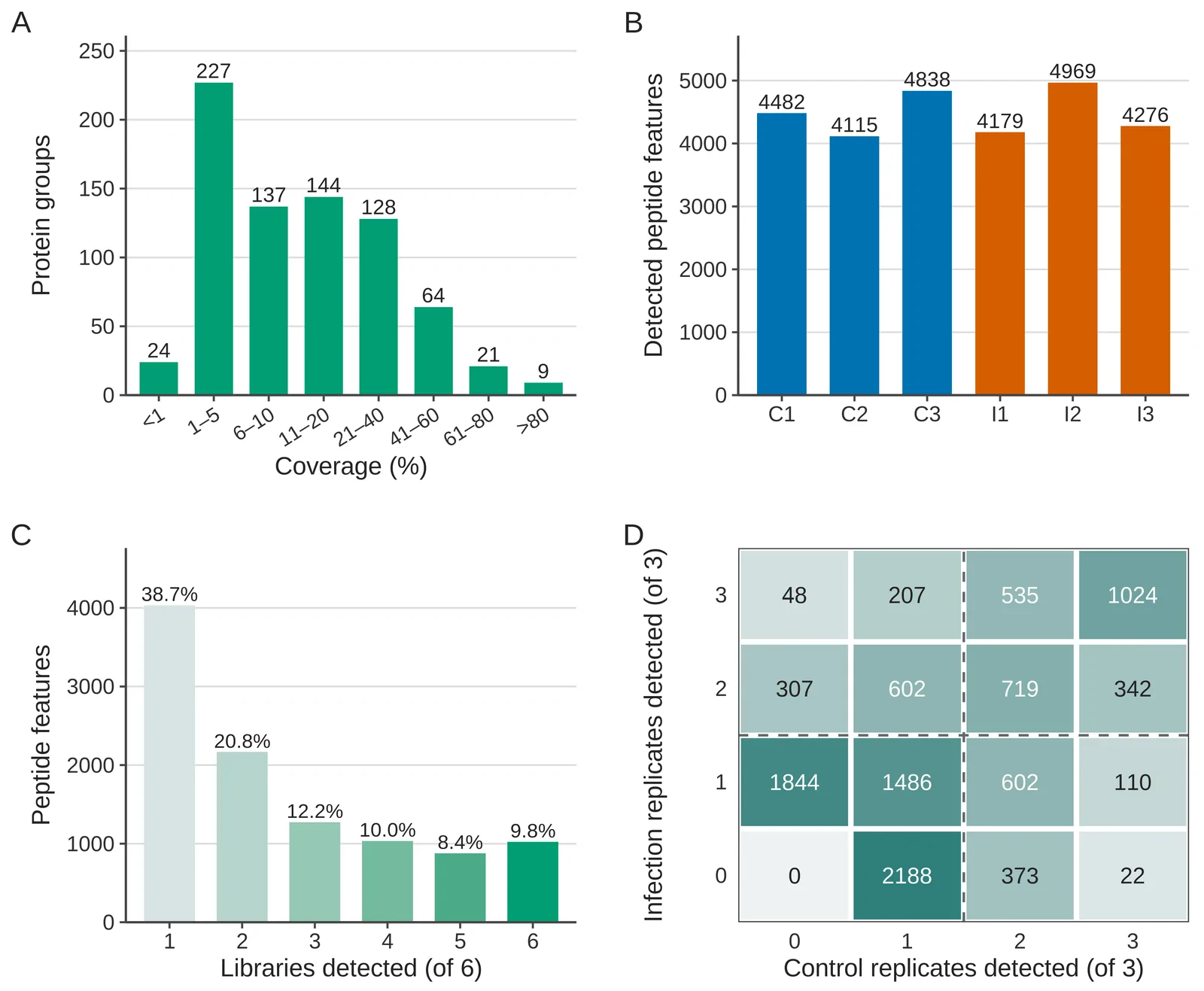
Peptide-level coverage, missingness and repeatable detection in the skin peptidomics dataset. (**A**) Distribution of protein sequence coverage across the 754 protein groups summarized in the peptidomics output. (**B**) Number of peptide-feature rows with quantitative area greater than zero in each library; blue and orange bars represent control and challenged samples, respectively. (**C**) Number and proportion of observed peptide-feature rows detected in one to six libraries. (**D**) Cross-tabulation of the number of control and infection biological replicates with peptide detection; darker shading indicates higher peptide-feature counts. The vertical and horizontal dashed lines indicate the thresholds of detection in at least two of three control and infection replicates, respectively. Features meeting the ≥2/3 threshold in only one condition may still have been detected in one replicate of the other condition. These patterns are descriptive; no differential peptide-abundance test was performed.

Protein-group sequence coverage is shown in Figure 8A. The peptidomics dataset contained 10,933 peptide-feature rows, of which 524 had zero quantitative area in all six libraries. Among the 10,409 detected features, 1024 (9.8%) occurred in all six libraries and 4032 (38.7%) in only one; individual libraries contained 4115–4969 detected features (Figure 8B,C). Using detection in at least two of three biological replicates as the repeatability threshold, 3727 features met the criterion in control skin and 3784 in challenged skin. Of these, 2620 met the criterion in both conditions, 1107 only in control skin, and 1164 only in challenged skin (Figure 8D).

Peptide matches were observed for Atonp2/cathelicidin-like and Preprofallaxidin-5-like candidates; however, neither satisfied both the repeatability and ORF-specificity criteria required for candidate peptide support.

### 3.10. Cross-Omics Integration Linked DEGs to Isoform Structure and Peptide Evidence

**Figure 9:**
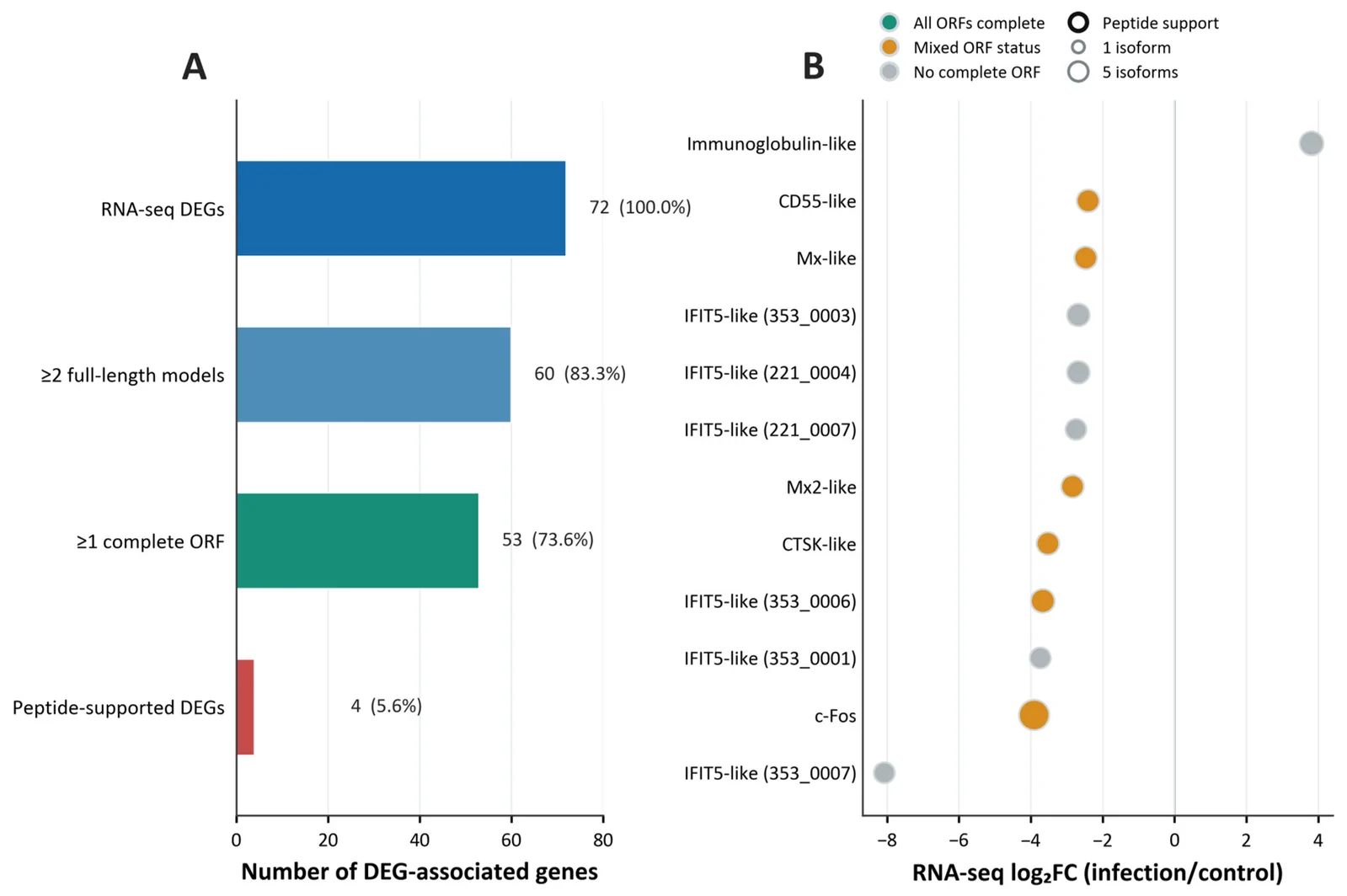
Full-length structural resolution and peptide support among the 72 FDR-defined DEGs. (**A**) Numbers represented by at least two reconstructed full-length models, by at least one complete ORF and by a matched peptide sequence; percentages are relative to the 72-DEG set. Peptide-supported DEGs form an overlap set and are not restricted to genes with a complete ORF. (**B**) Representative annotated DEGs plotted by RNA-seq log_2_FC (challenged/control). Point size indicates the number of reconstructed full-length isoforms, fill indicates ORF-completeness status and a black outline indicates peptide support. DEGs were defined using padj < 0.05 and |log_2_FC| ≥ 1.

Among the 72 FDR-defined DEGs, 60 (83.3%) had at least two reconstructed full-length models, 53 (73.6%) contained at least one complete ORF, and 4 (5.6%) had peptide-sequence support (Figure 9A). Representative DEGs spanned positive and negative RNA-seq log_2_FC values and varied in isoform number and ORF completeness (Figure 9B).

## 4. Discussion

The main contribution of this study is an isoform-resolved skin transcript reference for *Q. spinosa* that can be interpreted together with gene-level RNA-seq and peptidomic evidence. Earlier work in this species identified individual bioactive peptides and described gene-level responses to bacterial infection [12,13,14,15,16,17], but did not resolve the broader set of full-length transcript structures, alternative isoforms, complete ORFs, and candidate precursor architectures present in skin. The present dataset therefore adds a structural layer to existing functional studies and provides a species-specific framework for examining skin-defense genes at transcript and precursor levels.

The full-length catalog also broadens the view of the skin-defense repertoire beyond conventional antimicrobial peptides. We prioritized 72 secretory and immune-effector ORFs from 46 genes, spanning defense-peptide precursors, chemokines, interferons, interleukin/cytokine-related effectors, and other immune-related proteins. Candidate selection combined N-terminal secretion signals with functional annotation, while defense-peptide precursors received additional screening for processing motifs, family homology, and predicted mature-peptide properties. These criteria reduce a large ORF catalog to a tractable set for follow-up, but the candidates remain sequence-based predictions until secretion, precursor processing, and biological activity are experimentally confirmed. This distinction is important because amphibian skin peptides can perform diverse antimicrobial, antiviral, chemotactic, anti-inflammatory, and tissue-protective functions [3,4,5,6,14,15,16].

The RNA-seq data placed this structural resource in the context of a 24 h *P. mirabilis* challenge. After FDR correction, 72 genes met padj < 0.05 and |log_2_FC| ≥ 1, with 27 showing higher expression in challenged skin and 45 showing higher expression in control skin. No GO term remained significant after multiple-testing correction. The three significant KEGG annotations were driven by six unique genes: an *IRF3*-like gene and three Mx/Mx2-like genes grouped under “Innate Antiviral Response”, and c-Fos plus an immunoglobulin heavy-variable-like gene grouped under “Immune Signaling”. Likewise, NF-κB, phagosome, RIG-I-like receptor, TNF, and Toll-like receptor signaling did not remain significant after correction. The transcriptomic response at this time point is therefore better interpreted as limited and gene-specific than as broad activation of canonical immune pathways.

Long-read sequencing added structural information that gene-level expression values alone cannot provide. Among the 72 FDR-defined DEGs, 60 had at least two reconstructed full-length models and 53 contained at least one complete ORF (Figure 9A). The representative loci shown in Figure 7 further illustrate alternative exon organization and coding structures in defense-peptide, chemokine, and cytokine-related genes. Because the Iso-Seq library was pooled across conditions, these models define the available transcript structures but cannot establish condition-specific isoform abundance [20,21,22,23,24,25,26]. Among the 46 prioritized candidate genes, 29 had matching gene-level differential-expression statistics, and none of these 29 candidate genes belonged to the genome-wide set of 72 FDR-defined DEGs. The candidate repertoire and the acute challenge response therefore provide complementary rather than interchangeable information. Some candidates may be constitutively expressed, respond outside the 24 h window, or differ mainly at the level of precursor processing rather than transcript abundance.

Peptidomics provided an additional, more selective layer of support. Peptide sequences matched 1029 entries in the full-length catalog and 4 of the 72 FDR-defined DEGs. Although peptide matches were observed for Atonp2/cathelicidin-like and Preprofallaxidin-5-like candidates, neither met both criteria for candidate peptide support. Peptides mapped to 175 incomplete ORFs also matched complete ORFs, so they were treated as shared sequence evidence rather than proof that the incomplete models were translated. These results show the value of peptide evidence for supporting parts of the transcript catalog, while also illustrating its limited ability to assign every detected sequence to a unique precursor. A peptide match alone does not establish the exact mature product or its biological activity.

Several limitations should be considered when interpreting the dataset. Pooling control and challenged samples for Iso-Seq increased transcript discovery but removed individual- and condition-specific information on isoform abundance. The RNA-seq experiment included three biological replicates per group, and the PCA showed substantial overlap between conditions despite similar sequencing-quality metrics across libraries, indicating appreciable inter-individual variation. Only one post-challenge time point was examined. In addition, a subset of single-exon class-u models had weak short-read support and should remain provisional because residual genomic-DNA-derived artifacts cannot be excluded. Because transcript classification was referenced to the available genome annotation, incomplete or inaccurate reference gene models may affect the assignment of some reconstructed transcripts as known or novel isoforms. Peptide detection is also incomplete and can be influenced by ionization efficiency, sampling depth, and stochastic MS/MS selection [53,54]. Future work with additional time points and biological replicates, isoform-specific transcript assays, targeted peptidomics, synthetic-peptide confirmation, precursor-processing measurements, and functional immune assays will be important for testing the strongest candidates.

## 5. Conclusions

This study provides the first isoform-resolved skin transcript resource for *Q. spinosa* by integrating pooled PacBio Iso-Seq with independent RNA-seq and peptidomics. The full-length catalog substantially expanded transcript structural information and enabled prioritization of 72 secretory and immune-effector ORFs from 46 genes. In the 24 h *P. mirabilis* challenge, FDR-controlled RNA-seq identified 72 differentially expressed genes, but functional enrichment was limited, indicating a modest gene-level response at this time point rather than broad pathway-wide activation. Long-read sequencing added isoform and ORF resolution to most of these genes, whereas peptide matches provided sequence-level support for a smaller subset. Together, these complementary datasets distinguish transcript structure, gene-level expression, and peptide detection as separate evidence layers and provide a practical framework for prioritizing candidates for follow-up. This resource should facilitate isoform-specific validation, characterization of peptide precursor processing, targeted peptidomics, and functional studies of amphibian skin defense and disease resistance in aquaculture.

## Figures and Tables

**Table 1:** Representative transcript–ORF records for 36 secretory and immune-effector candidate genes with available transcript-level TPM values and peptide-support information.

Candidate Annotation	Stable IDs	Effector Class	Candidate ORFs	Infection Mean TPM	Control Mean TPM	Peptide Observation and Support Status
Preprofallaxidin-5-like	Gene: MSTRG.10278 Transcript: MSTRG.10278.1 ORF: ORF027052	Defense-peptide precursor	2	3088.17	781.46	VLDKVTDL; detected in 6/6 libraries (3/3 control, 3/3 challenged); shared/non-ORF-specific; does not meet candidate-support criteria
Temporin-H-like	Gene: MSTRG.9310 Transcript: MSTRG.9310.1 ORF: ORF105960	Defense-peptide precursor	1	1135.89	98.10	None reported
Pleskein-1-like	Gene: MSTRG.9309 Transcript: MSTRG.9309.1 ORF: ORF105958	Defense-peptide precursor	1	37.88	91.97	None reported
*Atonp2*/cathelicidin-like	Gene: QSP_Contig828_0015 Transcript: QSP_Contig828_0015 ORF: ORF013948	Defense-peptide precursor	8	22.46	27.48	STSHIGSIDPLSSHGAQQ; detected in 1/6 libraries (0/3 control, 1/3 challenged); ORF-specific but below the repeatability threshold; does not meet candidate-support criteria
Beta-defensin	Gene: QSP_Contig950_0023 Transcript: MSTRG.12214.1 ORF: ORF033395	Defense-peptide precursor	1	47.08	42.27	None reported
Pleskein-1-like	Gene: MSTRG.8891 Transcript: MSTRG.8891.1 ORF: ORF104205	Defense-peptide precursor	1	21.65	7.73	None reported
Temporin-H-like	Gene: A17034 Transcript: MSTRG.8894.2 ORF: ORF104211	Defense-peptide precursor	1	5.67	4.69	None reported
Pleskein-3-like	Gene: MSTRG.8892 Transcript: MSTRG.8892.1 ORF: ORF104207	Defense-peptide precursor	1	5.58	4.44	None reported
Hepcidin-like	Gene: QSP_Contig1211_0003 Transcript: QSP_Contig1211_0003 ORF: ORF021800	Defense-peptide precursor	1	0.00	2.88	None reported
Cystatin-F-like	Gene: QSP_Contig300_0067 Transcript: MSTRG.17202.1 ORF: ORF050918	Defense-peptide precursor	1	0.79	0.58	None reported
C-C motif chemokine 20-like	Gene: QSP_Contig662_0017 Transcript: QSP_Contig662_0017 ORF: ORF011898	Chemokine	1	51.74	32.46	None reported
C-X-C motif chemokine 14	Gene: QSP_Contig312_0001 Transcript: MSTRG.14523.1 ORF: ORF041573	Chemokine	2	24.95	32.28	None reported
C-C motif chemokine 27	Gene: QSP_Contig106_0004 Transcript: MSTRG.3409.1 ORF: ORF085145	Chemokine	1	7.74	9.29	None reported
C-X-C motif chemokine 10-like	Gene: QSP_Contig571_0043 Transcript: QSP_Contig571_0043 ORF: ORF002907	Chemokine	1	6.81	2.75	None reported
C-X-C motif chemokine 10-2	Gene: QSP_Contig571_0044 Transcript: MSTRG.1197.1 ORF: ORF032579	Chemokine	2	4.30	7.46	None reported
C-X-C motif chemokine 10-3	Gene: QSP_Contig33_0001 Transcript: MSTRG.1198.1 ORF: ORF032612	Chemokine	4	3.92	5.17	None reported
C-C motif chemokine 21-like	Gene: MSTRG.3402 Transcript: MSTRG.3402.1 ORF: ORF085085	Chemokine	1	1.44	0.52	None reported
C-C motif chemokine 19-like	Gene: QSP_Contig106_0002 Transcript: MSTRG.3404.1 ORF: ORF085087	Chemokine	3	1.83	2.85	None reported
C-C motif chemokine 2-like	Gene: QSP_Contig662_0018 Transcript: QSP_Contig662_0018 ORF: ORF011899	Chemokine	1	1.11	0.00	None reported
Interferon-gamma-like	Gene: QSP_Contig240_0035 Transcript: QSP_Contig240_0035 ORF: ORF008195	Interferon	1	0.75	0.72	None reported
Interferon lambda-3-like	Gene: QSP_Contig256_0002 Transcript: QSP_Contig256_0002 ORF: ORF001646	Interferon	1	0.00	0.18	None reported
Interferon lambda-3-like	Gene: QSP_Contig256_0006 Transcript: QSP_Contig256_0006 ORF: ORF001650	Interferon	1	0.00	0.17	None reported
Interferon lambda-3-like	Gene: QSP_Contig823_0004 Transcript: QSP_Contig823_0004 ORF: ORF003371	Interferon	1	0.00	0.10	None reported
Interferon lambda-3-like	Gene: QSP_Contig256_0003 Transcript: QSP_Contig256_0003 ORF: ORF001647	Interferon	1	0.00	0.07	None reported
Interferon lambda-3-like	Gene: QSP_Contig256_0004 Transcript: QSP_Contig256_0004 ORF: ORF001648	Interferon	1	0.03	0.03	None reported
Interferon lambda-3-like	Gene: QSP_Contig823_0002 Transcript: QSP_Contig823_0002 ORF: ORF003369	Interferon	1	0.03	0.03	None reported
Interleukin-17D-like	Gene: QSP_Contig48_0001 Transcript: MSTRG.7020.1 ORF: ORF097513	Interleukin/cytokine	2	68.72	56.12	None reported
Interleukin-8-like	Gene: QSP_Contig29_0012 Transcript: MSTRG.1218.1 ORF: ORF033281	Interleukin/cytokine	2	11.28	8.16	None reported
Interleukin-17A/F1alpha-like	Gene: A29000 Transcript: MSTRG.15746.2 ORF: ORF046116	Interleukin/cytokine	1	7.39	1.79	None reported
Interleukin-15-like	Gene: MSTRG.957 Transcript: MSTRG.957.1 ORF: ORF106524	Interleukin/cytokine	4	2.97	6.87	None reported
Interleukin-17D	Gene: QSP_Contig160_0003 Transcript: MSTRG.11767.2 ORF: ORF032009	Interleukin/cytokine	3	3.70	4.06	None reported
Pro-interleukin-16	Gene: QSP_Contig211_0027 Transcript: MSTRG.13371.1 ORF: ORF038004	Interleukin/cytokine	2	1.33	1.41	None reported
Unnamed protein product	Gene: QSP_Contig988_0005 Transcript: MSTRG.25991.1 ORF: ORF082298	Interleukin/cytokine	2	1.28	1.56	None reported
Interleukin-10-like	Gene: QSP_Contig531_0024 Transcript: QSP_Contig531_0024 ORF: ORF005748	Interleukin/cytokine	1	0.80	0.07	None reported
Cytokine-like protein 1	Gene: QSP_Contig674_0013 Transcript: QSP_Contig674_0013 ORF: ORF003147	Interleukin/cytokine	1	0.29	0.29	None reported
T-cell immunomodulatory protein	Gene: QSP_Contig466_0030 Transcript: MSTRG.25693.4 ORF: ORF080970	Other immune-related	3	1.13	1.12	None reported

Note: TPM values are mean transcript-level abundances across three biological replicates per condition for the representative transcript. “Candidate ORFs” indicates the number of prioritized ORFs assigned to each gene. Peptide detection frequency is the number of libraries with quantitative area > 0; shared peptides were not treated as candidate-specific support. “None reported” indicates no linked peptide feature. Candidates with mean TPM < 1 were retained as sequence-based candidates; such low transcript abundance should not be interpreted as evidence of strong biological expression or activity. For prioritized candidates, peptide support required repeatable detection (≥2/3 biological replicates in at least one condition) and ORF specificity. Peptide observations failing either criterion were not counted as candidate peptide support.

## Data Availability

The raw PacBio Iso-Seq and Illumina RNA-seq data generated in this study have been deposited in the NCBI Sequence Read Archive (SRA) under BioProject PRJNA1517185. The corresponding BioSample accessions are SAMN62639665–SAMN62639671, and the SRA run accessions are SRR40306512–SRR40306518. The peptidomics data generated in this study have been deposited in the iProX repository under project accession IPX0019989000.

## Supplementary Materials

The following supporting information can be downloaded at https://www.mdpi.com/article/10.3390/biology15201802/s1: Figure S1: Iso-Seq transcript rarefaction analysis; Figure S2: Principal component analysis of gene-level RNA-seq expression profiles; Figure S3: Functional grouping of six unique DEGs associated with significant KEGG annotations; Table S1: Iso-Seq quality-control, read-support, SQANTI3 classification, RNA-seq splice-junction support, and rarefaction summary; Table S2: PacBio full-model read support and RNA-seq splice-junction support for the 34 transcript models shown in Figure 7; Table S3: Selection of representative Figure 7 loci and within-locus ORF, protein-sequence, and SignalP 6.0 comparisons; Table S4: Peptide and PSM evidence for sequences matching incomplete ORFs; Table S5: Representative transcript IDs, candidate annotations, sample-level TPM values, group mean TPM values, descriptive expression ratios, and available gene-level differential-expression statistics underlying Figure 6C.
