# Supplementary material for: Integrated Full-Length Transcriptomics, RNA-Seq and Peptidomics Define an Isoform-Resolved Skin Defense Repertoire in the Chinese Spiny Frog (Quasipaa spinosa)
Source: Biology (Basel). 2026 Oct 9;15(20):1802. doi: 10.3390/biology15201802 (PMC13701283; doi:10.3390/biology15201802)
Supplement: Supplementary file 1 [file biology-15-01802-s001.zip › biology-4564974-supplementary.pdf]

Supplementary Materials

Integrated Full-Length Transcriptomics, RNA-seq and Peptidomics Define an Isoform-Resolved Skin Defense Repertoire in the Chinese Spiny Frog (*Quasipaa spinosa*)

Jie Chen, Yikai Feng, Xinyi Qian and Zhihua Lin

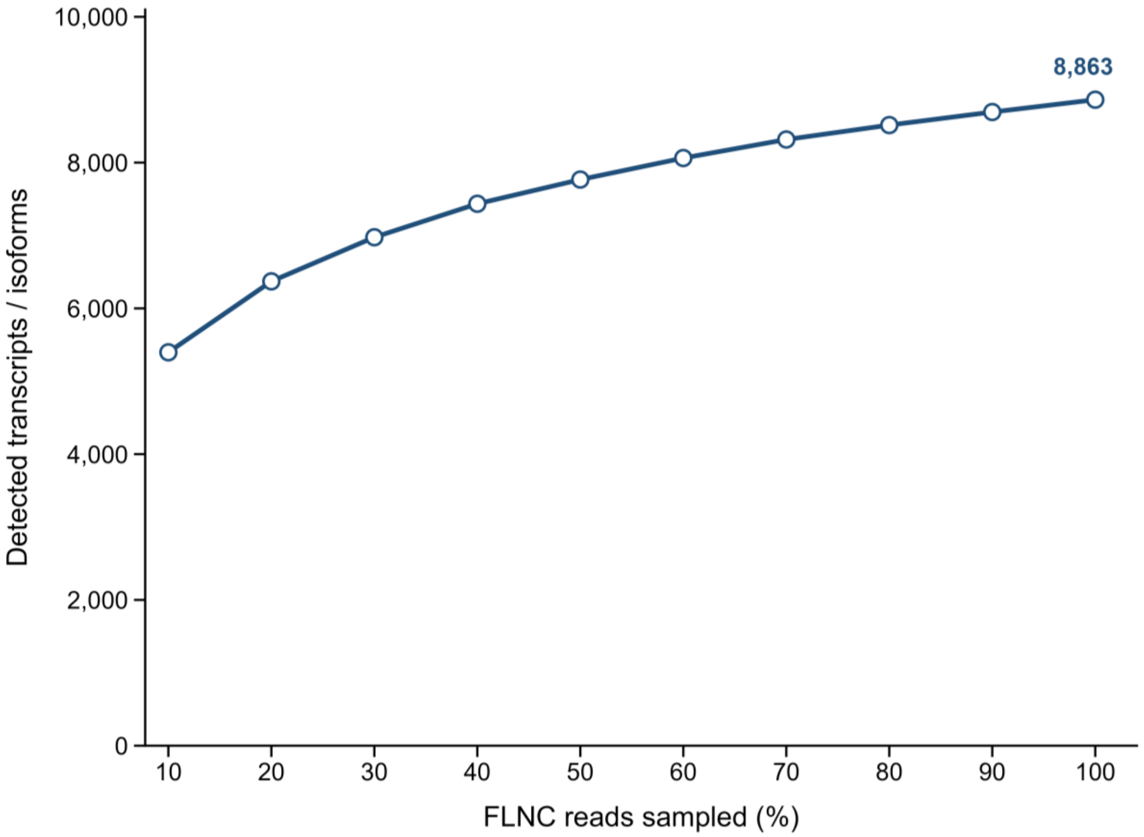

Figure S1. Iso-Seq transcript rarefaction analysis. Refined poly(A)+ FLNC reads were randomly subsampled from 10% to 100% of the total dataset, with three independent random subsamplings at each depth. A transcript was considered detected when supported by at least one uniquely assigned full-model FLNC read. The number of detected isoforms increased from an average of 5,397 at 10% sequencing depth to 8,863 at the full dataset, with the curve progressively approaching saturation at higher sampling depths.

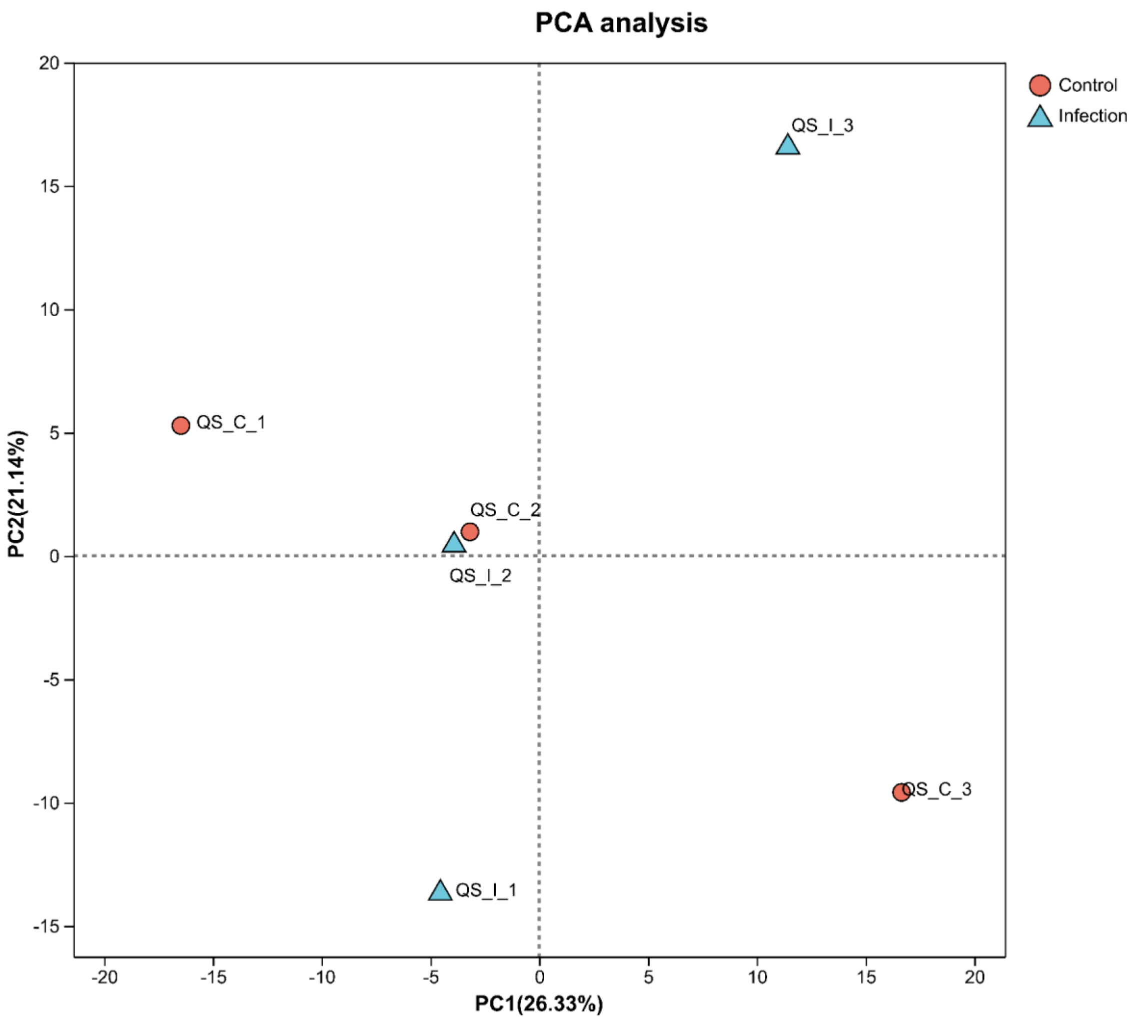

Figure S2. Principal component analysis of gene-level RNA-seq expression profiles. PCA was performed using gene-level RSEM expression data from three control and three infection-group skin samples. PC1 and PC2 explained 26.33% and 21.14% of the total variance, respectively. The two groups showed substantial overlap, indicating limited condition-based separation and appreciable inter-individual variation.

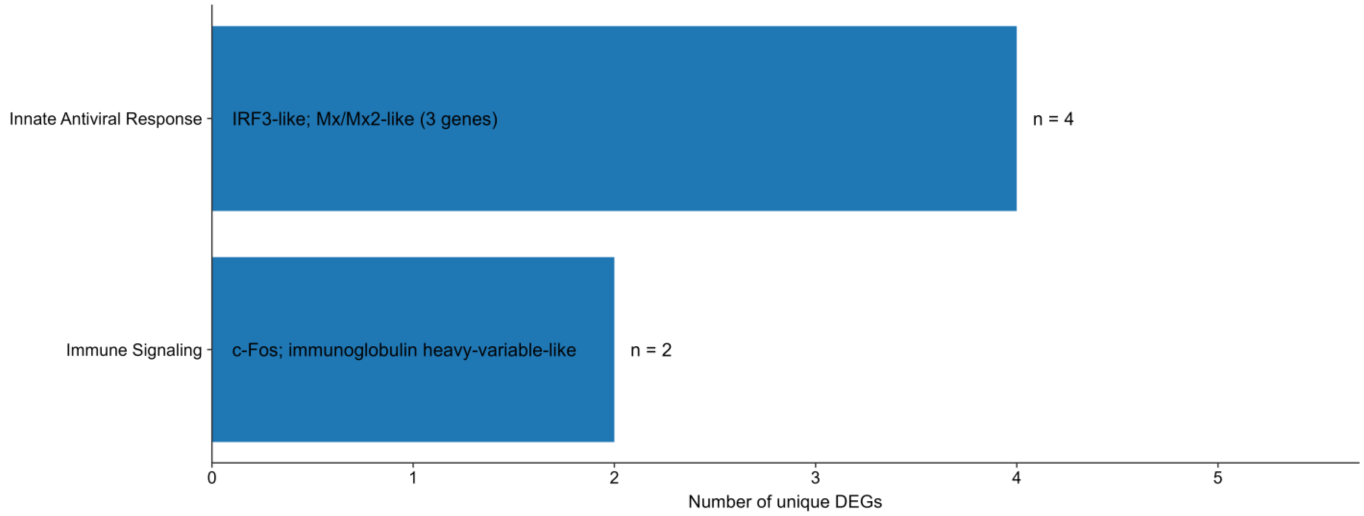

Figure S3. Functional grouping of six unique DEGs associated with significant KEGG annotations. The IRF3-like gene and three Mx/Mx2-like genes were grouped as Innate Antiviral Response, whereas c-Fos and an immunoglobulin heavy-variable-like gene were grouped as Immune Signaling.

Table S1. Iso-Seq quality-control, read-support, SQANTI3 classification, RNA-seq splice-junction support, and rarefaction summary.

QC metric	Result
FLNC reads	10,622,861
Clustered/polished consensus sequences	599,697
Consensus sequences mapped to reference genome	581,683 (97.00%)
Uniquely mapped consensus sequences	94.80%
5′/3′ primer-supported FLNC reads	10,654,125 / 10,660,747 (99.94%)
poly(A)+ FLNC reads (≥20 nt) among primer-supported FLNC reads	10,645,929 (99.92%)
Final transcript models with ≥1 unique full-model read	8,863 / 58,388 (15.18%)
Final transcript models with ≥2 unique full-model reads	7,359 / 58,388 (12.60%)
Final transcript models with ≥3 unique full-model reads	6,534 / 58,388 (11.19%)
Final transcript models with ≥5 unique full-model reads	5,596 / 58,388 (9.58%)
SQANTI3-classified transcript models	58,207 / 58,388; 181 unclassified/unmapped
SQANTI3 full-splice match (FSM)	20,475
SQANTI3 incomplete-splice match (ISM)	976
SQANTI3 novel-in-catalog (NIC)	4,517
SQANTI3 novel-not-in-catalog (NNC)	14,741
SQANTI3 intergenic / antisense / genic / genic-intron	11,291 / 3,658 / 1,512 / 1,037
Canonical splice-junction records	369,757 / 374,726 (98.67%)
Multi-exon models with ≥1 RNA-seq-supported junction	37,728 / 46,462 (81.20%)
Multi-exon models with all junctions RNA-seq-supported	22,847 / 46,462 (49.17%)
Models retained by standard SQANTI3 rules after RNA-seq junction coverage	46,652 / 58,207 classified models (80.15%)
Models flagged by standard SQANTI3 rules	11,555 / 58,207 classified models (19.85%)
Rarefaction: mean detected isoforms at 10% / 50% / 100% depth	5,397 / 7,767 / 8,863

Table S2. PacBio full-model read support and RNA-seq splice-junction support for the 34 transcript models shown in Figure 7.

Locus	Transcript ID	Class	Exons	Length (nt)	Unique full-model reads	Compatible full-model reads	RNA-seq junctions supported/total	Minimum junction coverage	Samples with junction support
Preprofallaxidin-5-like	MSTRG.10278.1	u	4	414	4	137	2/3	0	6
Preprofallaxidin-5-like	MSTRG.10278.2	u	3	409	0	145	2/2	846	6
Atonp2/cathelicidin-like	MSTRG.19859.1	j	4	1360	0	0	3/3	245	6
Atonp2/cathelicidin-like	MSTRG.19859.2	o	5	571	0	0	4/4	245	6
Atonp2/cathelicidin-like	MSTRG.19859.3	o	5	554	0	0	3/4	0	6
Atonp2/cathelicidin-like	MSTRG.19859.4	o	5	1179	64	64	4/4	109	6
Atonp2/cathelicidin-like	MSTRG.19859.6	o	5	640	0	0	4/4	10	6
Pleskein-1-like	MSTRG.8891.1	u	4	845	0	0	2/3	0	6
Pleskein-1-like	MSTRG.8891.2	u	2	435	0	0	0/1	0	0
Pleskein-1-like	MSTRG.8891.3	u	2	414	1	1	0/1	0	0
Pleskein-1-like	MSTRG.8891.4	u	3	748	0	0	1/2	0	6
CCL27-like	MSTRG.3409.1	o	4	1751	3	7	3/3	14	6
CCL27-like	MSTRG.3409.10	o	7	1379	0	2	5/6	0	6
CCL27-like	MSTRG.3409.2	o	5	1040	0	0	3/4	0	6
CCL27-like	MSTRG.3409.3	o	5	1040	0	0	3/4	0	6
CCL27-like	MSTRG.3409.4	o	5	1242	0	0	4/4	12	6
CCL27-like	MSTRG.3409.5	o	7	1135	0	0	5/6	0	6
CCL27-like	MSTRG.3409.6	o	5	1128	0	0	3/4	0	6
CCL27-like	MSTRG.3409.7	o	5	1113	0	1	4/4	7	6
CCL27-like	MSTRG.3409.8	o	6	1443	0	5	4/5	0	6
CCL27-like	MSTRG.3409.9	o	5	1177	0	3	4/4	12	6
CXCL10-2-like	MSTRG.1197.1	j	4	1001	24	24	3/3	126	6
CXCL10-2-like	MSTRG.1197.3	j	4	965	15	16	3/3	152	6
CXCL10-like	MSTRG.1197.4	j	5	1032	0	1	2/4	0	5
CXCL10-like	MSTRG.1198.1	o	5	1700	0	0	3/4	0	6
CXCL10-like	MSTRG.1198.2	o	5	1709	0	0	4/4	14	6
CXCL10-like	MSTRG.1198.3	o	5	1691	0	0	3/4	0	6
CXCL10-like	MSTRG.1198.4	o	5	1275	0	0	4/4	11	6
IL15-like	MSTRG.957.1	u	8	4013	52	120	7/7	212	6
IL15-like	MSTRG.957.2	u	7	3960	0	74	6/6	159	6
IL15-like	MSTRG.957.3	u	8	3909	0	44	7/7	91	6
IL15-like	MSTRG.957.4	u	9	3944	0	26	7/8	0	6
IL34-like	MSTRG.25991.1	j	9	4157	0	0	6/8	0	6
IL34-like	MSTRG.25991.2	j	9	3090	0	0	6/8	0	6

Note: “Unique full-model reads” are conservative isoform-specific assignments using the criteria described in Section 2.5. “Compatible full-model reads” also include full-model-compatible assignments that are not uniquely attributable to one isoform. RNA-seq junction support is aggregated across the six STAR-aligned libraries. A zero under the strict PacBio criterion means that no read passed that specific assignment rule and does not by itself establish transcript absence.

Table S3. Selection of representative Figure 7 loci and within-locus ORF, protein-sequence, and SignalP 6.0 comparisons.

Locus	Stratum	Rank	Isoforms	Matched ORFs	ORF lengths (aa)	Unique protein sequences	SignalP results	Cleavage sites	ORF-length change	Protein-sequence change	Signal-peptide change
Preprofallaxidin-5-like	Defense-peptide	3	2	2	74	1	SP	22-23	No	No	No
Atonp2/cathelicidin-like	Defense-peptide	1	5	8	50;91;145;154	7	OTHER;SP	20-21;NA	Yes	Yes	Yes
Pleskein-1-like	Defense-peptide	2	4	2	75;78	2	SP	22-23;24-25	Yes	Yes	No
CCL27-like	Chemokine	1	10	20	61;95;96;107;111;125;128;130;132;217;235;314	12	OTHER;SP	23-24;35-36;39-40;NA	Yes	Yes	Yes
CXCL10-2-like	Chemokine	3	2	3	52;100;101	3	OTHER;SP	20-21;21-22;NA	Yes	Yes	Yes
CXCL10-like	Chemokine	2	5	6	60;109;110;143	5	OTHER;SP	20-21;NA	Yes	Yes	Yes
IL15-like	Interleukin/cytokine	1	4	10	49;53;156	3	OTHER;SP	21-22;NA	Yes	Yes	Yes
IL34-like	Interleukin/cytokine	2	2	8	52;117;187;227;303;305	6	OTHER;SP	17-18;NA	Yes	Yes	Yes

Note: Eligible loci contained ≥2 reconstructed MSTRG full-length isoforms and ≥1 complete ORF. Loci were ranked within functional strata using the criteria stated in Section 2.6; the top three defense-peptide, three chemokine, and two interleukin/cytokine loci were retained. Protein-sequence differences are summarized from within-locus MAFFT alignments. SignalP was run on matched ORFs; two Pleskein-1-like isoforms without matched ORFs were retained as NA rather than inferred.

Table S4. Peptide and PSM evidence for sequences matching incomplete ORFs.

Table S4 is presented in two parts for readability: Table S4A summarizes evidence at the incomplete-ORF level, and Table S4B summarizes evidence at the unique-peptide level.

All peptide/PSM identifications passed the 1% FDR filter, and the database search used a fragment mass tolerance of 0.05 Da. All peptide sequences listed here also matched at least one complete ORF and therefore were not interpreted as incomplete-ORF-specific translation evidence.

Table S4A. ORF-level summary of peptide/PSM evidence.

ORF ID	Transcript ID	Gene ID	Completeness	Peptides (n)	PSM associations (n)	Samples (n)	Complete ORF match(es)	Best −10LgP	Max ∣ppm∣
ORF027075	MSTRG.10286.1	QSP_Contig1585_0001	5prime_partial	4	7	4	ORF004248	47.43	6.30
ORF027076	MSTRG.10286.2	QSP_Contig1585_0001	5prime_partial	4	7	4	ORF004248	47.43	6.30
ORF027079	MSTRG.10286.3	QSP_Contig1585_0001	5prime_partial	4	7	4	ORF004248	47.43	6.30
ORF027326	MSTRG.10364.1	QSP_Contig531_0021	3prime_partial	4	12	5	ORF005745	46.62	11.90
ORF027357	MSTRG.1038.2	QSP_Contig294_0031	5prime_partial	3	10	5	ORF001771	47.46	9.00
ORF029551	MSTRG.11097.1	QSP_Contig340_0015	5prime_partial	2	2	1	ORF005045; ORF029552; ORF029553; ORF029555; ORF029563; ORF029564; ORF029566	35.52	4.20
ORF029554	MSTRG.11097.13	QSP_Contig340_0015	5prime_partial	1	1	1	ORF005045; ORF029552; ORF029553; ORF029555; ORF029563; ORF029564; ORF029566	35.52	4.20
ORF029559	MSTRG.11097.2	QSP_Contig340_0014	internal	1	3	3	ORF005044	46.13	3.70
ORF029560	MSTRG.11097.3	QSP_Contig340_0015	5prime_partial	2	2	1	ORF005045; ORF029552; ORF029553; ORF029555; ORF029563; ORF029564; ORF029566	35.52	4.20
ORF029561	MSTRG.11097.4	QSP_Contig340_0015	5prime_partial	2	2	1	ORF005045; ORF029552; ORF029553; ORF029555; ORF029563; ORF029564; ORF029566	35.52	4.20
ORF029562	MSTRG.11097.5	QSP_Contig340_0015	5prime_partial	2	2	1	ORF005045; ORF029552; ORF029553; ORF029555; ORF029563; ORF029564; ORF029566	35.52	4.20
ORF031536	MSTRG.11679.2	QSP_Contig1546_0006	5prime_partial	41	151	6	ORF004221	47.46	18.30
ORF031542	MSTRG.11679.3	QSP_Contig1546_0006	5prime_partial	39	142	6	ORF004221	47.46	18.30
ORF032526	MSTRG.11946.4	QSP_Contig293_0021	5prime_partial	4	17	6	ORF004880; ORF005253	46.66	15.40
ORF033209	MSTRG.1212.1	QSP_Contig29_0009	5prime_partial	5	28	6	ORF001812	46.26	6.20
ORF036238	MSTRG.12913.1	QSP_Contig250_0021	5prime_partial	1	3	2	ORF008268	46.51	6.80
ORF036244	MSTRG.12913.2	QSP_Contig250_0021	5prime_partial	1	3	2	ORF008268	46.51	6.80
ORF036252	MSTRG.12913.3	QSP_Contig250_0021	5prime_partial	1	3	2	ORF008268	46.51	6.80

ORF ID	Transcript ID	Gene ID	Completeness	Peptides (n)	PSM associations (n)	Samples (n)	Complete ORF match(es)	Best −10LgP	Max ∣ppm∣
ORF036644	MSTRG.12994.1	QSP_Contig266_0052	5prime_partial	8	14	4	ORF008344; ORF036655; ORF036666; ORF036670; ORF036673	46.93	18.60
ORF036647	MSTRG.12994.2	QSP_Contig266_0052	5prime_partial	8	14	4	ORF008344; ORF036655; ORF036666; ORF036670; ORF036673	46.93	18.60
ORF038388	MSTRG.13483.2	QSP_Contig113_0037	5prime_partial	1	1	1	ORF007177; ORF038385; ORF038389	46.81	6.60
ORF038393	MSTRG.13483.4	QSP_Contig113_0037	5prime_partial	1	1	1	ORF007177; ORF038385; ORF038389	46.81	6.60
ORF038565	MSTRG.13549.1	QSP_Contig200_0003	5prime_partial	2	2	1	ORF007809	43.08	4.10
ORF038836	MSTRG.1365.4	QSP_Contig148_0029	5prime_partial	2	4	3	ORF001160; ORF038831; ORF038834	46.64	14.30
ORF040982	MSTRG.14314.2	QSP_Contig594_0007	3prime_partial	20	137	6	ORF008943; ORF022249	47.45	14.20
ORF041048	MSTRG.1435.1	QSP_Contig944_0013	5prime_partial	52	342	6	ORF003580	47.45	15.10
ORF043012	MSTRG.14883.3	QSP_Contig179_0059	3prime_partial	2	6	4	ORF007631; ORF043008	45.99	11.20
ORF043114	MSTRG.14915.5	QSP_Contig179_0094	5prime_partial	1	1	1	ORF007666; ORF043103; ORF043107; ORF043110	34.02	5.20
ORF044453	MSTRG.15203.1	QSP_Contig424_0018	3prime_partial	23	60	6	ORF008679; ORF008680; ORF008677	47.46	14.70
ORF044627	MSTRG.15257.1	QSP_Contig825_0027	5prime_partial	9	19	5	ORF009505; ORF022326; ORF092996; ORF093003; ORF011313	43.77	8.50
ORF045937	MSTRG.15676.1	QSP_Contig661_0009	3prime_partial	1	5	4	ORF011267; ORF011880	45.13	5.20
ORF045938	MSTRG.15676.2	QSP_Contig394_0001	3prime_partial	1	5	4	ORF011267; ORF011880	45.13	5.20
ORF045939	MSTRG.15676.3	QSP_Contig661_0009	3prime_partial	1	5	4	ORF011267; ORF011880	45.13	5.20
ORF045940	MSTRG.15676.4	QSP_Contig661_0009	3prime_partial	1	5	4	ORF011267; ORF011880	45.13	5.20
ORF045941	MSTRG.15676.5	QSP_Contig394_0001	3prime_partial	1	5	4	ORF011267; ORF011880	45.13	5.20
ORF045942	MSTRG.15676.6	QSP_Contig661_0009	internal	1	5	4	ORF011267; ORF011880	45.13	5.20
ORF045943	MSTRG.15676.8	QSP_Contig661_0009	5prime_partial	1	5	4	ORF011267; ORF011880	45.13	5.20
ORF050024	MSTRG.16879.1	QSP_Contig2_0062	5prime_partial	2	4	2	ORF010954	38.81	2.00
ORF050709	MSTRG.17139.1	QSP_Contig156_0008	3prime_partial	7	11	4	ORF009765; ORF010196; ORF037399; ORF037400	47.16	5.80

ORF ID	Transcript ID	Gene ID	Completeness	Peptides (n)	PSM associations (n)	Samples (n)	Complete ORF match(es)	Best −10LgP	Max ∣ppm∣
ORF051130	MSTRG.17271.1	QSP_Contig951_0006	5prime_partial	4	8	4	ORF012252	47.46	7.30
ORF051137	MSTRG.17271.2	QSP_Contig951_0006	5prime_partial	4	8	4	ORF012252	47.46	7.30
ORF052086	MSTRG.17566.3	QSP_Contig914_0019	5prime_partial	1	1	1	ORF012186; ORF052076	46.93	4.40
ORF053166	MSTRG.1782.2	QSP_Contig649_0014	5prime_partial	7	21	6	ORF003049; ORF053159; ORF053173; ORF053180	47.34	9.60
ORF053999	MSTRG.18074.10	QSP_Contig20_0004	5prime_partial	31	84	6	ORF012810; ORF054010; ORF054012; ORF054001; ORF054006; ORF054013; ORF054018	47.46	16.00
ORF055592	MSTRG.18550.1	QSP_Contig37_0070	5prime_partial	3	5	2	ORF013351	32.31	3.70
ORF057182	MSTRG.19053.1	QSP_Contig1414_0006	5prime_partial	8	25	6	ORF012631; ORF057183	47.39	15.60
ORF059178	MSTRG.19589.3	QSP_Contig362_0053	5prime_partial	2	3	3	ORF013234; ORF059172; ORF059175	37.62	12.50
ORF060338	MSTRG.1991.10	QSP_Contig456_0041	5prime_partial	22	53	6	ORF002404; ORF060337; ORF060344; ORF060345; ORF060349; ORF060350; ORF060353; ORF060354; ORF060356; ORF060365; ORF000781	47.26	18.80
ORF060339	MSTRG.1991.11	QSP_Contig127_0030	5prime_partial	1	1	1	ORF000781; ORF002404; ORF060337; ORF060344; ORF060345; ORF060349; ORF060350; ORF060353; ORF060354; ORF060356	39.65	3.70
ORF060340	MSTRG.1991.12	QSP_Contig127_0030	5prime_partial	1	1	1	ORF000781; ORF002404; ORF060337; ORF060344; ORF060345; ORF060349; ORF060350; ORF060353; ORF060354; ORF060356	39.65	3.70
ORF060352	MSTRG.1991.20	QSP_Contig127_0030	3prime_partial	1	1	1	ORF000781; ORF002404; ORF060337; ORF060344; ORF060345; ORF060349; ORF060350; ORF060353; ORF060354; ORF060356	39.65	3.70
ORF060355	MSTRG.1991.23	QSP_Contig127_0030	3prime_partial	1	1	1	ORF000781; ORF002404; ORF060337; ORF060344; ORF060345; ORF060349; ORF060350; ORF060353; ORF060354; ORF060356	39.65	3.70
ORF060357	MSTRG.1991.25	QSP_Contig127_0030	5prime_partial	1	1	1	ORF000781; ORF002404; ORF060337; ORF060344; ORF060345; ORF060349; ORF060350; ORF060353; ORF060354; ORF060356	39.65	3.70
ORF060358	MSTRG.1991.26	QSP_Contig127_0030	5prime_partial	1	1	1	ORF000781; ORF002404; ORF060337; ORF060344; ORF060345; ORF060349; ORF060350; ORF060353; ORF060354; ORF060356	39.65	3.70
ORF060364	MSTRG.1991.3	QSP_Contig456_0041	5prime_partial	22	53	6	ORF002404; ORF060337; ORF060344; ORF060345; ORF060349; ORF060350; ORF060353; ORF060354; ORF060356; ORF060365; ORF000781	47.26	18.80
ORF060367	MSTRG.1991.4	QSP_Contig127_0030	5prime_partial	1	1	1	ORF000781; ORF002404; ORF060337; ORF060344; ORF060345; ORF060349; ORF060350; ORF060353; ORF060354; ORF060356	39.65	3.70
ORF060370	MSTRG.1991.6	QSP_Contig127_0030	5prime_partial	1	1	1	ORF000781; ORF002404; ORF060337; ORF060344; ORF060345; ORF060349; ORF060350; ORF060353; ORF060354; ORF060356	39.65	3.70
ORF060373	MSTRG.1991.7	QSP_Contig456_0041	5prime_partial	2	2	1	ORF002404; ORF060337; ORF060344; ORF060345; ORF060349; ORF060350; ORF060353; ORF060354; ORF060356	33.01	5.10
ORF060374	MSTRG.1991.8	QSP_Contig127_0030	5prime_partial	1	1	1	ORF000781; ORF002404; ORF060337; ORF060344; ORF060345; ORF060349; ORF060350; ORF060353; ORF060354; ORF060356	39.65	3.70
ORF060375	MSTRG.1991.9	QSP_Contig127_0030	5prime_partial	1	1	1	ORF000781; ORF002404; ORF060337; ORF060344; ORF060345; ORF060349; ORF060350; ORF060353; ORF060354; ORF060356	39.65	3.70

ORF ID	Transcript ID	Gene ID	Completeness	Peptides (n)	PSM associations (n)	Samples (n)	Complete ORF match(es)	Best −10LgP	Max |ppm|
ORF060786	MSTRG.19993.4	QSP_Contig1265_0007	5prime_partial	1	1	1	ORF014594; ORF060773; ORF060779; ORF060783	31.52	9.60
ORF060787	MSTRG.19993.5	QSP_Contig1265_0007	5prime_partial	1	1	1	ORF014594; ORF060773; ORF060779; ORF060783	31.52	9.60
ORF061770	MSTRG.20291.3	QSP_Contig63_0119	5prime_partial	8	11	6	ORF015690; ORF061765; ORF061767	46.44	15.00
ORF062015	MSTRG.20378.2	QSP_Contig63_0021	5prime_partial	4	4	1	ORF015592	47.40	11.20
ORF062221	MSTRG.20441.10	QSP_Contig298_0049	3prime_partial	217	773	6	ORF014920	47.46	17.40
ORF062223	MSTRG.20441.11	QSP_Contig298_0049	3prime_partial	217	773	6	ORF014920	47.46	17.40
ORF062226	MSTRG.20441.4	QSP_Contig298_0049	3prime_partial	217	773	6	ORF014920	47.46	17.40
ORF062227	MSTRG.20441.5	QSP_Contig298_0049	3prime_partial	217	773	6	ORF014920	47.46	17.40
ORF062229	MSTRG.20441.6	QSP_Contig298_0049	3prime_partial	217	773	6	ORF014920	47.46	17.40
ORF062232	MSTRG.20441.7	QSP_Contig298_0049	3prime_partial	217	773	6	ORF014920	47.46	17.40
ORF062233	MSTRG.20441.8	QSP_Contig298_0049	3prime_partial	217	773	6	ORF014920	47.46	17.40
ORF062236	MSTRG.20441.9	QSP_Contig298_0049	3prime_partial	217	773	6	ORF014920	47.46	17.40
ORF063827	MSTRG.20960.2	QSP_Contig43_0007	5prime_partial	2	6	5	ORF015212	46.08	15.30
ORF065205	MSTRG.2131.3	QSP_Contig136_0013	5prime_partial	2	2	1	ORF000973; ORF065202	44.90	4.70
ORF065206	MSTRG.2131.4	QSP_Contig136_0013	5prime_partial	2	2	1	ORF000973; ORF065202	44.90	4.70
ORF065585	MSTRG.21444.1	QSP_Contig568_0023	5prime_partial	1	1	1	ORF015511	34.10	5.50
ORF065586	MSTRG.21444.2	QSP_Contig568_0023	5prime_partial	1	1	1	ORF015511	34.10	5.50
ORF065587	MSTRG.21444.3	QSP_Contig568_0023	5prime_partial	1	1	1	ORF015511	34.10	5.50
ORF066694	MSTRG.21758.10	QSP_Contig1154_0014	3prime_partial	1	1	1	ORF016200; ORF066716	41.63	3.00
ORF066708	MSTRG.21758.5	QSP_Contig1154_0014	5prime_partial	2	12	6	ORF016200; ORF066693; ORF066716	47.03	8.50
ORF066774	MSTRG.21795.1	QSP_Contig1034_0010	5prime_partial	1	3	3	ORF016089	36.40	5.10

ORF ID	Transcript ID	Gene ID	Completeness	Peptides (n)	PSM associations (n)	Samples (n)	Complete ORF match(es)	Best −10LgP	Max ∣ppm∣
ORF066779	MSTRG.21795.4	QSP_Contig1034_0010	5prime_partial	1	3	3	ORF016089	36.40	5.10
ORF066964	MSTRG.21844.1	QSP_Contig155_0033	5prime_partial	6	9	4	ORF010381; ORF016315; ORF005131; ORF012718	40.99	4.30
ORF067090	MSTRG.21890.1	QSP_Contig305_0037	5prime_partial	29	108	6	ORF016564; ORF006342	47.46	13.60
ORF067172	MSTRG.21919.1	QSP_Contig305_0012	3prime_partial	23	60	6	ORF008470; ORF016539; ORF020361; ORF020365; ORF020604; ORF020626; ORF020632; ORF020636; ORF020638; ORF020641; ORF020717; ORF020720; ORF020908; ORF020913; ORF020951; ORF020954; ORF021008; ORF021012; ORF021015; ORF021020; ORF021411; ORF021497; ORF021500; ORF021528; ORF021529; ORF021532; ORF021534; ORF021542; ORF021563; ORF021731; ORF041549; ORF085760; ORF025126; ORF026134; ORF004026; ORF005986; ORF021386; ORF021537; ORF003297; ORF005988; ORF008392; ORF029575; ORF029578; ORF036876	47.44	11.70
ORF068100	MSTRG.22136.1	QSP_Contig755_0009	5prime_partial	1	1	1	ORF016152; ORF017153	40.65	1.20
ORF068995	MSTRG.22469.1	QSP_Contig309_0027	5prime_partial	1	10	6	ORF016023; ORF016608	44.67	4.80
ORF069000	MSTRG.22469.3	QSP_Contig309_0027	5prime_partial	1	10	6	ORF016023; ORF016608	44.67	4.80
ORF069006	MSTRG.22469.5	A36391	5prime_partial	1	10	6	ORF016023; ORF016608	44.67	4.80
ORF069010	MSTRG.22469.6	A36391	5prime_partial	1	10	6	ORF016023; ORF016608	44.67	4.80
ORF069012	MSTRG.22469.7	A36391	5prime_partial	1	10	6	ORF016023; ORF016608	44.67	4.80
ORF069609	MSTRG.2266.5	QSP_Contig151_0011	5prime_partial	4	8	3	ORF001221; ORF069605; ORF069606; ORF069607; ORF069608	42.69	5.20
ORF069950	MSTRG.22763.1	QSP_Contig429_0019	5prime_partial	19	101	6	ORF016799; ORF069954; ORF069958; ORF069960	47.46	10.60
ORF071696	MSTRG.23183.1	QSP_Contig259_0022	5prime_partial	42	197	6	ORF018184	47.46	9.60
ORF072258	MSTRG.23322.1	QSP_Contig1920_0010	5prime_partial	1	1	1	ORF018021	34.75	5.30
ORF072259	MSTRG.23322.3	QSP_Contig1920_0010	5prime_partial	1	1	1	ORF018021	34.75	5.30
ORF072898	MSTRG.23529.10	QSP_Contig176_0125	5prime_partial	5	11	6	ORF017928; ORF072897; ORF072901; ORF072902; ORF072903; ORF072904; ORF072905	47.45	14.10
ORF072899	MSTRG.23529.11	QSP_Contig176_0125	5prime_partial	5	11	6	ORF017928; ORF072897; ORF072901; ORF072902; ORF072903; ORF072904; ORF072905	47.45	14.10
ORF072900	MSTRG.23529.12	QSP_Contig176_0125	5prime_partial	5	11	6	ORF017928; ORF072897; ORF072901; ORF072902; ORF072903; ORF072904; ORF072905	47.45	14.10
ORF072906	MSTRG.23529.8	QSP_Contig176_0125	5prime_partial	5	11	6	ORF017928; ORF072897; ORF072901; ORF072902; ORF072903; ORF072904; ORF072905	47.45	14.10
ORF072907	MSTRG.23529.9	QSP_Contig176_0125	5prime_partial	5	11	6	ORF017928; ORF072897; ORF072901; ORF072902; ORF072903; ORF072904; ORF072905	47.45	14.10

ORF ID	Transcript ID	Gene ID	Completeness	Peptides (n)	PSM associations (n)	Samples (n)	Complete ORF match(es)	Best −10LgP	Max ǀppmǀ
ORF073021	MSTRG.23562.1	QSP_Contig176_0082	5prime_partial	9	15	3	ORF017885	47.11	19.80
ORF076172	MSTRG.24392.1	QSP_Contig586_0021	5prime_partial	1	4	4	ORF018463	37.29	2.50
ORF076177	MSTRG.24392.2	QSP_Contig586_0021	5prime_partial	1	4	4	ORF018463	37.29	2.50
ORF076196	MSTRG.24397.2	QSP_Contig586_0029	5prime_partial	3	3	1	ORF018471; ORF076197; ORF076198	32.44	7.80
ORF076580	MSTRG.24520.3	QSP_Contig582_0004	5prime_partial	6	20	5	ORF018429; ORF076577; ORF076579; ORF076582	47.46	10.70
ORF076584	MSTRG.24520.5	QSP_Contig582_0004	5prime_partial	12	30	5	ORF018429; ORF076582; ORF076577; ORF076579	47.46	11.50
ORF078118	MSTRG.24857.1	QSP_Contig1234_0005	5prime_partial	1	5	5	ORF019298; ORF078128; ORF078131; ORF078132	47.32	10.60
ORF078133	MSTRG.24857.6	QSP_Contig1234_0005	5prime_partial	1	5	5	ORF019298; ORF078128; ORF078131; ORF078132	47.32	10.60
ORF078612	MSTRG.25035.1	QSP_Contig137_0017	5prime_partial	32	263	6	ORF019339; ORF078617; ORF078626; ORF078629; ORF078634	47.46	19.10
ORF079333	MSTRG.25185.1	QSP_Contig7_0001	5prime_partial	1	1	1	ORF020230	41.27	3.70
ORF079438	MSTRG.25222.1	QSP_Contig7_0036	3prime_partial	24	100	6	ORF020265; ORF079440	47.42	9.70
ORF079444	MSTRG.25222.4	QSP_Contig7_0036	3prime_partial	24	100	6	ORF020265; ORF079440	47.42	9.70
ORF080397	MSTRG.25481.1	QSP_Contig174_0010	5prime_partial	9	15	4	ORF019503	47.42	7.90
ORF080398	MSTRG.25482.1	QSP_Contig174_0012	5prime_partial	2	2	2	ORF019505	41.33	5.10
ORF081161	MSTRG.25719.1	QSP_Contig263_0038	5prime_partial	4	15	6	ORF019667	46.63	6.20
ORF082494	MSTRG.2628.1	QSP_Contig150_0003	5prime_partial	3	7	3	ORF001027; ORF001195; ORF005506; ORF005510; ORF011164; ORF107843; ORF107844; ORF107845; ORF107846; ORF107847; ORF107848; ORF107849; ORF107850; ORF107851; ORF107852; ORF107853; ORF107854; ORF107855; ORF107856; ORF107857; ORF107858; ORF107859; ORF107860	45.19	10.00
ORF082495	MSTRG.2628.2	QSP_Contig150_0003	5prime_partial	3	7	3	ORF001027; ORF001195; ORF005506; ORF005510; ORF011164; ORF107843; ORF107844; ORF107845; ORF107846; ORF107847; ORF107848; ORF107849; ORF107850; ORF107851; ORF107852; ORF107853; ORF107854; ORF107855; ORF107856; ORF107857; ORF107858; ORF107859; ORF107860	45.19	10.00
ORF082496	MSTRG.2628.3	QSP_Contig150_0003	5prime_partial	3	7	3	ORF001027; ORF001195; ORF005506; ORF005510; ORF011164; ORF107843; ORF107844; ORF107845; ORF107846; ORF107847; ORF107848; ORF107849; ORF107850; ORF107851; ORF107852; ORF107853; ORF107854; ORF107855; ORF107856; ORF107857; ORF107858; ORF107859; ORF107860	45.19	10.00
ORF082497	MSTRG.2628.4	QSP_Contig150_0003	5prime_partial	3	7	3	ORF001027; ORF001195; ORF005506; ORF005510; ORF011164; ORF107843; ORF107844; ORF107845; ORF107846; ORF107847; ORF107848; ORF107849; ORF107850; ORF107851; ORF107852; ORF107853; ORF107854; ORF107855; ORF107856; ORF107857; ORF107858; ORF107859; ORF107860	45.19	10.00

ORF ID	Transcript ID	Gene ID	Completeness	Peptides (n)	PSM associations (n)	Samples (n)	Complete ORF match(es)	Best −10LgP	Max ∣ppm∣
ORF082498	MSTRG.2628.5	QSP_Contig150_0003	5prime_partial	3	7	3	ORF001027; ORF001195; ORF005506; ORF005510; ORF011164; ORF107843; ORF107844; ORF107845; ORF107846; ORF107847; ORF107848; ORF107849; ORF107850; ORF107851; ORF107852; ORF107853; ORF107854; ORF107855; ORF107856; ORF107857; ORF107858; ORF107859; ORF107860	45.19	10.00
ORF083443	MSTRG.2901.1	QSP_Contig326_0024	5prime_partial	9	32	6	ORF001845	47.44	17.10
ORF083445	MSTRG.2901.3	QSP_Contig326_0024	3prime_partial	42	112	6	ORF001845	47.46	14.70
ORF083446	MSTRG.2901.4	QSP_Contig326_0024	internal	29	81	6	ORF001845	47.42	13.40
ORF085010	MSTRG.337.1	QSP_Contig329_0008	5prime_partial	1	1	1	ORF001870; ORF085013; ORF085017	43.53	4.60
ORF085019	MSTRG.337.5	QSP_Contig329_0008	3prime_partial	4	11	5	ORF001870; ORF085013; ORF085017	47.42	6.40
ORF086771	MSTRG.4019.1	QSP_Contig182_0008	5prime_partial	1	8	6	ORF020965	47.09	10.10
ORF086801	MSTRG.4026.6	QSP_Contig182_0009	5prime_partial	4	6	3	ORF020966; ORF086789; ORF086800; ORF086783	43.53	8.90
ORF086823	MSTRG.4037.2	QSP_Contig182_0023	5prime_partial	4	6	2	ORF020980; ORF086821	45.94	7.10
ORF086999	MSTRG.4101.1	QSP_Contig221_0022	5prime_partial	2	6	4	ORF021062	46.77	7.00
ORF087579	MSTRG.4251.3	QSP_Contig559_0033	5prime_partial	6	20	6	ORF021453	44.87	8.10
ORF087614	MSTRG.4259.2	QSP_Contig559_0019	3prime_partial	1	1	1	ORF021439	33.04	3.10
ORF087621	MSTRG.4263.1	QSP_Contig559_0016	5prime_partial	8	27	6	ORF021436	47.40	17.90
ORF087623	MSTRG.4263.2	QSP_Contig559_0016	5prime_partial	8	27	6	ORF021436	47.40	17.90
ORF087796	MSTRG.4311.4	QSP_Contig373_0034	5prime_partial	2	6	6	ORF021301; ORF087775; ORF087778; ORF087823	47.38	10.80
ORF087805	MSTRG.4311.6	QSP_Contig373_0034	5prime_partial	2	6	6	ORF021301; ORF087775; ORF087778; ORF087823	47.38	10.80
ORF087831	MSTRG.4311.9	QSP_Contig373_0034	5prime_partial	3	7	6	ORF021301; ORF087775; ORF087778; ORF087823	47.38	10.80
ORF088248	MSTRG.4424.1	QSP_Contig31_0044	5prime_partial	5	15	5	ORF021197	43.54	7.50
ORF088249	MSTRG.4424.3	QSP_Contig31_0044	5prime_partial	5	15	5	ORF021197	43.54	7.50
ORF088463	MSTRG.4479.1	QSP_Contig383_0009	5prime_partial	4	7	2	ORF021312	46.27	8.30
ORF089382	MSTRG.4730.6	QSP_Contig1009_0005	5prime_partial	41	115	6	ORF021680; ORF089369; ORF089372; ORF089373; ORF089377; ORF089379; ORF089383	47.46	16.70

ORF ID	Transcript ID	Gene ID	Completeness	Peptides (n)	PSM associations (n)	Samples (n)	Complete ORF match(es)	Best −10LgP	Max ǀppmǀ
ORF090968	MSTRG.5154.1	QSP_Contig128_0046	5prime_partial	15	42	4	ORF021892	47.46	6.30
ORF091776	MSTRG.5413.2	QSP_Contig1097_0007	5prime_partial	36	177	6	ORF021747; ORF022702	47.46	17.40
ORF091777	MSTRG.5413.3	QSP_Contig1097_0007	5prime_partial	36	177	6	ORF021747; ORF022702	47.46	17.40
ORF091780	MSTRG.5418.2	QSP_Contig766_0008	3prime_partial	1	1	1	ORF017086; ORF022708; ORF068763; ORF098038	32.98	5.20
ORF095365	MSTRG.6418.5	QSP_Contig513_0093	5prime_partial	6	10	5	ORF024066; ORF095359; ORF095363; ORF095366	44.31	11.20
ORF095859	MSTRG.6533.1	QSP_Contig303_0001	5prime_partial	31	121	6	ORF023722; ORF013742; ORF058081; ORF058082	47.45	10.20
ORF097179	MSTRG.6961.1	QSP_Contig303_0069	5prime_partial	2	2	2	ORF023790	44.29	6.20
ORF097181	MSTRG.6961.3	QSP_Contig303_0069	5prime_partial	2	2	2	ORF023790	44.29	6.20
ORF098458	MSTRG.7285.1	QSP_Contig1222_0016	5prime_partial	29	135	6	ORF023230	47.45	17.60
ORF100055	MSTRG.7748.2	QSP_Contig477_0063	5prime_partial	11	26	6	ORF025240; ORF100065	47.11	7.70
ORF100073	MSTRG.7748.4	QSP_Contig477_0063	5prime_partial	10	24	6	ORF025240; ORF100065	47.11	7.70
ORF100075	MSTRG.7748.5	QSP_Contig477_0063	3prime_partial	14	32	6	ORF025240; ORF100065	47.26	7.70
ORF100076	MSTRG.7748.5	QSP_Contig477_0063	5prime_partial	8	21	6	ORF025240; ORF100065	47.11	7.70
ORF100077	MSTRG.7748.6	QSP_Contig477_0063	internal	6	18	6	ORF025240; ORF100065	47.11	7.70
ORF100078	MSTRG.7748.7	QSP_Contig477_0063	internal	10	25	6	ORF025240; ORF100065	47.26	7.70
ORF100079	MSTRG.7748.8	QSP_Contig477_0063	internal	9	22	6	ORF025240; ORF100065	47.26	7.70
ORF100266	MSTRG.7815.1	QSP_Contig1422_0001	5prime_partial	24	87	6	ORF024560	47.46	19.40
ORF100267	MSTRG.7815.2	QSP_Contig1422_0001	5prime_partial	24	87	6	ORF024560	47.46	19.40
ORF100268	MSTRG.7815.3	QSP_Contig1422_0001	5prime_partial	23	86	6	ORF024560	47.46	19.40
ORF100296	MSTRG.7824.4	QSP_Contig277_0013	3prime_partial	6	10	5	ORF024865; ORF100294; ORF100295	46.95	12.00
ORF100297	MSTRG.7824.5	QSP_Contig277_0013	3prime_partial	6	10	5	ORF024865; ORF100294; ORF100295	46.95	12.00

ORF ID	Transcript ID	Gene ID	Completeness	Peptides (n)	PSM associations (n)	Samples (n)	Complete ORF match(es)	Best −10LgP	Max ∣ppm∣
ORF100298	MSTRG.7824.6	QSP_Contig277_0013	3prime_partial	6	10	5	ORF024865; ORF100294; ORF100295	46.95	12.00
ORF100299	MSTRG.7824.7	QSP_Contig277_0013	3prime_partial	6	10	5	ORF024865; ORF100294; ORF100295	46.95	12.00
ORF100300	MSTRG.7824.8	QSP_Contig277_0013	3prime_partial	6	10	5	ORF024865; ORF100294; ORF100295	46.95	12.00
ORF100927	MSTRG.8028.3	QSP_Contig650_0002	5prime_partial	1	2	1	ORF025409; ORF100925; ORF100933	34.92	9.30
ORF101777	MSTRG.8311.1	QSP_Contig609_0015	5prime_partial	38	195	6	ORF025356	47.44	17.00
ORF101779	MSTRG.8311.2	QSP_Contig609_0015	5prime_partial	38	195	6	ORF025356	47.44	17.00
ORF101782	MSTRG.8311.3	QSP_Contig609_0015	5prime_partial	38	195	6	ORF025356	47.44	17.00
ORF101784	MSTRG.8311.4	QSP_Contig609_0015	5prime_partial	38	195	6	ORF025356	47.44	17.00
ORF102745	MSTRG.8538.1	QSP_Contig1439_0006	5prime_partial	10	50	6	ORF024572; ORF000815; ORF008634	47.46	10.20
ORF102746	MSTRG.8538.2	QSP_Contig1439_0006	5prime_partial	10	50	6	ORF024572; ORF000815; ORF008634	47.46	10.20
ORF103626	MSTRG.8790.4	QSP_Contig867_0062	5prime_partial	5	6	1	ORF025742; ORF103624; ORF103625; ORF020600; ORF020628; ORF020724; ORF021390; ORF025739; ORF026119; ORF026136; ORF103631; ORF103655; ORF103657; ORF103659	44.36	10.80
ORF103627	MSTRG.8790.5	QSP_Contig867_0062	internal	1	1	1	ORF020600; ORF020628; ORF020724; ORF021390; ORF025739; ORF025742; ORF026119; ORF026136; ORF103624; ORF103625; ORF103631; ORF103655; ORF103657; ORF103659	44.13	10.80
ORF103630	MSTRG.8790.8	QSP_Contig867_0062	3prime_partial	1	1	1	ORF020600; ORF020628; ORF020724; ORF021390; ORF025739; ORF025742; ORF026119; ORF026136; ORF103624; ORF103625; ORF103631; ORF103655; ORF103657; ORF103659	44.13	10.80

Note: "PSM associations" is the sum of PSM counts across peptide records linked to each incomplete ORF. Because the same peptide can match more than one incomplete ORF, these ORF-level counts are descriptive association counts and should not be summed across ORFs to obtain a unique experiment-wide PSM total.

Table S4B. Unique-peptide summary of PSM support and precursor mass error.

Peptide sequence	Incomplete ORF(s)	PSMs (n)	Best −10LgP	Median ppm	Max |ppm|	Replicates (n)	PTM	Complete ORF match(es)
AAAVGATVVGAATAATSSIPKEEVKTE	ORF083445; ORF083446	1	46.04	-2.80	2.80	1	None	ORF001845
AADGKGVIVV	ORF091776; ORF091777	2	28.21	-1.95	2.80	2	None	ORF021747; ORF022702
AADGKGVIVVLK	ORF091776; ORF091777	1	25.70	-2.20	2.20	1	None	ORF021747; ORF022702
AAGGTAPKPNVITK	ORF053999	2	47.20	3.00	4.40	1	None	ORF012810; ORF054010; ORF054012
AAGKSSDSVDNAAIK	ORF062221; ORF062223; ORF062226; ORF062227; ORF062229; ORF062232; ORF062233; ORF062236	1	47.15	0.10	0.10	1	None	ORF014920
AAGPVSATTVEKVQEVAV	ORF076584	2	38.86	-5.00	7.50	2	None	ORF018429; ORF076582
AAGPVSATTVEKVQEVAVK	ORF076584	4	47.15	-3.90	11.50	3	None	ORF018429; ORF076582
AAGPVSATTVEKVQEVAVKVA	ORF076584	1	29.22	-6.10	6.10	1	None	ORF018429; ORF076582
AAKESMSDEDLIAAMSSEFVS	ORF083445; ORF083446	1	37.66	-1.20	1.20	1	None	ORF001845
AAPETIAETTLEISTVK	ORF100075; ORF100078; ORF100079	2	45.19	1.35	3.30	2	None	ORF025240
AAPTAPSQSQSAAVSDEALDA	ORF083445; ORF083446	2	47.25	2.40	2.40	1	None	ORF001845
AAPTAPSQSQSAAVSDEALDALM	ORF083445; ORF083446	1	30.52	-7.70	7.70	1	None	ORF001845
AARLPASSGHKPGSANVQILN	ORF053999	1	32.18	-7.60	7.60	1	None	ORF012810; ORF054001; ORF054006; ORF054010; ORF054012; ORF054013; ORF054018
AATAATSSIPKEEVK	ORF083445; ORF083446	1	46.46	-0.40	0.40	1	None	ORF001845
AATAATSSIPKEEVKTE	ORF083445; ORF083446	9	47.42	-0.70	6.30	5	None	ORF001845
AATAATSSIPKEEVKTEK	ORF083445; ORF083446	8	47.15	2.50	5.60	5	None	ORF001845
AATAATSSIPKEEVKTEKK	ORF083445; ORF083446	4	47.14	4.80	11.40	3	None	ORF001845
AATAATSSIPKEEVKTEKKL	ORF083445; ORF083446	1	33.37	8.20	8.20	1	None	ORF001845
AATAATSSIPKEEVKTEKKLV	ORF083445; ORF083446	2	47.08	2.20	2.90	1	None	ORF001845
AATAATSSIPKEEVKTEKKLVS	ORF083445; ORF083446	1	45.42	-13.40	13.40	1	None	ORF001845
AATAATSSIPKEEVKTEKKLVSVD	ORF083445; ORF083446	2	43.92	5.95	9.80	2	None	ORF001845
AATAATSSIPKEEVKTEKKLVSVDKIA	ORF083445; ORF083446	4	47.08	1.85	3.50	3	None	ORF001845
AATSSIPKEEVKTEK	ORF083445; ORF083446	5	46.58	1.80	5.70	5	None	ORF001845
ADFLEGHS	ORF060338; ORF060364; ORF060373	1	27.67	-5.10	5.10	1	None	ORF002404; ORF060337; ORF060344; ORF060345; ORF060349; ORF060350; ORF060353; ORF060354; ORF060356
ADFLEGHSEQATQHVSG	ORF060338; ORF060364	3	45.94	-1.20	9.10	2	None	ORF002404; ORF060337; ORF060344; ORF060345; ORF060349; ORF060350; ORF060353; ORF060354; ORF060356
ADGKGVIVV	ORF091776; ORF091777	2	37.50	0.80	0.90	2	None	ORF021747; ORF022702

Peptide sequence	Incomplete ORF(s)	PSMs (n)	Best −10LgP	Median ppm	Max ∣ppm∣	Replicates (n)	PTM	Complete ORF match(es)
ADRTEIETIVTDPR	ORF062221; ORF062223; ORF062226; ORF062227; ORF062229; ORF062232; ORF062233; ORF062236	14	47.01	4.40	15.80	6	None	ORF014920
ADRTEIETIVTDPRYL	ORF062221; ORF062223; ORF062226; ORF062227; ORF062229; ORF062232; ORF062233; ORF062236	2	37.21	-1.30	3.40	2	None	ORF014920
ADTVDALAGTLGTR	ORF083443	2	43.53	1.00	8.40	2	None	ORF001845
AEILELAGNAAR	ORF067172	2	40.54	-8.85	11.70	2	None	ORF008470; ORF016539; ORF020361; ORF020365; ORF020604; ORF020626; ORF020632; ORF020636; ORF020638; ORF020641; ORF020717; ORF020720; ORF020908; ORF020913; ORF020951; ORF020954; ORF021008; ORF021012; ORF021015; ORF021020; ORF021411; ORF021497; ORF021500; ORF021528; ORF021529; ORF021532; ORF021534; ORF021542; ORF021563; ORF021731; ORF041549; ORF085760
AEILELAGNAARDN	ORF067172	1	46.89	0.50	0.50	1	None	ORF008470; ORF016539; ORF020361; ORF020365; ORF020604; ORF020626; ORF020632; ORF020636; ORF020638; ORF020641; ORF020717; ORF020720; ORF020908; ORF020913; ORF020951; ORF020954; ORF021008; ORF021012; ORF021015; ORF021020; ORF021411; ORF021497; ORF021500; ORF021528; ORF021529; ORF021532; ORF021534; ORF021542; ORF021563; ORF021731; ORF041549; ORF085760
AEILELAGNAARDNK	ORF067172	12	47.44	1.10	3.40	6	None	ORF008470; ORF016539; ORF020361; ORF020365; ORF020604; ORF020626; ORF020632; ORF020636; ORF020638; ORF020641; ORF020717; ORF020720; ORF020908; ORF020913; ORF020951; ORF020954; ORF021008; ORF021012; ORF021015; ORF021020; ORF021411; ORF021497; ORF021500; ORF021528; ORF021529; ORF021532; ORF021534; ORF021542; ORF021563; ORF021731; ORF041549; ORF085760
AEILELAGNAARDNKK	ORF067172	1	45.94	-3.30	3.30	1	None	ORF008470; ORF016539; ORF020361; ORF020365; ORF020604; ORF020632; ORF020636; ORF020638; ORF020641; ORF020717; ORF020720; ORF020908; ORF020913; ORF020951; ORF020954; ORF021008; ORF021012; ORF021015; ORF021020; ORF021411; ORF021497; ORF021500; ORF021528; ORF021529; ORF021532; ORF021534; ORF021542; ORF021563; ORF021731; ORF041549; ORF085760
AEILELAGNAARDNKKT	ORF067172	4	47.09	0.10	3.30	2	None	ORF016539; ORF020361; ORF020365; ORF020604; ORF020632; ORF020636; ORF020638; ORF020641; ORF020717; ORF020720; ORF020908; ORF020913; ORF020951; ORF020954; ORF021008; ORF021012; ORF021015; ORF021020; ORF021497; ORF021500; ORF021528; ORF021529; ORF021532; ORF021534; ORF021542; ORF021563; ORF021731; ORF085760
AEPEAVISASSSSSVQ	ORF083443	6	46.23	12.00	16.90	4	None	ORF001845
AEPPASKQEEPAAATPAAPAS	ORF057182	3	46.69	-3.90	4.60	3	None	ORF012631; ORF057183
AEPPASKQEEPAAATPAAPAS SEASKVSEPTT	ORF057182	2	46.70	-9.50	15.60	2	None	ORF012631; ORF057183
AEPPASKQEEPAAATPAAPAS SEASKVSEPTTEAKVSQPSE	ORF057182	5	47.23	-1.50	6.90	4	None	ORF012631; ORF057183
AESNFGKENPADVIQEQK	ORF076584	1	40.27	5.60	5.60	1	None	ORF018429; ORF076582
AESPVITIQPDSTPL	ORF085019	1	41.85	1.40	1.40	1	None	ORF001870; ORF085013
AGELTEDEVERVVT	ORF071696	1	47.17	1.00	1.00	1	None	ORF018184
AGELTEDEVERVVTI	ORF071696	1	40.34	-5.50	5.50	1	None	ORF018184
AGELTEDEVERVVTIM	ORF071696	4	43.40	-2.05	2.40	2	Oxidation (M)	ORF018184
AGELTEDEVERVVTIM(+15.99)	ORF071696	4	43.40	-2.05	2.40	2	Oxidation (M)	ORF018184
AGELTEDEVERVVTIM(+15.99) QNPR	ORF071696	27	47.46	-0.50	4.00	6	Oxidation (M)	ORF018184

Peptide sequence	Incomplete ORF(s)	PSMs (n)	Best −10LgP	Median ppm	Max |ppm|	Replicates (n)	PTM	Complete ORF match(es)
AGELTEDEVERVVTIM(+15.99) QNPRQ	ORF071696	16	47.41	2.20	6.00	6	Oxidation (M)	ORF018184
AGELTEDEVERVVTIMQNPR	ORF071696	27	47.46	-0.50	4.00	6	Oxidation (M)	ORF018184
AGELTEDEVERVVTIMQNPR Q	ORF071696	16	47.41	2.20	6.00	6	Oxidation (M)	ORF018184
AGGTAPKPNVITK	ORF053999	1	45.85	-3.40	3.40	1	None	ORF012810; ORF054010; ORF054012
AGLQFPVGR	ORF067172	3	30.35	-3.00	4.60	3	None	ORF003297; ORF004026; ORF005986; ORF005988; ORF008392; ORF016539; ORF020361; ORF020365; ORF020604; ORF020632; ORF020636; ORF020638; ORF020641; ORF020717; ORF020720; ORF020908; ORF020913; ORF020951; ORF020954; ORF021008; ORF021012; ORF021015; ORF021020; ORF021497; ORF021500; ORF021528; ORF021529; ORF021532; ORF021534; ORF021537; ORF021542; ORF021563; ORF021731; ORF025126; ORF026134; ORF029575; ORF029578; ORF036876; ORF085760
AGLQFPVGRVHRLL	ORF067172	1	42.10	-6.00	6.00	1	None	ORF016539; ORF020361; ORF020365; ORF020604; ORF020632; ORF020636; ORF020638; ORF020641; ORF020717; ORF020720; ORF020908; ORF020913; ORF020951; ORF020954; ORF021008; ORF021012; ORF021015; ORF021020; ORF021497; ORF021500; ORF021528; ORF021529; ORF021532; ORF021534; ORF021542; ORF021563; ORF021731; ORF025126; ORF026134; ORF085760
AGNDITAPPNKELPASPEK	ORF053999	2	45.39	-3.55	4.40	2	None	ORF012810; ORF054010; ORF054012
AGPGGGGGGAGANILG	ORF089382	1	36.26	2.70	2.70	1	None	ORF021680; ORF089369; ORF089372; ORF089373; ORF089377; ORF089379; ORF089383
AGPVSATTVEKVQEVAVK	ORF076584	1	32.41	-2.50	2.50	1	None	ORF018429; ORF076582
AGSGQAGGAGKFIK	ORF089382	2	46.90	5.25	6.50	2	None	ORF021680; ORF089369; ORF089372; ORF089373; ORF089377; ORF089379; ORF089383
AGSGQGGGGGGGVNVGNLI GGLS	ORF089382	1	45.06	-1.30	1.30	1	None	ORF021680; ORF089369; ORF089372; ORF089373; ORF089377; ORF089379; ORF089383
AGSGQGGGGGGGVNVGNLI GGLSS	ORF089382	4	47.46	0.20	7.10	3	None	ORF021680; ORF089369; ORF089372; ORF089373; ORF089377; ORF089379; ORF089383
AGSRIEEGVPQH	ORF062221; ORF062223; ORF062226; ORF062227; ORF062229; ORF062232; ORF062233; ORF062236	2	34.42	5.30	5.60	2	None	ORF014920
AGSRIEEGVPQHL	ORF062221; ORF062223; ORF062226; ORF062227; ORF062229; ORF062232; ORF062233; ORF062236	2	45.49	-10.30	16.20	1	None	ORF014920
AGSRIEEGVPQHLV	ORF062221; ORF062223; ORF062226; ORF062227; ORF062229; ORF062232; ORF062233; ORF062236	7	44.95	-1.20	6.10	6	None	ORF014920
AGSRIEEGVPQHLVL	ORF062221; ORF062223; ORF062226; ORF062227; ORF062229; ORF062232; ORF062233; ORF062236	2	41.79	-2.15	2.40	2	None	ORF014920
AGSRIEEGVPQHLVLLTGGKS VDDVRGAA	ORF062221; ORF062223; ORF062226; ORF062227; ORF062229; ORF062232; ORF062233; ORF062236	1	33.15	5.90	5.90	1	None	ORF014920
AGTEDSIHIR	ORF095859	1	26.34	4.90	4.90	1	None	ORF023722
AGTTKPTDKKTPESAEKKQ	ORF083445	1	27.48	-3.10	3.10	1	None	ORF001845
AHPLPKTTEKF	ORF032526	3	44.64	0.30	2.50	2	None	ORF004880; ORF005253

Peptide sequence	Incomplete ORF(s)	PSMs (n)	Best −10LgP	Median ppm	Max |ppm|	Replicates (n)	PTM	Complete ORF match(es)
AHSTKDEVQSAVR	ORF062221; ORF062223; ORF062226; ORF062227; ORF062229; ORF062232; ORF062233; ORF062236	2	47.07	1.25	3.60	1	None	ORF014920
AIDTLSGGGGGGQAGGAGK	ORF089382	10	47.46	2.30	5.80	6	None	ORF021680; ORF089369; ORF089372; ORF089373; ORF089377; ORF089379; ORF089383
AIDTLSGGGGGGQAGGAGKL	ORF089382	1	46.63	-3.70	3.70	1	None	ORF021680; ORF089369; ORF089372; ORF089373; ORF089377; ORF089379; ORF089383
AIPEIYRTDTQVVQLD	ORF061770	2	25.94	-14.80	15.00	2	None	ORF015690; ORF061765; ORF061767
AKSVSDGQSAALALASKGVKV	ORF062221; ORF062223; ORF062226; ORF062227; ORF062229; ORF062232; ORF062233; ORF062236	2	47.15	3.05	5.20	1	None	ORF014920
ANGLDNKLREDLERLK	ORF071696	2	38.47	-1.20	3.10	1	None	ORF018184
APAPGQPTDPKVA	ORF100266; ORF100267; ORF100268	1	38.19	-8.60	8.60	1	None	ORF024560
APAPGQPTDPKVAG	ORF100266; ORF100267; ORF100268	2	42.92	4.40	11.00	2	None	ORF024560
APDETNVVR	ORF087621; ORF087623	3	42.35	0.20	11.30	3	None	ORF021436
APGPQYPGAPAPGQPTDPK	ORF100266; ORF100267; ORF100268	3	43.89	0.10	9.60	2	None	ORF024560
APGPQYPGAPAPGQPTDPKVAG	ORF100266; ORF100267; ORF100268	7	47.43	-2.30	4.40	6	None	ORF024560
APGQPTDPKVAG	ORF100266; ORF100267; ORF100268	3	45.56	1.90	1.90	3	None	ORF024560
APPEEKKPKLE	ORF083445; ORF083446	6	46.87	2.40	4.60	6	None	ORF001845
APPRDVVIDVEDTKPL	ORF087579	4	41.13	-2.40	8.10	3	None	ORF021453
APPRDVVIDVEDTKPLL	ORF087579	5	31.85	-3.40	4.00	2	None	ORF021453
APSAAASAGSSKVPTSQ	ORF083445	2	47.46	-6.30	14.70	2	None	ORF001845
AQPEVIVVPLIQVK	ORF086801	2	26.09	-7.70	8.90	2	None	ORF020966; ORF086789; ORF086800
AQVDEVVDIMRVNVDKVLE	ORF044627	1	33.07	-8.50	8.50	1	None	ORF009505
ARIDEILEPAA	ORF101777; ORF101779; ORF101782; ORF101784	3	28.10	-5.30	5.70	3	None	ORF025356
ARVPNAPDETNVVR	ORF087621; ORF087623	7	47.40	7.80	17.90	6	None	ORF021436
ASAPSAAASAGSSKVPTSQ	ORF083445	4	47.46	-3.70	13.90	4	None	ORF001845
ASAPSAAASAGSSKVPTSQE	ORF083445	3	47.26	-6.80	10.10	3	None	ORF001845
ASAPSAAASAGSSKVPTSQER	ORF083445	2	46.66	-4.50	10.80	2	None	ORF001845
ASAPSAAASAGSSKVPTSQERT	ORF083445	2	46.11	-2.45	3.30	2	None	ORF001845
ASNSVDQVFVNVQK	ORF069950	5	46.63	-3.90	10.30	5	None	ORF016799; ORF069954; ORF069958; ORF069960
ASNSVDQVFVNVQKIFDREG	ORF069950	6	43.66	-3.05	3.80	6	None	ORF016799; ORF069954; ORF069958; ORF069960
ASPVAPKLG	ORF053999	1	26.78	-2.60	2.60	1	None	ORF012810; ORF054010; ORF054012
ASSIVPFLIGKNVDSEEANK	ORF062221; ORF062223; ORF062226; ORF062227; ORF062229; ORF062232; ORF062233; ORF062236	1	34.28	-1.30	1.30	1	None	ORF014920
ASSIVPFLIGKNVDSEEANKLT	ORF062221; ORF062223; ORF062226; ORF062227; ORF062229; ORF062232; ORF062233; ORF062236	5	45.98	-1.30	2.80	4	None	ORF014920

Peptide sequence	Incomplete ORF(s)	PSMs (n)	Best −10LgP	Median ppm	Max |ppm|	Replicates (n)	PTM	Complete ORF match(es)
ASVKEVFQGIEEHASGL	ORF103626	1	44.36	-7.20	7.20	1	None	ORF025742; ORF103624; ORF103625
ATSGADRTEIETIVTDPR	ORF062221; ORF062223; ORF062226; ORF062227; ORF062229; ORF062232; ORF062233; ORF062236	6	47.32	-1.15	3.90	5	None	ORF014920
ATSSIPKEEVKTEK	ORF083445; ORF083446	1	46.82	-2.00	2.00	1	None	ORF001845
ATVTPSPAKA	ORF027326	4	39.22	1.50	8.40	4	None	ORF005745
ATVVGAATAATSSIPKEEVKTEK	ORF083445; ORF083446	1	43.89	-2.50	2.50	1	None	ORF001845
AVATSGADRTEIETIVTDPR	ORF062221; ORF062223; ORF062226; ORF062227; ORF062229; ORF062232; ORF062233; ORF062236	6	47.44	2.05	5.60	6	None	ORF014920
AVATSGADRTEIETIVTDPRYL	ORF062221; ORF062223; ORF062226; ORF062227; ORF062229; ORF062232; ORF062233; ORF062236	1	42.02	0.10	0.10	1	None	ORF014920
AVLLPKKT	ORF067172	2	37.35	1.05	3.70	2	None	ORF004026; ORF005986; ORF016539; ORF020361; ORF020365; ORF020604; ORF020626; ORF020632; ORF020636; ORF020638; ORF020641; ORF020717; ORF020720; ORF020908; ORF020913; ORF020951; ORF020954; ORF021008; ORF021012; ORF021015; ORF021020; ORF021386; ORF021497; ORF021500; ORF021528; ORF021529; ORF021532; ORF021534; ORF021537; ORF021542; ORF021563; ORF026134; ORF085760
DALSSEEEHVVPAPIIPAETIG	ORF100296; ORF100297; ORF100298; ORF100299; ORF100300	1	25.16	-5.70	5.70	1	None	ORF024865; ORF100294; ORF100295
DALSSEEEHVVPAPIIPAETIGSVR	ORF100296; ORF100297; ORF100298; ORF100299; ORF100300	1	46.80	-5.20	5.20	1	None	ORF024865; ORF100294; ORF100295
DASKGDDWLPAGTEDSIHIR	ORF095859	3	47.14	-1.10	4.40	3	None	ORF023722
DASNSVDQVFVNVQ	ORF069950	2	32.46	0.20	1.50	2	None	ORF016799; ORF069954; ORF069958; ORF069960
DASNSVDQVFVNVQK	ORF069950	2	40.97	1.20	1.90	2	None	ORF016799; ORF069954; ORF069958; ORF069960
DDEAMSVVEMLKDK	ORF100296; ORF100297; ORF100298; ORF100299; ORF100300	1	46.95	-0.40	0.40	1	None	ORF024865; ORF100294; ORF100295
DDEVQVVR	ORF040982	6	44.41	-1.10	2.10	5	None	ORF008943; ORF022249
DDIEILR	ORF062221; ORF062223; ORF062226; ORF062227; ORF062229; ORF062232; ORF062233; ORF062236	6	27.67	-3.20	3.50	2	None	ORF014920
DDRLEQYTHAIET	ORF079438; ORF079444	4	47.42	2.60	6.40	2	None	ORF020265; ORF079440
DDRLEQYTHAIETS	ORF079438; ORF079444	2	44.96	-5.05	6.90	2	None	ORF020265; ORF079440
DDRTTQEELNDLFQK	ORF080398	1	41.33	-1.20	1.20	1	None	ORF019505
DDTNRSIIR	ORF041048	1	26.10	-0.60	0.60	1	None	ORF003580
DDWLPAGTEDSIHIR	ORF095859	35	47.45	-1.20	4.20	6	None	ORF023722
DDWLPAGTEDSIHIRIQ	ORF095859	3	32.88	-0.50	1.70	3	None	ORF023722
DDWLPAGTEDSIHIRIQQ	ORF095859	1	45.73	-2.70	2.70	1	None	ORF023722
DEEYGRESGPPA	ORF027326	3	46.62	3.80	5.80	3	None	ORF005745
DEEYGRESGPPAK	ORF027326	4	45.58	3.05	11.90	2	None	ORF005745
DEPLTQNDIR	ORF078612	3	38.02	5.80	9.20	3	None	ORF019339; ORF078617; ORF078626; ORF078629; ORF078634

Peptide sequence	Incomplete ORF(s)	PSMs (n)	Best −10LgP	Median ppm	Max ∣ppm∣	Replicates (n)	PTM	Complete ORF match(es)
DEPLTQNDIRNTKDVDM(+15.99)K	ORF078612	7	47.43	3.50	5.20	4	Oxidation (M)	ORF019339; ORF078617; ORF078626; ORF078629; ORF078634
DEPLTQNDIRNTKDVDM(+15.99)KIGV	ORF078612	16	47.39	1.85	6.10	5	Oxidation (M)	ORF019339; ORF078617; ORF078626; ORF078629; ORF078634
DEPLTQNDIRNTKDVDMK	ORF078612	7	47.43	3.50	5.20	4	Oxidation (M)	ORF019339; ORF078617; ORF078626; ORF078629; ORF078634
DEPLTQNDIRNTKDVDMKIGV	ORF078612	16	47.39	1.85	6.10	5	Oxidation (M)	ORF019339; ORF078617; ORF078626; ORF078629; ORF078634
DESLLTSVTTLDQDKIK	ORF062221; ORF062223; ORF062226; ORF062227; ORF062229; ORF062232; ORF062233; ORF062236	4	45.63	-4.50	5.40	4	None	ORF014920
DESLRNHFEQWGALT	ORF031536; ORF031542	4	33.06	-0.50	4.90	3	None	ORF004221
DEVERVVTIMQNPR	ORF071696	4	47.29	-1.50	6.90	3	None	ORF018184
DFGEEDLFHQQGDPRTK	ORF038836	1	44.62	3.20	3.20	1	None	ORF001160; ORF038831; ORF038834
DFLEGHSEQATQHVSG	ORF060338; ORF060364	2	46.30	0.95	3.80	1	None	ORF002404; ORF060337; ORF060344; ORF060345; ORF060349; ORF060350; ORF060353; ORF060354; ORF060356
DFLEGHSEQATQHVSGLQ	ORF060338; ORF060364	1	45.79	3.90	3.90	1	None	ORF002404; ORF060337; ORF060344; ORF060345; ORF060349; ORF060350; ORF060353; ORF060354; ORF060356
DGKQRDPSMHGLILNDIFK	ORF062015	1	46.58	-5.10	5.10	1	None	ORF015592
DGKYSQVLANGLDNK	ORF071696	2	30.95	0.40	1.30	1	None	ORF018184
DGRGAAQNIIPASTGAA	ORF088248; ORF088249	3	32.47	1.00	2.90	3	None	ORF021197
DGRKIIVPQGPPKPTPK	ORF066708	1	46.05	-1.30	1.30	1	None	ORF016200; ORF066693; ORF066716
DGSTRTGSDGLVNIR	ORF062221; ORF062223; ORF062226; ORF062227; ORF062229; ORF062232; ORF062233; ORF062236	4	47.14	-4.00	5.90	4	None	ORF014920
DGYGNNPPYSGNR	ORF031536; ORF031542	4	47.26	5.85	9.80	4	None	ORF004221
DHNNRDIIMDAVTKAE	ORF062221; ORF062223; ORF062226; ORF062227; ORF062229; ORF062232; ORF062233; ORF062236	1	46.76	-0.40	0.40	1	None	ORF014920
DHNNRDIIMDAVTKAEFK	ORF062221; ORF062223; ORF062226; ORF062227; ORF062229; ORF062232; ORF062233; ORF062236	1	31.92	-8.10	8.10	1	None	ORF014920
DHYAIIKFPLTTESAM(+15.99)	ORF067090	2	42.45	-5.30	5.30	1	Oxidation (M)	ORF006342; ORF016564
DIRNTKDVDMKIGV	ORF078612	1	46.40	2.80	2.80	1	None	ORF019339; ORF078617; ORF078626; ORF078629; ORF078634
DISQLGNVYQTITTR	ORF062221; ORF062223; ORF062226; ORF062227; ORF062229; ORF062232; ORF062233; ORF062236	2	28.80	2.25	2.80	2	None	ORF014920
DLIAYIATKE	ORF088248; ORF088249	1	27.15	-0.80	0.80	1	None	ORF021197
DLLHPSPEEEK	ORF102745; ORF102746	1	36.26	0.90	0.90	1	None	ORF024572
DLLHPSPEEEKR	ORF102745; ORF102746	3	47.19	-1.10	3.20	3	None	ORF024572
DNIDEGARQIK	ORF062221; ORF062223; ORF062226; ORF062227; ORF062229; ORF062232; ORF062233; ORF062236	7	47.16	3.50	3.90	6	None	ORF014920

Peptide sequence	Incomplete ORF(s)	PSMs (n)	Best −10LgP	Median ppm	Max ∣ppm∣	Replicates (n)	PTM	Complete ORF match(es)
DNKLREDLERLK	ORF071696	2	45.75	-6.05	7.10	2	None	ORF018184
DNNTLVFIVDVK	ORF067090	3	45.58	4.00	8.60	3	None	ORF006342; ORF016564
DNTRVAVIQFSEDPKVEF	ORF062221; ORF062223; ORF062226; ORF062227; ORF062229; ORF062232; ORF062233; ORF062236	2	46.45	-4.30	4.30	1	None	ORF014920
DPFADASKG	ORF095859	5	43.05	0.60	5.40	5	None	ORF023722
DQDEPLTQNDIR	ORF078612	3	46.28	8.70	19.10	3	None	ORF019339; ORF078617; ORF078626; ORF078629; ORF078634
DQDEPLTQNDIRNTKDVDMKIGV	ORF078612	2	40.21	-0.75	4.90	1	None	ORF019339; ORF078617; ORF078626; ORF078629; ORF078634
DQKLSELDDRADALQAGASQFETSAA	ORF044627	1	40.61	0.00	0.00	1	None	ORF009505
DQLYHWTGSD	ORF090968	4	46.09	3.10	4.80	3	None	ORF021892
DQTNQGGNFVVTAAGK	ORF044453	2	47.44	6.35	10.30	2	None	ORF008679
DRTEIETIVTDPR	ORF062221; ORF062223; ORF062226; ORF062227; ORF062229; ORF062232; ORF062233; ORF062236	14	46.04	0.15	2.80	6	None	ORF014920
DRTEIETIVTDPRY	ORF062221; ORF062223; ORF062226; ORF062227; ORF062229; ORF062232; ORF062233; ORF062236	2	40.50	-2.85	3.90	2	None	ORF014920
DRTEIETIVTDPRYL	ORF062221; ORF062223; ORF062226; ORF062227; ORF062229; ORF062232; ORF062233; ORF062236	4	40.66	-0.70	6.30	3	None	ORF014920
DTAALIQTK	ORF044453	9	42.94	0.80	3.20	6	None	ORF008679; ORF008680
DTLSGGGGGGQAGGAGK	ORF089382	4	47.46	-3.20	16.30	4	None	ORF021680; ORF089369; ORF089372; ORF089373; ORF089377; ORF089379; ORF089383
DVANKIGII	ORF067090	3	43.65	-0.40	2.80	3	None	ORF006342; ORF016564
DVKPADTKKPNLT	ORF053999	1	46.89	7.20	7.20	1	None	ORF012810; ORF054010; ORF054012
DVLTLLESEREA	ORF041048	4	32.93	4.10	8.20	4	None	ORF003580
DVLTLLESEREARR	ORF041048	3	36.76	1.40	2.40	3	None	ORF003580
DVVIDVEDTKPL	ORF087579	4	37.20	2.30	4.00	4	None	ORF021453
DWFLNRQKDIKD	ORF071696	1	38.23	0.50	0.50	1	None	ORF018184
DWLPAGTEDSIHIR	ORF095859	6	46.77	-1.10	5.90	5	None	ORF023722
DYVVESTGVFTTIDK	ORF088248; ORF088249	2	36.63	4.15	5.80	2	None	ORF021197
DYYESYSAARVPPPPPIA	ORF069609	1	30.57	-4.90	4.90	1	None	ORF001221; ORF069605; ORF069606; ORF069607; ORF069608
EAAAKYQPDPPPPR	ORF073021	1	28.56	7.60	7.60	1	None	ORF017885
EAPSTEGAESEITGVVVEK	ORF100055; ORF100075; ORF100076	1	44.53	-4.80	4.80	1	None	ORF025240
EAPSTEGAESEITGVVVEKV	ORF100055; ORF100075; ORF100076	2	39.18	-4.00	4.00	1	None	ORF025240
EDEVERVVTIMQNPR	ORF071696	2	30.41	-0.55	1.90	2	None	ORF018184
EDEVERVVTIMQNPRQ	ORF071696	2	45.43	-0.30	3.80	2	None	ORF018184
EDLERLKKIK	ORF071696	1	31.69	3.20	3.20	1	None	ORF018184
EDLVPIKPAN	ORF088463	1	27.60	-4.30	4.30	1	None	ORF021312

Peptide sequence	Incomplete ORF(s)	PSMs (n)	Best −10LgP	Median ppm	Max |ppm|	Replicates (n)	PTM	Complete ORF match(es)
EDLVPIKPANK	ORF088463	1	27.10	8.30	8.30	1	None	ORF021312
EDNNTLVFIVDVK	ORF067090	8	47.20	1.20	4.50	5	None	ORF006342; ORF016564
EEDLFHQQGDPRTK	ORF038836	3	46.64	5.40	14.30	3	None	ORF001160; ORF038831; ORF038834
EEEDVLSGWKLPT	ORF100055; ORF100073	1	32.70	-1.10	1.10	1	None	ORF025240; ORF100065
EEEHVVPAPIIPAETIGSVR	ORF100296; ORF100297; ORF100298; ORF100299; ORF100300	5	45.32	-2.30	7.30	5	None	ORF024865; ORF100294; ORF100295
EEEHVVPAPIIPAETIGSVRER	ORF100296; ORF100297; ORF100298; ORF100299; ORF100300	1	37.15	-12.00	12.00	1	None	ORF024865; ORF100294; ORF100295
EEGVPQHL	ORF062221; ORF062223; ORF062226; ORF062227; ORF062229; ORF062232; ORF062233; ORF062236	5	35.80	2.90	5.50	4	None	ORF014920
EEGVPQHLV	ORF062221; ORF062223; ORF062226; ORF062227; ORF062229; ORF062232; ORF062233; ORF062236	1	41.13	-2.40	2.40	1	None	ORF014920
EEGVPQHLVL	ORF062221; ORF062223; ORF062226; ORF062227; ORF062229; ORF062232; ORF062233; ORF062236	1	26.62	-1.30	1.30	1	None	ORF014920
EEIKDFLLT	ORF098458	4	38.46	-0.60	0.60	2	None	ORF023230
EEIKDFLLTAR	ORF098458	6	40.78	-0.45	2.70	3	None	ORF023230
EEKEDLVPIKPAN	ORF088463	1	46.27	-1.90	1.90	1	None	ORF021312
EEKEDLVPIKPANK	ORF088463	4	43.93	1.85	3.40	2	None	ORF021312
EEVDASMNARPH	ORF031536; ORF031542	4	46.56	2.55	8.30	4	None	ORF004221
EEVDASMNARPHRVDGRM(+15.99)VEPK	ORF031536; ORF031542	4	43.85	8.05	18.30	4	Oxidation (M)	ORF004221
EEVDASMNARPHRVDGRMVEPK	ORF031536; ORF031542	4	43.85	8.05	18.30	4	Oxidation (M)	ORF004221
EFKELPSIESI	ORF062221; ORF062223; ORF062226; ORF062227; ORF062229; ORF062232; ORF062233; ORF062236	2	28.18	-0.90	0.90	1	None	ORF014920
EFKELPSIESIL	ORF062221; ORF062223; ORF062226; ORF062227; ORF062229; ORF062232; ORF062233; ORF062236	3	37.58	2.10	3.60	3	None	ORF014920
EFKELPSIESILFK	ORF062221; ORF062223; ORF062226; ORF062227; ORF062229; ORF062232; ORF062233; ORF062236	4	44.78	3.00	6.90	3	None	ORF014920
EFLRQADFLEGHS	ORF060338; ORF060364; ORF060373	1	33.01	-1.60	1.60	1	None	ORF002404; ORF060337; ORF060344; ORF060345; ORF060349; ORF060350; ORF060353; ORF060354; ORF060356
EFMDDTNRSII	ORF041048	1	37.63	-1.50	1.50	1	None	ORF003580
EFMDDTNRSIIR	ORF041048	5	39.01	-2.90	5.40	5	None	ORF003580
EGDVLTLLESER	ORF041048	8	38.97	1.70	3.10	5	None	ORF003580
EGDVLTLLESEREA	ORF041048	6	46.98	0.95	6.60	5	None	ORF003580
EGDVLTLLESEREAR	ORF041048	3	32.89	-2.00	3.20	3	None	ORF003580

Peptide sequence	Incomplete ORF(s)	PSMs (n)	Best −10LgP	Median ppm	Max ∣ppm∣	Replicates (n)	PTM	Complete ORF match(es)
EGDVLTLLESEREARR	ORF041048	7	43.76	0.00	3.50	5	None	ORF003580
EGHSEQATQHVSGLQ	ORF060338; ORF060364	6	46.33	1.75	4.90	5	None	ORF002404; ORF060337; ORF060344; ORF060345; ORF060349; ORF060350; ORF060353; ORF060354; ORF060356
EGLDVDKDKIR	ORF062221; ORF062223; ORF062226; ORF062227; ORF062229; ORF062232; ORF062233; ORF062236	1	41.73	-0.60	0.60	1	None	ORF014920
EGLWEIENNPTVK	ORF101777; ORF101779; ORF101782; ORF101784	15	46.38	1.10	1.70	6	None	ORF025356
EGLWEIENNPTVKA	ORF101777; ORF101779; ORF101782; ORF101784	2	39.00	8.15	12.40	2	None	ORF025356
EHPDYAFSYYLSNIAKL	ORF033209	1	43.87	-2.50	2.50	1	None	ORF001812
EIENNPTVK	ORF101777; ORF101779; ORF101782; ORF101784	8	42.61	0.70	7.90	6	None	ORF025356
EIETIVTDPR	ORF062221; ORF062223; ORF062226; ORF062227; ORF062229; ORF062232; ORF062233; ORF062236	4	44.24	-1.00	2.10	3	None	ORF014920
EILELAGNAARDNK	ORF067172	5	47.27	0.30	3.00	5	None	ORF008470; ORF016539; ORF020361; ORF020365; ORF020604; ORF020626; ORF020632; ORF020636; ORF020638; ORF020641; ORF020717; ORF020720; ORF020908; ORF020913; ORF020951; ORF020954; ORF021008; ORF021012; ORF021015; ORF021020; ORF021411; ORF021497; ORF021500; ORF021528; ORF021529; ORF021532; ORF021534; ORF021542; ORF021563; ORF021731; ORF041549; ORF085760
EKKGLLDIIH	ORF033209	4	46.26	1.85	4.20	4	None	ORF001812
ELIGRLVETMDVNQDNTRVA	ORF062221; ORF062223; ORF062226; ORF062227; ORF062229; ORF062232; ORF062233; ORF062236	1	40.10	-10.20	10.20	1	None	ORF014920
ELKTDDIEILR	ORF062221; ORF062223; ORF062226; ORF062227; ORF062229; ORF062232; ORF062233; ORF062236	5	42.88	-2.00	11.70	3	None	ORF014920
ELTEDEVERVVTIMQNPR	ORF071696	2	38.46	0.85	7.60	2	None	ORF018184
ELTEDEVERVVTIMQNPRQ	ORF071696	4	46.75	-4.90	6.70	2	None	ORF018184
ELWKDVDRI	ORF091780	1	32.98	-5.20	5.20	1	None	ORF017086; ORF022708; ORF068763; ORF098038
EPAADGKGVIVV	ORF091776; ORF091777	1	39.64	0.60	0.60	1	None	ORF021747; ORF022702
EPPLPIH	ORF079438; ORF079444	2	25.39	-0.10	1.60	2	None	ORF020265; ORF079440
EPPLPIHK	ORF079438; ORF079444	4	42.74	-1.25	3.00	4	None	ORF020265; ORF079440
EPPLPIHKIDDRLE	ORF079438; ORF079444	8	47.35	-0.70	2.70	6	None	ORF020265; ORF079440
EPPLPIHKIDDRLEQ	ORF079438; ORF079444	2	38.58	-1.10	6.50	2	None	ORF020265; ORF079440
EQATQHVSGLQ	ORF060338; ORF060364	5	45.52	5.00	6.40	4	None	ORF002404; ORF060337; ORF060344; ORF060345; ORF060349; ORF060350; ORF060353; ORF060354; ORF060356
ETAASKDASEPTSADSSMVSE PKTPKK	ORF085010	1	43.53	4.60	4.60	1	None	ORF001870; ORF085013; ORF085017
ETAYLGPKDIVAYEETKA	ORF101777; ORF101779; ORF101782; ORF101784	8	46.47	-2.10	4.20	6	None	ORF025356

Peptide sequence	Incomplete ORF(s)	PSMs (n)	Best −10LgP	Median ppm	Max ǀppmǀ	Replicates (n)	PTM	Complete ORF match(es)
ETMDVNQDNTRVAVIQ	ORF062221; ORF062223; ORF062226; ORF062227; ORF062229; ORF062232; ORF062233; ORF062236	2	46.81	-5.80	10.20	2	None	ORF014920
ETMDVNQDNTRVAVIQFSEDPKVE	ORF062221; ORF062223; ORF062226; ORF062227; ORF062229; ORF062232; ORF062233; ORF062236	1	47.12	3.10	3.10	1	None	ORF014920
EVFQGIEEHASGL	ORF103626	1	38.74	1.70	1.70	1	None	ORF025742; ORF103624; ORF103625
EVGLAKEDQLKVHG	ORF095859	1	36.41	-0.60	0.60	1	None	ORF023722
EVVDIM(+15.99)RVNVDKVLE	ORF044627	2	43.77	-1.65	3.60	2	Oxidation (M)	ORF009505; ORF022326; ORF092996; ORF093003
EVVDIM(+15.99)RVNVDKVLER	ORF044627	4	41.04	-1.80	4.20	3	Oxidation (M)	ORF009505; ORF022326; ORF092996; ORF093003
EVVDIMRVNVD	ORF044627	3	33.06	1.20	2.60	2	None	ORF009505; ORF011313; ORF022326; ORF092996; ORF093003
EVVDIMRVNVDK	ORF044627	1	35.79	1.30	1.30	1	None	ORF009505; ORF011313; ORF022326; ORF092996; ORF093003
EVVDIMRVNVDKVLER	ORF044627	4	41.04	-1.80	4.20	3	Oxidation (M)	ORF009505; ORF022326; ORF092996; ORF093003
EYGEVIQLQGDQR	ORF095859	7	44.30	4.20	5.50	4	None	ORF013742; ORF023722; ORF058081
EYGRESGPPA	ORF027326	1	32.59	6.00	6.00	1	None	ORF005745
FDKEKKGLLDIIH	ORF033209	5	45.45	-2.30	6.20	5	None	ORF001812
FGSDGYGNNPPYSGNR	ORF031536; ORF031542	1	43.71	-0.20	0.20	1	None	ORF004221
FGTHETAYLGPKDIVA	ORF101777; ORF101779; ORF101782; ORF101784	4	45.43	-1.25	3.00	4	None	ORF025356
FGTHETAYLGPKDIVAYE	ORF101777; ORF101779; ORF101782; ORF101784	3	45.88	-3.50	5.50	3	None	ORF025356
FGTHETAYLGPKDIVAYEETKA	ORF101777; ORF101779; ORF101782; ORF101784	8	45.95	0.00	5.60	4	None	ORF025356
FIGGLSFETTDESLR	ORF031536; ORF031542	3	44.10	1.30	2.20	3	None	ORF004221
FIITGAKSVSDGQSAALA	ORF062221; ORF062223; ORF062226; ORF062227; ORF062229; ORF062232; ORF062233; ORF062236	1	45.01	-2.10	2.10	1	None	ORF014920
FIKGAIDTLSGGGGGGQAGGAG	ORF089382	1	45.17	-8.00	8.00	1	None	ORF021680; ORF089369; ORF089372; ORF089373; ORF089377; ORF089379; ORF089383
FIKGAIDTLSGGGGGGQAGGAGK	ORF089382	5	47.46	0.30	12.60	3	None	ORF021680; ORF089369; ORF089372; ORF089373; ORF089377; ORF089379; ORF089383
FIQRVVEGLDVDKDKIR	ORF062221; ORF062223; ORF062226; ORF062227; ORF062229; ORF062232; ORF062233; ORF062236	7	47.46	0.10	6.00	6	None	ORF014920
FKELPSIESIL	ORF062221; ORF062223; ORF062226; ORF062227; ORF062229; ORF062232; ORF062233; ORF062236	3	27.48	0.10	3.70	3	None	ORF014920
FKNLTDADLIQLLQNYGIK	ORF076580; ORF076584	6	47.42	-5.05	8.50	5	None	ORF018429; ORF076577; ORF076579; ORF076582
FLEGHSEQATQHVS	ORF060338; ORF060364	1	30.53	-0.50	0.50	1	None	ORF002404; ORF060337; ORF060344; ORF060345; ORF060349; ORF060350; ORF060353; ORF060354; ORF060356; ORF060365

Peptide sequence	Incomplete ORF(s)	PSMs (n)	Best −10LgP	Median ppm	Max |ppm|	Replicates (n)	PTM	Complete ORF match(es)
FLEGHSEQATQHVSG	ORF060338; ORF060364	4	43.41	7.20	18.80	3	None	ORF002404; ORF060337; ORF060344; ORF060345; ORF060349; ORF060350; ORF060353; ORF060354; ORF060356; ORF060365
FLEGHSEQATQHVSGLQ	ORF060338; ORF060364	3	47.06	-0.60	2.00	2	None	ORF002404; ORF060337; ORF060344; ORF060345; ORF060349; ORF060350; ORF060353; ORF060354; ORF060356
FSEDPKVE	ORF062221; ORF062223; ORF062226; ORF062227; ORF062229; ORF062232; ORF062233; ORF062236	2	36.84	-0.05	1.60	2	None	ORF014920
FSEGLWEIENNPTVK	ORF101777; ORF101779; ORF101782; ORF101784	6	47.26	2.15	3.50	6	None	ORF025356
FVTYSTTEEVDASMNARPHRVDGRMVEPK	ORF031536; ORF031542	1	42.39	7.60	7.60	1	None	ORF004221
GAAKILGTNGVK	ORF062221; ORF062223; ORF062226; ORF062227; ORF062229; ORF062232; ORF062233; ORF062236	1	47.05	3.40	3.40	1	None	ORF014920
GADRAKKAIDELNGRELH	ORF080398	1	32.34	5.10	5.10	1	None	ORF019505
GADRTEIETIVTDPR	ORF062221; ORF062223; ORF062226; ORF062227; ORF062229; ORF062232; ORF062233; ORF062236	10	46.34	0.55	3.60	6	None	ORF014920
GAGGQGGGGAASNILG	ORF089382	1	46.77	2.00	2.00	1	None	ORF021680; ORF089369; ORF089372; ORF089373; ORF089377; ORF089379; ORF089383
GAGLIEGLVAKELDSPVEPGQDAK	ORF097179; ORF097181	1	31.93	4.70	4.70	1	None	ORF023790
GAIDTLSGGGGGGQAGGAGK	ORF089382	10	47.46	1.60	3.80	6	None	ORF021680; ORF089369; ORF089372; ORF089373; ORF089377; ORF089379; ORF089383
GAIDTLSGGGGGGQAGGAGKL	ORF089382	3	47.46	-1.60	9.40	3	None	ORF021680; ORF089369; ORF089372; ORF089373; ORF089377; ORF089379; ORF089383
GAIDTLSGGGGGGQAGGAGKLF	ORF089382	1	41.28	-2.20	2.20	1	None	ORF021680; ORF089369; ORF089372; ORF089373; ORF089377; ORF089379; ORF089383
GAPAPGQPTDPK	ORF100266; ORF100267; ORF100268	5	44.20	1.60	16.90	4	None	ORF024560
GAPAPGQPTDPKVA	ORF100266; ORF100267; ORF100268	2	40.36	-6.00	7.70	2	None	ORF024560
GAPAPGQPTDPKVAG	ORF100266; ORF100267; ORF100268	4	47.03	-2.70	6.40	3	None	ORF024560
GAPAPGQPTDPKVAGQSGP	ORF100266; ORF100267; ORF100268	1	46.95	-3.40	3.40	1	None	ORF024560
GAPSPITDAQTSRPK	ORF060786; ORF060787	1	31.52	9.60	9.60	1	None	ORF014594; ORF060773; ORF060779; ORF060783
GDDWLPAGTEDSIHIR	ORF095859	4	40.44	0.20	5.80	2	None	ORF023722
GDVLTLLESER	ORF041048	3	32.66	0.60	1.30	2	None	ORF003580
GDVLTLLESEREA	ORF041048	7	46.83	-1.70	5.20	5	None	ORF003580
GDVLTLLESEREAR	ORF041048	2	39.87	-4.00	5.00	2	None	ORF003580
GDVLTLLESEREARR	ORF041048	4	43.53	-1.50	1.90	4	None	ORF003580
GELTEDEVERVVTIM	ORF071696	1	40.79	2.00	2.00	1	None	ORF018184
GELTEDEVERVVTIMQNPR	ORF071696	6	47.42	-9.20	9.60	4	None	ORF018184
GELTEDEVERVVTIMQNPRQ	ORF071696	5	47.18	0.60	1.10	2	None	ORF018184
GEVIQLQGDQR	ORF095859	4	43.35	-3.30	5.50	4	None	ORF013742; ORF023722; ORF058081

Peptide sequence	Incomplete ORF(s)	PSMs (n)	Best −10LgP	Median ppm	Max |ppm|	Replicates (n)	PTM	Complete ORF match(es)
GFGGGDGFDDR	ORF086823	1	41.77	-0.20	0.20	1	None	ORF020980; ORF086821
GFGGGDGFDDRLR	ORF086823	2	36.01	-1.75	3.90	2	None	ORF020980; ORF086821
GFSEGLWEIENNPTVK	ORF101777; ORF101779; ORF101782; ORF101784	12	47.44	1.20	4.20	6	None	ORF025356
GGGDGFDDRLR	ORF086823	2	45.94	5.90	7.10	2	None	ORF020980; ORF086821
GGGGGGFGGRGFG	ORF073021	1	27.71	-1.80	1.80	1	None	ORF017885
GGGGGGGFGGRGFG	ORF073021	2	42.95	3.10	3.90	2	None	ORF017885
GGGGGGGGFGGRGFG	ORF073021	2	46.55	-0.60	6.50	2	None	ORF017885
GGGGGGGGFGGRGFGGGFG GGGGGGM	ORF073021	1	30.06	-1.30	1.30	1	None	ORF017885
GGGGGGGGGFGDER	ORF065585; ORF065586; ORF065587	1	34.10	5.50	5.50	1	None	ORF015511
GGGGGGGGGFGGRGFG	ORF073021	3	47.09	-0.50	7.90	3	None	ORF017885
GGGGGGGGGFGGRGFGGG	ORF073021	1	47.03	5.50	5.50	1	None	ORF017885
GGGGGGGVNVGNLIGGLS	ORF089382	1	44.87	2.50	2.50	1	None	ORF021680; ORF089369; ORF089372; ORF089373; ORF089377; ORF089379; ORF089383
GGGGGGGVNVGNLIGGLSS	ORF089382	1	42.65	-0.10	0.10	1	None	ORF021680; ORF089369; ORF089372; ORF089373; ORF089377; ORF089379; ORF089383
GGKSVDDVRGAA	ORF062221; ORF062223; ORF062226; ORF062227; ORF062229; ORF062232; ORF062233; ORF062236	4	46.69	4.05	6.20	4	None	ORF014920
GGLSFETTDESLR	ORF031536; ORF031542	7	46.79	3.10	4.60	6	None	ORF004221
GGLSSLAGSGQGGGGGGGV NVGN	ORF089382	1	28.86	3.40	3.40	1	None	ORF021680; ORF089369; ORF089372; ORF089373; ORF089377; ORF089379; ORF089383
GGMPPAGGQQYPGGMPPA	ORF100266; ORF100267	1	27.47	-11.50	11.50	1	None	ORF024560
GGNGNFGGGSGGGGGSNFN DFGS	ORF031536; ORF031542	1	43.07	-1.40	1.40	1	None	ORF004221
GGNGNFGGGSGGGGGSNFN DFGSYNN	ORF031536; ORF031542	1	47.15	-3.70	3.70	1	None	ORF004221
GGNGNFGGGSGGGGGSNFN DFGSYNNQS	ORF031536; ORF031542	3	46.71	-2.40	9.60	2	None	ORF004221
GGNGNFGGGSGGGGGSNFN DFGSYNNQSSSNFGPM	ORF031536; ORF031542	1	45.96	2.80	2.80	1	None	ORF004221
GGSGGGGGSNFNDFGSYNN QS	ORF031536; ORF031542	1	37.70	1.50	1.50	1	None	ORF004221
GGSGGGGGSNFNDFGSYNN QSSSNFGP	ORF031536; ORF031542	4	46.53	-3.40	7.80	3	None	ORF004221
GGSGGGGGSNFNDFGSYNN QSSSNFGPM	ORF031536; ORF031542	10	47.26	-4.70	7.00	5	Oxidation (M)	ORF004221
GGSGGGGGSNFNDFGSYNN QSSSNFGPM(+15.99)	ORF031536; ORF031542	10	47.26	-4.70	7.00	5	Oxidation (M)	ORF004221
GGSSANIGNALEYVSK	ORF062221; ORF062223; ORF062226; ORF062227; ORF062229; ORF062232; ORF062233; ORF062236	3	46.21	-5.60	6.40	3	None	ORF014920

Peptide sequence	Incomplete ORF(s)	PSMs (n)	Best −10LgP	Median ppm	Max ∣ppm∣	Replicates (n)	PTM	Complete ORF match(es)
GGSSANIGNALEYVSKNI	ORF062221; ORF062223; ORF062226; ORF062227; ORF062229; ORF062232; ORF062233; ORF062236	1	26.65	-0.90	0.90	1	None	ORF014920
GGTAPKPNVITK	ORF053999	1	46.85	-5.40	5.40	1	None	ORF012810; ORF054010; ORF054012
GGTSLNIGKALD	ORF062221; ORF062223; ORF062226; ORF062227; ORF062229; ORF062232; ORF062233; ORF062236	2	43.45	-9.90	11.70	2	None	ORF014920
GGTSLNIGKALDYVA	ORF062221; ORF062223; ORF062226; ORF062227; ORF062229; ORF062232; ORF062233; ORF062236	4	42.26	-2.00	2.10	3	None	ORF014920
GGTSLNIGKALDYVAKN	ORF062221; ORF062223; ORF062226; ORF062227; ORF062229; ORF062232; ORF062233; ORF062236	1	43.62	-6.60	6.60	1	None	ORF014920
GGTSLNIGKALDYVAKNQ	ORF062221; ORF062223; ORF062226; ORF062227; ORF062229; ORF062232; ORF062233; ORF062236	1	42.60	0.90	0.90	1	None	ORF014920
GGVQEELIRTVDVK	ORF044453	2	38.23	-3.85	5.40	2	None	ORF008679
GGVQEELIRTVDVKNIVPK	ORF044453	2	45.70	1.60	1.60	1	None	ORF008679
GGVTIAQGGVLPNIQ	ORF067172	7	47.11	1.30	3.90	5	None	ORF016539; ORF020361; ORF020365; ORF020604; ORF020632; ORF020636; ORF020638; ORF020641; ORF020717; ORF020720; ORF020908; ORF020913; ORF020951; ORF020954; ORF021008; ORF021012; ORF021015; ORF021020; ORF021386; ORF021497; ORF021500; ORF021528; ORF021529; ORF021532; ORF021534; ORF021542; ORF021563; ORF021731; ORF025126; ORF085760
GGVTIAQGGVLPNIQA	ORF067172	1	45.58	-2.10	2.10	1	None	ORF016539; ORF020361; ORF020365; ORF020604; ORF020632; ORF020636; ORF020638; ORF020641; ORF020717; ORF020720; ORF020908; ORF020913; ORF020951; ORF020954; ORF021008; ORF021012; ORF021015; ORF021020; ORF021386; ORF021497; ORF021500; ORF021528; ORF021529; ORF021532; ORF021534; ORF021542; ORF021563; ORF021731; ORF025126; ORF085760
GGVTIAQGGVLPNIQAV	ORF067172	1	43.26	4.30	4.30	1	None	ORF016539; ORF020361; ORF020365; ORF020604; ORF020632; ORF020636; ORF020638; ORF020641; ORF020717; ORF020720; ORF020908; ORF020913; ORF020951; ORF020954; ORF021008; ORF021012; ORF021015; ORF021020; ORF021386; ORF021497; ORF021500; ORF021528; ORF021529; ORF021532; ORF021534; ORF021542; ORF021563; ORF021731; ORF085760
GHSEQATQHVSGLQ	ORF060338; ORF060364	2	47.26	0.70	1.70	2	None	ORF002404; ORF060337; ORF060344; ORF060345; ORF060349; ORF060350; ORF060353; ORF060354; ORF060356
GIADDYDKK	ORF095859	1	25.77	8.80	8.80	1	None	ORF013742; ORF023722; ORF058081; ORF058082
GIGTISNEEVTK	ORF062221; ORF062223; ORF062226; ORF062227; ORF062229; ORF062232; ORF062233; ORF062236	4	46.39	6.35	14.30	4	None	ORF014920
GIGTISNEEVTKIA	ORF062221; ORF062223; ORF062226; ORF062227; ORF062229; ORF062232; ORF062233; ORF062236	3	46.67	-4.80	4.80	3	None	ORF014920
GISSAAGGTAPKPNVIT	ORF053999	1	42.96	-1.10	1.10	1	None	ORF012810; ORF054010; ORF054012
GISSAAGGTAPKPNVITK	ORF053999	1	47.46	-4.50	4.50	1	None	ORF012810; ORF054010; ORF054012

Peptide sequence	Incomplete ORF(s)	PSMs (n)	Best −10LgP	Median ppm	Max ∣ppm∣	Replicates (n)	PTM	Complete ORF match(es)
GKNVDSEEANKLT	ORF062221; ORF062223; ORF062226; ORF062227; ORF062229; ORF062232; ORF062233; ORF062236	3	47.25	0.80	2.00	3	None	ORF014920
GKYKEETIEKM(+15.99)QE	ORF040982	12	47.44	0.20	5.10	6	Oxidation (M)	ORF008943
GKYKEETIEKMQE	ORF040982	12	47.44	0.20	5.10	6	Oxidation (M)	ORF008943
GLDNKLREDLERLK	ORF071696	1	44.29	0.40	0.40	1	None	ORF018184
GLSFETTDESLR	ORF031536; ORF031542	5	40.41	1.40	7.60	5	None	ORF004221
GLWEIENNPTVK	ORF101777; ORF101779; ORF101782; ORF101784	4	43.15	1.95	8.00	4	None	ORF025356
GPIDPISPSVT	ORF062221; ORF062223; ORF062226; ORF062227; ORF062229; ORF062232; ORF062233; ORF062236	1	27.48	-2.50	2.50	1	None	ORF014920
GPIFNVIGEAAAKYQPDPPPPR	ORF073021	3	47.11	0.00	7.50	2	None	ORF017885
GPKDIVAYEETKA	ORF101777; ORF101779; ORF101782; ORF101784	2	41.06	-6.75	9.70	2	None	ORF025356
GPPYHDPFS	ORF080397	1	29.83	3.00	3.00	1	None	ORF019503
GPPYHDPFSDPYVPI	ORF080397	1	27.61	0.80	0.80	1	None	ORF019503
GPQYPGAPAPGQPTDPK	ORF100266; ORF100267; ORF100268	3	41.73	-7.30	8.40	3	None	ORF024560
GPQYPGAPAPGQPTDPKVA	ORF100266; ORF100267; ORF100268	1	44.94	-6.40	6.40	1	None	ORF024560
GPQYPGAPAPGQPTDPKVAG	ORF100266; ORF100267; ORF100268	8	47.45	-5.15	12.80	6	None	ORF024560
GPSASADAKKVPTTRPAPLN	ORF053999	2	47.11	2.85	3.30	2	None	ORF012810; ORF054010; ORF054012
GPVREGDVLT	ORF041048	2	27.38	1.15	4.50	2	None	ORF003580
GPVREGDVLTLLE	ORF041048	1	27.98	-0.70	0.70	1	None	ORF003580
GPVREGDVLTLLESER	ORF041048	9	46.18	-1.10	6.10	5	None	ORF003580
GPVREGDVLTLLESEREA	ORF041048	20	47.11	-1.40	5.80	6	None	ORF003580
GPVREGDVLTLLESEREAR	ORF041048	5	42.11	-4.60	5.60	3	None	ORF003580
GPVREGDVLTLLESEREARR	ORF041048	12	46.41	-1.25	3.90	6	None	ORF003580
GPVREGDVLTLLESEREARRL	ORF041048	6	47.01	-0.45	1.40	6	None	ORF003580
GPVREGDVLTLLESEREARRLR	ORF041048	3	43.50	0.20	2.70	3	None	ORF003580
GQAGGAGKFI	ORF089382	1	39.08	-4.10	4.10	1	None	ORF021680; ORF089369; ORF089372; ORF089373; ORF089377; ORF089379; ORF089383
GQGGGGGGGVNVGNLIGGLSS	ORF089382	2	39.44	3.90	3.90	1	None	ORF021680; ORF089369; ORF089372; ORF089373; ORF089377; ORF089379; ORF089383
GQSRGAGGQGGGGAASNI	ORF089382	1	44.74	2.00	2.00	1	None	ORF021680; ORF089369; ORF089372; ORF089373; ORF089377; ORF089379; ORF089383
GQSRGAGGQGGGGAASNILG	ORF089382	1	37.55	-16.70	16.70	1	None	ORF021680; ORF089369; ORF089372; ORF089373; ORF089377; ORF089379; ORF089383
GSGQAGGAGKFIK	ORF089382	6	47.44	3.20	9.60	6	None	ORF021680; ORF089369; ORF089372; ORF089373; ORF089377; ORF089379; ORF089383
GSGQAGGAGKFIKGAIDTLS	ORF089382	4	46.61	-0.90	0.90	1	None	ORF021680; ORF089369; ORF089372; ORF089373; ORF089377; ORF089379; ORF089383
GSGQGGGGGGGVNVGNLIGGLS	ORF089382	2	46.00	0.90	3.00	2	None	ORF021680; ORF089369; ORF089372; ORF089373; ORF089377; ORF089379; ORF089383

Peptide sequence	Incomplete ORF(s)	PSMs (n)	Best −10LgP	Median ppm	Max ∣ppm∣	Replicates (n)	PTM	Complete ORF match(es)
GSGQGGGGGGGVNVGNLIGGLSS	ORF089382	1	46.65	-4.80	4.80	1	None	ORF021680; ORF089369; ORF089372; ORF089373; ORF089377; ORF089379; ORF089383
GSGQSRGAGGQGGGGAASNI	ORF089382	4	47.46	0.85	4.10	3	None	ORF021680; ORF089369; ORF089372; ORF089373; ORF089377; ORF089379; ORF089383
GSGQSRGAGGQGGGGAASNILG	ORF089382	1	29.36	-2.20	2.20	1	None	ORF021680; ORF089369; ORF089372; ORF089373; ORF089377; ORF089379; ORF089383
GSRIEEGVPQH	ORF062221; ORF062223; ORF062226; ORF062227; ORF062229; ORF062232; ORF062233; ORF062236	2	25.18	-1.90	2.30	2	None	ORF014920
GSRIEEGVPQHL	ORF062221; ORF062223; ORF062226; ORF062227; ORF062229; ORF062232; ORF062233; ORF062236	2	38.76	6.10	9.60	2	None	ORF014920
GSRIEEGVPQHLVLLTGGKSVDDVRGAA	ORF062221; ORF062223; ORF062226; ORF062227; ORF062229; ORF062232; ORF062233; ORF062236	3	47.45	2.10	4.30	2	None	ORF014920
GSTRTGSDGLVNIR	ORF062221; ORF062223; ORF062226; ORF062227; ORF062229; ORF062232; ORF062233; ORF062236	1	44.77	-2.40	2.40	1	None	ORF014920
GTEDSIHIR	ORF095859	2	45.76	-0.60	0.60	1	None	ORF023722
GTHETAYLGPKDIVA	ORF101777; ORF101779; ORF101782; ORF101784	3	47.33	-5.40	17.00	3	None	ORF025356
GTHETAYLGPKDIVAYE	ORF101777; ORF101779; ORF101782; ORF101784	1	38.83	2.50	2.50	1	None	ORF025356
GTHETAYLGPKDIVAYEETKA	ORF101777; ORF101779; ORF101782; ORF101784	1	45.63	2.70	2.70	1	None	ORF025356
GTSLNIGKALD	ORF062221; ORF062223; ORF062226; ORF062227; ORF062229; ORF062232; ORF062233; ORF062236	1	39.79	-12.60	12.60	1	None	ORF014920
GTSLNIGKALDYVAK	ORF062221; ORF062223; ORF062226; ORF062227; ORF062229; ORF062232; ORF062233; ORF062236	1	32.62	-1.90	1.90	1	None	ORF014920
GTVVEHPEYGEVIQLQGDQR	ORF095859	1	46.01	-3.80	3.80	1	None	ORF023722
GVEPAADGKGVIV	ORF091776; ORF091777	2	44.74	-1.45	7.80	2	None	ORF021747; ORF022702
GVEPAADGKGVIVV	ORF091776; ORF091777	8	47.21	2.05	3.60	6	None	ORF021747; ORF022702
GVEPAADGKGVIVVL	ORF091776; ORF091777	1	31.13	6.00	6.00	1	None	ORF021747; ORF022702
GVEPAADGKGVIVVLK	ORF091776; ORF091777	16	47.45	-0.50	8.60	6	None	ORF021747; ORF022702
GVHDNTDIVLLQE	ORF050024	2	38.81	0.15	2.00	2	None	ORF010954
GVLSDQLYHWTGSD	ORF090968	1	31.88	-1.20	1.20	1	None	ORF021892
GVTIAQGGVLPNIQ	ORF067172	4	43.01	-1.90	3.40	3	None	ORF016539; ORF020361; ORF020365; ORF020604; ORF020632; ORF020636; ORF020638; ORF020641; ORF020717; ORF020720; ORF020908; ORF020913; ORF020951; ORF020954; ORF021008; ORF021012; ORF021015; ORF021020; ORF021386; ORF021497; ORF021500; ORF021528; ORF021529; ORF021532; ORF021534; ORF021542; ORF021563; ORF021731; ORF025126; ORF085760

Peptide sequence	Incomplete ORF(s)	PSMs (n)	Best −10LgP	Median ppm	Max ∣ppm∣	Replicates (n)	PTM	Complete ORF match(es)
GYPHWPARIDEILEPAA	ORF101777; ORF101779; ORF101782; ORF101784	8	41.59	-3.50	4.20	5	None	ORF025356
GYPHWPARIDEILEPAAKS	ORF101777; ORF101779; ORF101782; ORF101784	1	30.48	-0.20	0.20	1	None	ORF025356
HDNTDIVLLQE	ORF050024	2	27.01	0.65	1.40	2	None	ORF010954
HDPFSDPYVPI	ORF080397	1	28.84	-1.30	1.30	1	None	ORF019503
HETAYLGPKDIVA	ORF101777; ORF101779; ORF101782; ORF101784	3	46.62	0.60	5.50	3	None	ORF025356
HGDIITIIDR	ORF087831	1	32.88	-5.20	5.20	1	None	ORF021301; ORF087775; ORF087778; ORF087823
HGYTAAYSR	ORF066774; ORF066779	3	36.40	2.60	5.10	3	None	ORF016089
HLLPPPPPLPS	ORF080397	1	37.82	-1.90	1.90	1	None	ORF019503
HNNRDIIMDAVTKAE	ORF062221; ORF062223; ORF062226; ORF062227; ORF062229; ORF062232; ORF062233; ORF062236	1	45.39	-0.50	0.50	1	None	ORF014920
HNVKELEV	ORF066964	1	37.27	0.10	0.10	1	None	ORF010381; ORF016315
HSEQATQHVSGL	ORF060338; ORF060364	1	45.14	5.40	5.40	1	None	ORF002404; ORF060337; ORF060344; ORF060345; ORF060349; ORF060350; ORF060353; ORF060354; ORF060356
HSEQATQHVSGLQ	ORF060338; ORF060364	5	47.25	0.20	6.80	5	None	ORF002404; ORF060337; ORF060344; ORF060345; ORF060349; ORF060350; ORF060353; ORF060354; ORF060356
HSQNGFIVAPPPEK	ORF038565	1	26.07	2.80	2.80	1	None	ORF007809
HSTKDEVQSAVR	ORF062221; ORF062223; ORF062226; ORF062227; ORF062229; ORF062232; ORF062233; ORF062236	4	46.53	0.35	4.70	4	None	ORF014920
HWPARIDEILEPAA	ORF101777; ORF101779; ORF101782; ORF101784	3	36.15	-3.70	5.10	3	None	ORF025356
IAFAITAIK	ORF071696	3	41.27	-2.70	5.70	3	None	ORF018184
IAPVIDEDPIEEIRQ	ORF053166	7	46.67	-2.80	6.40	5	None	ORF003049; ORF053159; ORF053173; ORF053180
IAPVIDEDPIEEIRQES	ORF053166	2	45.84	-3.10	3.10	1	None	ORF003049; ORF053159; ORF053173; ORF053180
IAQGGVLPNIQ	ORF067172	2	32.75	-1.60	2.10	2	None	ORF016539; ORF020361; ORF020365; ORF020604; ORF020626; ORF020632; ORF020636; ORF020638; ORF020641; ORF020717; ORF020720; ORF020908; ORF020913; ORF020951; ORF020954; ORF021008; ORF021012; ORF021015; ORF021020; ORF021386; ORF021497; ORF021500; ORF021528; ORF021529; ORF021532; ORF021534; ORF021542; ORF021563; ORF021731; ORF025126; ORF085760
IAQGGVLPNIQAVLLPKK	ORF067172	3	47.14	-3.80	4.50	2	None	ORF016539; ORF020361; ORF020365; ORF020604; ORF020626; ORF020632; ORF020636; ORF020638; ORF020641; ORF020717; ORF020720; ORF020908; ORF020913; ORF020951; ORF020954; ORF021008; ORF021012; ORF021015; ORF021020; ORF021386; ORF021497; ORF021500; ORF021528; ORF021529; ORF021532; ORF021534; ORF021542; ORF021563; ORF021731; ORF085760
IDDRLEQYTHA	ORF079438; ORF079444	1	25.79	2.60	2.60	1	None	ORF020265; ORF079440
IDDRLEQYTHAIE	ORF079438; ORF079444	1	29.76	-2.40	2.40	1	None	ORF020265; ORF079440
IDDRLEQYTHAIET	ORF079438; ORF079444	8	47.16	-1.05	5.10	4	None	ORF020265; ORF079440
IDDRLEQYTHAIETSIK	ORF079438; ORF079444	1	46.95	-2.40	2.40	1	None	ORF020265; ORF079440

Peptide sequence	Incomplete ORF(s)	PSMs (n)	Best −10LgP	Median ppm	Max ∣ppm∣	Replicates (n)	PTM	Complete ORF match(es)
IDDRLEQYTHAIETSIKPGR	ORF079438; ORF079444	3	45.75	0.50	2.00	3	None	ORF020265; ORF079440
IDDRLEQYTHAIETSIKPGRLA	ORF079438; ORF079444	5	47.20	2.40	3.00	4	None	ORF020265; ORF079440
IDEGARQIK	ORF062221; ORF062223; ORF062226; ORF062227; ORF062229; ORF062232; ORF062233; ORF062236	3	37.81	2.80	6.20	3	None	ORF014920
IDEILEPAAK	ORF101777; ORF101779; ORF101782; ORF101784	3	41.37	-3.60	7.70	3	None	ORF025356
IDEILEPAAKS	ORF101777; ORF101779; ORF101782; ORF101784	1	30.50	-1.80	1.80	1	None	ORF025356
IDGSNDGRSSFPS	ORF062221; ORF062223; ORF062226; ORF062227; ORF062229; ORF062232; ORF062233; ORF062236	1	32.00	1.50	1.50	1	None	ORF014920
IDGSRDATPEFNNVK	ORF062221; ORF062223; ORF062226; ORF062227; ORF062229; ORF062232; ORF062233; ORF062236	4	43.52	1.60	3.60	3	None	ORF014920
IDGSRDATPEFNNVKE	ORF062221; ORF062223; ORF062226; ORF062227; ORF062229; ORF062232; ORF062233; ORF062236	1	46.76	-1.60	1.60	1	None	ORF014920
IDGSRDATPEFNNVKELIGR	ORF062221; ORF062223; ORF062226; ORF062227; ORF062229; ORF062232; ORF062233; ORF062236	3	42.56	-4.50	4.80	2	None	ORF014920
IDGYPREVKQGEEFEK	ORF076196	1	28.87	7.80	7.80	1	None	ORF018471; ORF076197; ORF076198
IDTLSGGGGGGQAGGAGK	ORF089382	8	47.44	1.90	3.60	6	None	ORF021680; ORF089369; ORF089372; ORF089373; ORF089377; ORF089379; ORF089383
IDTLSGGGGGGQAGGAGKL	ORF089382	1	46.64	-7.30	7.30	1	None	ORF021680; ORF089369; ORF089372; ORF089373; ORF089377; ORF089379; ORF089383
IDVAKVNTLIRPDGEK	ORF067090	5	47.45	-0.10	1.30	5	None	ORF006342; ORF016564
IEDNNTLVFIVDVK	ORF067090	11	47.46	2.50	3.50	5	None	ORF006342; ORF016564
IEDNNTLVFIVDVKANK	ORF067090	12	47.46	-0.10	3.00	6	None	ORF006342; ORF016564
IEEGVPQH	ORF062221; ORF062223; ORF062226; ORF062227; ORF062229; ORF062232; ORF062233; ORF062236	5	39.80	2.10	4.80	4	None	ORF014920
IEEGVPQHL	ORF062221; ORF062223; ORF062226; ORF062227; ORF062229; ORF062232; ORF062233; ORF062236	3	43.82	-0.10	3.30	3	None	ORF014920
IEEGVPQHLV	ORF062221; ORF062223; ORF062226; ORF062227; ORF062229; ORF062232; ORF062233; ORF062236	4	33.57	0.25	4.30	4	None	ORF014920
IEEGVPQHLVLLTGGKSVDDVRGAA	ORF062221; ORF062223; ORF062226; ORF062227; ORF062229; ORF062232; ORF062233; ORF062236	3	40.11	2.00	2.20	2	None	ORF014920
IEEIKDFL	ORF098458	4	36.93	-1.65	2.00	4	None	ORF023230
IEEIKDFLL	ORF098458	2	27.36	3.10	3.20	2	None	ORF023230
IEEIKDFLLT	ORF098458	9	35.34	0.90	2.30	6	None	ORF023230
IEEIKDFLLTA	ORF098458	5	46.92	0.40	3.00	4	None	ORF023230

Peptide sequence	Incomplete ORF(s)	PSMs (n)	Best −10LgP	Median ppm	Max ∣ppm∣	Replicates (n)	PTM	Complete ORF match(es)
IEEIKDFLLTAR	ORF098458	7	45.83	0.20	2.80	3	None	ORF023230
IETIVTDPR	ORF062221; ORF062223; ORF062226; ORF062227; ORF062229; ORF062232; ORF062233; ORF062236	2	35.26	1.10	2.60	2	None	ORF014920
IGGLSFETTDESLR	ORF031536; ORF031542	6	47.13	0.25	1.30	5	None	ORF004221
IGGLSFETTDESLRNH	ORF031536; ORF031542	1	47.00	0.00	0.00	1	None	ORF004221
IGIGTISNEEVTK	ORF062221; ORF062223; ORF062226; ORF062227; ORF062229; ORF062232; ORF062233; ORF062236	7	47.26	-2.00	9.40	6	None	ORF014920
IGIGTISNEEVTKIA	ORF062221; ORF062223; ORF062226; ORF062227; ORF062229; ORF062232; ORF062233; ORF062236	4	47.15	-0.20	11.40	2	None	ORF014920
IGIGTISNEEVTKIAS	ORF062221; ORF062223; ORF062226; ORF062227; ORF062229; ORF062232; ORF062233; ORF062236	2	47.13	1.00	1.00	1	None	ORF014920
IGKNVDSEEANKLT	ORF062221; ORF062223; ORF062226; ORF062227; ORF062229; ORF062232; ORF062233; ORF062236	19	47.26	0.90	4.30	6	None	ORF014920
IGKNVDSEEANKLTLS	ORF062221; ORF062223; ORF062226; ORF062227; ORF062229; ORF062232; ORF062233; ORF062236	5	47.11	-3.90	5.60	5	None	ORF014920
IGTISNEEVTK	ORF062221; ORF062223; ORF062226; ORF062227; ORF062229; ORF062232; ORF062233; ORF062236	2	32.47	-8.00	17.40	2	None	ORF014920
IGTISNEEVTKIA	ORF062221; ORF062223; ORF062226; ORF062227; ORF062229; ORF062232; ORF062233; ORF062236	4	47.20	1.55	2.60	4	None	ORF014920
IGVRNADTTELQ	ORF062221; ORF062223; ORF062226; ORF062227; ORF062229; ORF062232; ORF062233; ORF062236	1	45.42	3.90	3.90	1	None	ORF014920
IGVRNADTTELQTIS	ORF062221; ORF062223; ORF062226; ORF062227; ORF062229; ORF062232; ORF062233; ORF062236	1	45.91	-14.10	14.10	1	None	ORF014920
IGVVQYSDTTRPNFQ	ORF062221; ORF062223; ORF062226; ORF062227; ORF062229; ORF062232; ORF062233; ORF062236	4	46.85	-1.95	7.90	3	None	ORF014920
IITGAKSVSDGQSAALA	ORF062221; ORF062223; ORF062226; ORF062227; ORF062229; ORF062232; ORF062233; ORF062236	1	44.33	9.10	9.10	1	None	ORF014920
IIVPQGPPKPTPK	ORF066708	11	47.03	-1.10	8.50	6	None	ORF016200; ORF066693; ORF066716
IKEAEATKDEFGR	ORF044453	1	25.48	4.20	4.20	1	None	ORF008679
IKEFKELPSIESI	ORF062221; ORF062223; ORF062226; ORF062227; ORF062229; ORF062232; ORF062233; ORF062236	2	44.80	-3.35	4.80	2	None	ORF014920

Peptide sequence	Incomplete ORF(s)	PSMs (n)	Best −10LgP	Median ppm	Max |ppm|	Replicates (n)	PTM	Complete ORF match(es)
ILELAGNAARDNK	ORF067172	1	42.16	11.60	11.60	1	None	ORF008470; ORF016539; ORF020361; ORF020365; ORF020604; ORF020626; ORF020632; ORF020636; ORF020638; ORF020641; ORF020717; ORF020720; ORF020908; ORF020913; ORF020951; ORF020954; ORF021008; ORF021012; ORF021015; ORF021020; ORF021411; ORF021497; ORF021500; ORF021528; ORF021529; ORF021532; ORF021534; ORF021542; ORF021563; ORF021731; ORF041549; ORF085760
INARADEDVEPDVIVDK	ORF036644; ORF036647	1	46.39	-3.50	3.50	1	None	ORF008344; ORF036655; ORF036666; ORF036670; ORF036673
INARADEDVEPDVIVDKVA	ORF036644; ORF036647	2	46.57	2.50	4.10	2	None	ORF008344; ORF036655; ORF036666; ORF036670; ORF036673
IPDWFLNR	ORF071696	2	26.85	-2.15	2.80	2	None	ORF018184
IPDWFLNRQKDIK	ORF071696	4	46.76	1.20	1.60	2	None	ORF018184
IPFAIGVR	ORF062221; ORF062223; ORF062226; ORF062227; ORF062229; ORF062232; ORF062233; ORF062236	3	26.79	-1.10	2.70	3	None	ORF014920
IQRVVEGLDVDKDKIR	ORF062221; ORF062223; ORF062226; ORF062227; ORF062229; ORF062232; ORF062233; ORF062236	1	30.02	3.20	3.20	1	None	ORF014920
IRKDDEVQVVR	ORF040982	6	46.32	1.60	3.90	3	None	ORF008943
IRLNNQNALNA	ORF044453	2	30.89	1.10	6.40	2	None	ORF008677; ORF008679
IRLNNQNALNAR	ORF044453	2	46.32	-6.35	13.80	2	None	ORF008677; ORF008679
ISSAAGGTAPKPNVIT	ORF053999	4	47.22	2.65	5.70	4	None	ORF012810; ORF054010; ORF054012
ISSAAGGTAPKPNVITK	ORF053999	4	47.46	-1.25	2.90	2	None	ORF012810; ORF054010; ORF054012
IVPFLIGKNVDSEEANKLT	ORF062221; ORF062223; ORF062226; ORF062227; ORF062229; ORF062232; ORF062233; ORF062236	3	46.85	-3.40	7.20	2	None	ORF014920
IVPKTDVETIVTVQG	ORF044453	1	39.20	-2.50	2.50	1	None	ORF008679
IVTHPTLLQDPDVREFLEKDELPR	ORF038565	1	43.08	4.10	4.10	1	None	ORF007809
IYTGVLSDQLYHWTGSD	ORF090968	1	28.96	-1.00	1.00	1	None	ORF021892
KADIDLSKRAGELTEDEVERVVT	ORF071696	2	47.13	3.10	3.10	1	None	ORF018184
KADIDLSKRAGELTEDEVERVVTI	ORF071696	1	26.66	-4.90	4.90	1	None	ORF018184
KALDYVAKNQFVK	ORF062221; ORF062223; ORF062226; ORF062227; ORF062229; ORF062232; ORF062233; ORF062236	1	42.25	1.50	1.50	1	None	ORF014920
KATETPAANSDQTVAVQE	ORF057182	1	37.52	-6.20	6.20	1	None	ORF012631; ORF057183
KDDEVQVVR	ORF040982	6	46.42	0.85	5.00	6	None	ORF008943; ORF022249
KDSPDFSVSLQEITENETLTPELK	ORF061770	1	30.49	6.50	6.50	1	None	ORF015690; ORF061765; ORF061767
KEAEATKDEFGR	ORF044453	1	40.54	9.70	9.70	1	None	ORF008679
KEETIEKMQE	ORF040982	2	47.18	4.60	5.20	2	None	ORF008943

Peptide sequence	Incomplete ORF(s)	PSMs (n)	Best −10LgP	Median ppm	Max ∣ppm∣	Replicates (n)	PTM	Complete ORF match(es)
KEFIYGVSSFQ	ORF062221; ORF062223; ORF062226; ORF062227; ORF062229; ORF062232; ORF062233; ORF062236	3	46.22	9.10	12.00	2	None	ORF014920
KEFKELPSIESI	ORF062221; ORF062223; ORF062226; ORF062227; ORF062229; ORF062232; ORF062233; ORF062236	2	43.97	1.15	2.20	2	None	ORF014920
KELPSIESI	ORF062221; ORF062223; ORF062226; ORF062227; ORF062229; ORF062232; ORF062233; ORF062236	3	37.30	-3.50	3.80	3	None	ORF014920
KELPSIESIL	ORF062221; ORF062223; ORF062226; ORF062227; ORF062229; ORF062232; ORF062233; ORF062236	3	34.52	-5.40	6.50	2	None	ORF014920
KEQTGELGDQDEPLT	ORF078612	2	47.11	12.80	12.80	1	None	ORF019339; ORF078617; ORF078626; ORF078629; ORF078634
KEQTGELGDQDEPLTQN	ORF078612	1	44.82	2.50	2.50	1	None	ORF019339; ORF078617; ORF078626; ORF078629; ORF078634
KEQTGELGDQDEPLTQNDIR	ORF078612	8	47.46	1.30	5.70	6	None	ORF019339; ORF078617; ORF078626; ORF078629; ORF078634
KEQTGELGDQDEPLTQNDIR NTKDVDM(+15.99)K	ORF078612	11	47.46	4.30	12.10	6	Oxidation (M)	ORF019339; ORF078617; ORF078626; ORF078629; ORF078634
KEQTGELGDQDEPLTQNDIR NTKDVDM(+15.99)KIGV	ORF078612	30	47.46	-0.30	4.20	6	Oxidation (M)	ORF019339; ORF078617; ORF078626; ORF078629; ORF078634
KEQTGELGDQDEPLTQNDIR NTKDVDMK	ORF078612	11	47.46	4.30	12.10	6	Oxidation (M)	ORF019339; ORF078617; ORF078626; ORF078629; ORF078634
KEQTGELGDQDEPLTQNDIR NTKDVDMKIGV	ORF078612	30	47.46	-0.30	4.20	6	Oxidation (M)	ORF019339; ORF078617; ORF078626; ORF078629; ORF078634
KESLNPLDLEILKEK	ORF086999	4	46.77	-1.90	4.50	4	None	ORF021062
KGAIDTLSGGGGGGQAGGA GK	ORF089382	1	47.15	8.10	8.10	1	None	ORF021680; ORF089369; ORF089372; ORF089373; ORF089377; ORF089379; ORF089383
KGDDWLPAGTEDSIHIR	ORF095859	2	47.23	1.05	1.80	2	None	ORF023722
KGFSEGLWEIENNPTVK	ORF101777; ORF101779; ORF101782; ORF101784	27	47.05	-0.80	3.20	6	None	ORF025356
KGFSEGLWEIENNPTVKA	ORF101777; ORF101779; ORF101782; ORF101784	6	46.93	-2.50	10.60	5	None	ORF025356
KGLLDIIH	ORF033209	11	36.99	-1.10	1.90	6	None	ORF001812
KGPVREGDVLTLLESEREA	ORF041048	1	39.83	-2.50	2.50	1	None	ORF003580
KIEDNNTLVFIVDVK	ORF067090	8	47.14	-1.70	5.40	6	None	ORF006342; ORF016564
KIEDNNTLVFIVDVKANK	ORF067090	4	47.36	-3.10	6.60	4	None	ORF006342; ORF016564
KIEEIKDFL	ORF098458	1	41.91	3.40	3.40	1	None	ORF023230
KIEEIKDFLLT	ORF098458	2	27.50	1.00	4.50	2	None	ORF023230
KKGLLDIIH	ORF033209	7	44.71	0.80	2.40	5	None	ORF001812
KLFIGGLSFETTDESLR	ORF031536; ORF031542	6	46.75	1.00	7.30	5	None	ORF004221
KLYNIDVAKVNTLIRPDGEK	ORF067090	1	26.80	2.70	2.70	1	None	ORF016564
KNLTDADLIQLLQNYGIK	ORF076580; ORF076584	2	35.00	-4.70	6.40	2	None	ORF018429; ORF076577; ORF076579; ORF076582

Peptide sequence	Incomplete ORF(s)	PSMs (n)	Best −10LgP	Median ppm	Max ∣ppm∣	Replicates (n)	PTM	Complete ORF match(es)
KNVDSEEANKLT	ORF062221; ORF062223; ORF062226; ORF062227; ORF062229; ORF062232; ORF062233; ORF062236	2	46.56	0.25	2.10	2	None	ORF014920
KNVDSEEANKLTLSKEFIYG	ORF062221; ORF062223; ORF062226; ORF062227; ORF062229; ORF062232; ORF062233; ORF062236	1	36.64	-13.60	13.60	1	None	ORF014920
KPSQISKPTPEAPADVDNAAD LLSQ	ORF055592	2	31.82	2.65	2.70	2	None	ORF013351
KPSTAPASKPIAAPK	ORF053999	2	47.38	1.55	2.80	2	None	ORF012810; ORF054010; ORF054012
KPVPPAAPKPAASKPADTKE AAQKQ	ORF053999	1	40.32	-4.90	4.90	1	None	ORF012810; ORF054001; ORF054006; ORF054010; ORF054012; ORF054013; ORF054018
KPVSSYEKITINK	ORF091776; ORF091777	2	47.04	-4.55	5.90	2	None	ORF021747; ORF022702
KPVSSYEKITINKN	ORF091776; ORF091777	1	44.23	-10.50	10.50	1	None	ORF021747; ORF022702
KQLEDGRTL	ORF082494; ORF082495; ORF082496; ORF082497; ORF082498	2	26.31	2.05	6.00	2	None	ORF001027; ORF001195; ORF005506; ORF005510; ORF011164; ORF107843; ORF107844; ORF107845; ORF107846; ORF107847; ORF107848; ORF107849; ORF107850; ORF107851; ORF107852; ORF107853; ORF107854; ORF107855; ORF107856; ORF107857; ORF107858; ORF107859; ORF107860
KRAGELTEDEVERVVT	ORF071696	2	47.26	-0.95	2.00	2	None	ORF018184
KRAGELTEDEVERVVTIM	ORF071696	2	26.40	-0.90	1.50	2	None	ORF018184
KRAGELTEDEVERVVTIMQN PR	ORF071696	1	43.91	-3.90	3.90	1	None	ORF018184
KRAGELTEDEVERVVTIMQN PRQ	ORF071696	3	41.91	-3.60	3.60	2	None	ORF018184
KSEEPEQLR	ORF031536	2	34.79	7.95	13.80	2	None	ORF004221
KTDDIEILR	ORF062221; ORF062223; ORF062226; ORF062227; ORF062229; ORF062232; ORF062233; ORF062236	4	28.83	4.30	7.80	4	None	ORF014920
KTITTVQTAAKEAISPTLE	ORF100055; ORF100073; ORF100075; ORF100076; ORF100077; ORF100078; ORF100079	3	44.80	-1.70	2.80	3	None	ORF025240
KTVGVEPAADGKGVIV	ORF091776; ORF091777	2	46.02	-5.05	5.70	2	None	ORF021747; ORF022702
KTVGVEPAADGKGVIVV	ORF091776; ORF091777	4	47.22	-0.70	2.10	3	None	ORF021747; ORF022702
KTVGVEPAADGKGVIVVL	ORF091776; ORF091777	1	28.86	-3.90	3.90	1	None	ORF021747; ORF022702
KTVGVEPAADGKGVIVVLK	ORF091776; ORF091777	8	47.46	3.40	4.10	6	None	ORF021747; ORF022702
KVAPPPIPPPPKEESFA	ORF086801	1	37.06	-8.10	8.10	1	None	ORF020966; ORF086789; ORF086800
KVDASNSVDQVFVNVQ	ORF069950	2	46.97	-2.60	4.50	2	None	ORF016799; ORF069954; ORF069958; ORF069960
KVDASNSVDQVFVNVQK	ORF069950	19	47.46	-1.20	7.20	6	None	ORF016799; ORF069954; ORF069958; ORF069960
KVDASNSVDQVFVNVQKIFD REG	ORF069950	11	47.39	-2.90	5.00	6	None	ORF016799; ORF069954; ORF069958; ORF069960
KVPAATSPRKPLS	ORF053999	1	37.06	2.60	2.60	1	None	ORF012810; ORF054010; ORF054012
KYKEETIEKMQE	ORF040982	4	41.88	3.60	14.20	2	None	ORF008943

Peptide sequence	Incomplete ORF(s)	PSMs (n)	Best −10LgP	Median ppm	Max ∣ppm∣	Replicates (n)	PTM	Complete ORF match(es)
LAVATSGADRTEIETIVTDPR	ORF062221; ORF062223; ORF062226; ORF062227; ORF062229; ORF062232; ORF062233; ORF062236	2	47.11	1.05	6.40	2	None	ORF014920
LDDRADALQAGASQFETSAA	ORF044627	1	33.09	3.10	3.10	1	None	ORF009505; ORF022326; ORF092996; ORF093003
LDHYAIIKFPL	ORF067090	3	29.62	-8.50	8.50	2	None	ORF006342; ORF016564
LDHYAIIKFPLTTESAM	ORF067090	3	40.82	-3.00	5.50	1	Oxidation (M)	ORF006342; ORF016564
LDHYAIIKFPLTTESAM(+15.99)	ORF067090	3	40.82	-3.00	5.50	1	Oxidation (M)	ORF006342; ORF016564
LDVANKIGII	ORF067090	1	43.25	-2.70	2.70	1	None	ORF006342; ORF016564
LDYDFQRDYYESYSAARVPPPPPIA	ORF069609	1	27.72	-5.20	5.20	1	None	ORF001221; ORF069605; ORF069606; ORF069607; ORF069608
LEDYISGKSNGEAEGIGKTVDV	ORF052086	1	46.93	-4.40	4.40	1	None	ORF012186; ORF052076
LEGHSEQATQHVSG	ORF060338; ORF060364	1	41.89	-5.80	5.80	1	None	ORF002404; ORF060337; ORF060344; ORF060345; ORF060349; ORF060350; ORF060353; ORF060354; ORF060356; ORF060365
LEGHSEQATQHVSGL	ORF060338; ORF060364	1	39.58	5.10	5.10	1	None	ORF002404; ORF060337; ORF060344; ORF060345; ORF060349; ORF060350; ORF060353; ORF060354; ORF060356
LEGHSEQATQHVSGLQ	ORF060338; ORF060364	5	46.90	2.80	9.70	5	None	ORF002404; ORF060337; ORF060344; ORF060345; ORF060349; ORF060350; ORF060353; ORF060354; ORF060356
LELLSPTDPVSNIK	ORF079438; ORF079444	2	38.50	-3.55	9.10	2	None	ORF020265; ORF079440
LENNPPVQK	ORF086801	1	43.53	-8.60	8.60	1	None	ORF020966; ORF086783; ORF086789; ORF086800
LFIGGLSFETTDESLR	ORF031536; ORF031542	1	29.59	2.10	2.10	1	None	ORF004221
LGGVTIAQGGVLPNIQ	ORF067172	1	39.56	-4.40	4.40	1	None	ORF016539; ORF020361; ORF020365; ORF020604; ORF020632; ORF020636; ORF020638; ORF020641; ORF020717; ORF020720; ORF020908; ORF020913; ORF020951; ORF020954; ORF021008; ORF021012; ORF021015; ORF021020; ORF021386; ORF021497; ORF021500; ORF021528; ORF021529; ORF021532; ORF021534; ORF021542; ORF021563; ORF021731; ORF025126; ORF085760
LGISSAAGGTAPKPNVIT	ORF053999	4	47.18	1.65	5.20	4	None	ORF012810; ORF054010; ORF054012
LGISSAAGGTAPKPNVITK	ORF053999	2	47.45	-2.70	4.80	1	None	ORF012810; ORF054010; ORF054012
LGPKDIVAYEETK	ORF101777; ORF101779; ORF101782; ORF101784	2	27.46	-4.20	9.40	2	None	ORF025356
LGPKDIVAYEETKA	ORF101777; ORF101779; ORF101782; ORF101784	5	45.70	-2.60	5.70	5	None	ORF025356
LIAYIATKE	ORF088248; ORF088249	5	37.72	0.80	5.30	5	None	ORF021197
LIGGLSSLAGSGQSR	ORF089382	1	36.58	-16.30	16.30	1	None	ORF021680; ORF089369; ORF089372; ORF089373; ORF089377; ORF089379; ORF089383
LIGKNVDSEEANKLT	ORF062221; ORF062223; ORF062226; ORF062227; ORF062229; ORF062232; ORF062233; ORF062236	1	39.95	-7.50	7.50	1	None	ORF014920
LKTDDIEIL	ORF062221; ORF062223; ORF062226; ORF062227; ORF062229; ORF062232; ORF062233; ORF062236	1	27.71	-8.40	8.40	1	None	ORF014920

Peptide sequence	Incomplete ORF(s)	PSMs (n)	Best −10LgP	Median ppm	Max ∣ppm∣	Replicates (n)	PTM	Complete ORF match(es)
LKTDDIEILR	ORF062221; ORF062223; ORF062226; ORF062227; ORF062229; ORF062232; ORF062233; ORF062236	1	27.35	0.10	0.10	1	None	ORF014920
LLESEREAR	ORF041048	1	25.17	3.40	3.40	1	None	ORF003580
LLIDTLGGPESVPESPK	ORF083445; ORF083446	3	45.72	-6.40	6.40	2	None	ORF001845
LLQEALESNPDGISTAFPSR	ORF036238; ORF036244; ORF036252	3	46.51	-0.70	6.80	2	None	ORF008268
LLSPTDPVSNIK	ORF079438; ORF079444	2	42.93	-2.15	4.60	2	None	ORF020265; ORF079440
LLSPTDPVSNIKNRWEIGDVTTAA	ORF079438; ORF079444	2	40.33	-2.55	4.30	2	None	ORF020265; ORF079440
LNNQNALNAR	ORF044453	2	38.58	5.40	5.70	2	None	ORF008677; ORF008679
LPAGTEDSIHIR	ORF095859	6	47.35	-2.05	10.20	6	None	ORF023722
LPPIPPPSVSTLSHR	ORF080397	1	47.33	-1.00	1.00	1	None	ORF019503
LPTFKIPL	ORF100055; ORF100073; ORF100075; ORF100078	3	26.43	-3.00	5.90	3	None	ORF025240; ORF100065
LPTPEIKTVAEEDEVK	ORF100073; ORF100075	1	41.84	3.30	3.30	1	None	ORF025240
LRQADFLEGHSEQATQHVSG	ORF060338; ORF060364	4	47.02	5.45	8.20	4	None	ORF002404; ORF060337; ORF060344; ORF060345; ORF060349; ORF060350; ORF060353; ORF060354; ORF060356
LSKEFIYGVSSFQ	ORF062221; ORF062223; ORF062226; ORF062227; ORF062229; ORF062232; ORF062233; ORF062236	1	38.77	12.20	12.20	1	None	ORF014920
LTEDEVERVVTIMQNPR	ORF071696	9	46.94	-2.10	3.50	5	None	ORF018184
LTEDEVERVVTIMQNPRQ	ORF071696	4	45.00	-4.00	4.90	2	None	ORF018184
LVITDKEKAEK	ORF098458	3	47.21	4.90	7.30	2	None	ORF023230
LVITDKEKAEKLK	ORF098458	5	47.43	1.90	3.20	5	None	ORF023230
LWEIENNPTVK	ORF101777; ORF101779; ORF101782; ORF101784	4	25.34	-0.70	2.70	4	None	ORF025356
LYTLVITDKEKAEK	ORF098458	1	45.62	-4.60	4.60	1	None	ORF023230
M(+15.99)DNITHVPGGGHK	ORF095365	2	29.03	4.60	7.20	1	Oxidation (M)	ORF024066; ORF095359; ORF095363; ORF095366
M(+15.99)PIRKDDEVQVVR	ORF040982	12	47.45	2.30	6.40	6	Oxidation (M)	ORF008943
MDNITHVPGGGHK	ORF095365	2	29.03	4.60	7.20	1	Oxidation (M)	ORF024066; ORF095359; ORF095363; ORF095366
MPIRKDDEVQVVR	ORF040982	12	47.45	2.30	6.40	6	Oxidation (M)	ORF008943
MQIFVKTLT	ORF082494; ORF082495; ORF082496; ORF082497; ORF082498	3	30.26	-1.60	10.00	3	None	ORF001027; ORF001195; ORF005506; ORF005510; ORF011164; ORF107843; ORF107844; ORF107845; ORF107846; ORF107847; ORF107848; ORF107849; ORF107850; ORF107851; ORF107852; ORF107853; ORF107854; ORF107855; ORF107856; ORF107857; ORF107858; ORF107859; ORF107860
NAAEDTKALIPVIVVK	ORF029559	3	46.13	-1.20	3.70	3	None	ORF005044
NAHSTKDEVQSAVR	ORF062221; ORF062223; ORF062226; ORF062227; ORF062229; ORF062232; ORF062233; ORF062236	8	47.15	0.05	10.70	6	None	ORF014920
NAILRQAENIKV	ORF050709	1	37.98	-5.80	5.80	1	None	ORF009765; ORF010196; ORF037399; ORF037400
NAILRQAENIKVK	ORF050709	4	47.07	-0.70	2.70	4	None	ORF009765; ORF010196; ORF037399; ORF037400

Peptide sequence	Incomplete ORF(s)	PSMs (n)	Best −10LgP	Median ppm	Max ∣ppm∣	Replicates (n)	PTM	Complete ORF match(es)
NAILRQAENIKVKQ	ORF050709	2	47.16	-1.35	3.50	2	None	ORF009765; ORF010196; ORF037399; ORF037400
NAPDETNVVR	ORF087621; ORF087623	6	45.96	3.30	7.10	5	None	ORF021436
NDEELNKLL	ORF067172	2	29.44	3.45	4.10	2	None	ORF016539; ORF020361; ORF020365; ORF020604; ORF020626; ORF020632; ORF020636; ORF020638; ORF020641; ORF020717; ORF020720; ORF020908; ORF020913; ORF020951; ORF020954; ORF021008; ORF021012; ORF021015; ORF021020; ORF021386; ORF021497; ORF021500; ORF021528; ORF021529; ORF021532; ORF021534; ORF021537; ORF021542; ORF021563; ORF021731; ORF025126; ORF026134; ORF085760
NDEELNKLLG	ORF067172	2	45.57	-0.45	0.80	2	None	ORF016539; ORF020361; ORF020365; ORF020604; ORF020626; ORF020632; ORF020636; ORF020638; ORF020641; ORF020717; ORF020720; ORF020908; ORF020913; ORF020951; ORF020954; ORF021008; ORF021012; ORF021015; ORF021020; ORF021386; ORF021497; ORF021500; ORF021528; ORF021529; ORF021532; ORF021534; ORF021537; ORF021542; ORF021563; ORF021731; ORF025126; ORF026134; ORF085760
NDEELNKLLGGVT	ORF067172	2	42.80	1.25	5.60	2	None	ORF016539; ORF020361; ORF020365; ORF020604; ORF020632; ORF020636; ORF020638; ORF020641; ORF020717; ORF020720; ORF020908; ORF020913; ORF020951; ORF020954; ORF021008; ORF021012; ORF021015; ORF021020; ORF021386; ORF021497; ORF021500; ORF021528; ORF021529; ORF021532; ORF021534; ORF021537; ORF021542; ORF021563; ORF021731; ORF025126; ORF026134; ORF085760
NDEELNKLLGGVTIA	ORF067172	1	46.35	-2.10	2.10	1	None	ORF016539; ORF020361; ORF020365; ORF020604; ORF020632; ORF020636; ORF020638; ORF020641; ORF020717; ORF020720; ORF020908; ORF020913; ORF020951; ORF020954; ORF021008; ORF021012; ORF021015; ORF021020; ORF021386; ORF021497; ORF021500; ORF021528; ORF021529; ORF021532; ORF021534; ORF021537; ORF021542; ORF021563; ORF021731; ORF025126; ORF026134; ORF085760
NDHNSDGFIST	ORF062015	1	26.09	3.90	3.90	1	None	ORF015592
NDHNSDGFISTK	ORF062015	1	47.40	11.20	11.20	1	None	ORF015592
NDHNSDGFISTKEYNVYRHDEL	ORF062015	1	47.39	0.00	0.00	1	None	ORF015592
NDIRNTKDVDMK	ORF078612	2	35.91	7.85	14.30	2	None	ORF019339; ORF078617; ORF078626; ORF078629; ORF078634
NDIRNTKDVDMKIGV	ORF078612	4	47.23	-0.90	6.30	4	None	ORF019339; ORF078617; ORF078626; ORF078629; ORF078634
NDTTPTSGGGLR	ORF078118; ORF078133	5	47.32	4.60	10.60	5	None	ORF019298; ORF078128; ORF078131; ORF078132
NDVKPADTKKPNLT	ORF053999	10	47.46	1.00	7.10	5	None	ORF012810; ORF054010; ORF054012
NEEVTKIA	ORF062221; ORF062223; ORF062226; ORF062227; ORF062229; ORF062232; ORF062233; ORF062236	2	35.82	-0.40	0.60	2	None	ORF014920
NFESDHLKGVK	ORF097179; ORF097181	1	44.29	6.20	6.20	1	None	ORF023790
NGAALQAAYKDVVDGKT	ORF036644; ORF036647	1	42.49	0.30	0.30	1	None	ORF008344; ORF036655; ORF036666; ORF036670; ORF036673
NGAALQAAYKDVVDGKTK	ORF036644; ORF036647	2	46.13	-15.30	18.60	2	None	ORF008344; ORF036655; ORF036666; ORF036670; ORF036673
NGAALQAAYKDVVDGKTKTDWALYTYEGNSNDIR	ORF036644; ORF036647	1	46.48	-3.20	3.20	1	None	ORF008344; ORF036655; ORF036666; ORF036670; ORF036673
NGLDNKLREDL	ORF071696	1	33.51	-0.50	0.50	1	None	ORF018184
NGLDNKLREDLER	ORF071696	3	44.67	-3.40	6.90	3	None	ORF018184
NGLDNKLREDLERLK	ORF071696	9	47.09	-2.40	5.00	4	None	ORF018184
NGRKTLTTVQGIADDYDK	ORF095859	2	41.59	3.50	9.30	2	None	ORF013742; ORF023722; ORF058081; ORF058082
NGTVVEHPEYGEVIQLQG	ORF095859	1	42.56	-0.60	0.60	1	None	ORF023722

Peptide sequence	Incomplete ORF(s)	PSMs (n)	Best −10LgP	Median ppm	Max ∣ppm∣	Replicates (n)	PTM	Complete ORF match(es)
NGTVVEHPEYGEVIQLQGDQR	ORF095859	5	46.66	-1.60	3.40	5	None	ORF023722
NIDEGARQIK	ORF062221; ORF062223; ORF062226; ORF062227; ORF062229; ORF062232; ORF062233; ORF062236	9	45.26	2.80	7.40	6	None	ORF014920
NIDEGARQIKA	ORF062221; ORF062223; ORF062226; ORF062227; ORF062229; ORF062232; ORF062233; ORF062236	1	39.07	-4.80	4.80	1	None	ORF014920
NIDVAKVNTLIRPDGEK	ORF067090	1	47.02	1.50	1.50	1	None	ORF016564
NIFGSSTEPENMPR	ORF063827	2	46.08	-1.10	7.10	2	None	ORF015212
NIFTRPAGSRVEEQVPQ	ORF062221; ORF062223; ORF062226; ORF062227; ORF062229; ORF062232; ORF062233; ORF062236	4	38.59	-2.95	4.80	4	None	ORF014920
NIFTRPAGSRVEEQVPQFLILT	ORF062221; ORF062223; ORF062226; ORF062227; ORF062229; ORF062232; ORF062233; ORF062236	1	43.43	-4.50	4.50	1	None	ORF014920
NILGTISGLAGSGQAGGAGK	ORF089382	1	38.87	-9.20	9.20	1	None	ORF021680; ORF089369; ORF089372; ORF089373; ORF089377; ORF089379; ORF089383
NIVPKTDVETIVTVQ	ORF044453	3	44.76	-2.90	4.90	2	None	ORF008679
NIVPKTDVETIVTVQG	ORF044453	1	35.47	-2.00	2.00	1	None	ORF008679
NKFTGVSEDIDPDRRDVV	ORF062221; ORF062223; ORF062226; ORF062227; ORF062229; ORF062232; ORF062233; ORF062236	1	39.66	6.80	6.80	1	None	ORF014920
NKLDHYAIIK	ORF067090	1	36.33	1.30	1.30	1	None	ORF006342; ORF016564
NKLDHYAIIKFPL	ORF067090	2	46.02	-3.00	3.00	1	None	ORF006342; ORF016564
NKLDHYAIIKFPLT	ORF067090	4	44.53	-0.35	4.20	4	None	ORF006342; ORF016564
NKLDHYAIIKFPLTTESAM	ORF067090	4	45.51	-4.15	5.20	2	Oxidation (M)	ORF006342; ORF016564
NKLDHYAIIKFPLTTESAM(+15.99)	ORF067090	4	45.51	-4.15	5.20	2	Oxidation (M)	ORF006342; ORF016564
NKLDHYAIIKFPLTTESAMK	ORF067090	3	47.03	-0.40	3.90	3	None	ORF006342; ORF016564
NKQVYSTEPNNLK	ORF091776; ORF091777	8	47.32	-1.85	11.80	5	None	ORF021747; ORF022702
NKQVYSTEPNNLKA	ORF091776; ORF091777	5	47.46	-3.20	8.20	4	None	ORF021747; ORF022702
NLTDADLIQLLQNYGIK	ORF076580; ORF076584	6	47.46	1.80	6.00	4	None	ORF018429; ORF076577; ORF076579; ORF076582
NLTDADLIQLLQNYGIKHGPIVESTR	ORF076580; ORF076584	1	26.22	-5.40	5.40	1	None	ORF018429; ORF076577; ORF076579; ORF076582
NNRDIIMDAVTKAE	ORF062221; ORF062223; ORF062226; ORF062227; ORF062229; ORF062232; ORF062233; ORF062236	1	46.23	-1.10	1.10	1	None	ORF014920
NPPGFAFVEFEDPR	ORF027075; ORF027076; ORF027079	1	47.43	-0.30	0.30	1	None	ORF004248
NPPQEKTIEEQL	ORF053166	2	46.54	6.40	9.60	2	None	ORF003049; ORF053159; ORF053173; ORF053180
NPVILGTIK	ORF072898; ORF072899; ORF072900; ORF072906; ORF072907	1	30.72	3.80	3.80	1	None	ORF017928; ORF072897; ORF072901; ORF072902; ORF072903; ORF072904; ORF072905

Peptide sequence	Incomplete ORF(s)	PSMs (n)	Best −10LgP	Median ppm	Max ∣ppm∣	Replicates (n)	PTM	Complete ORF match(es)
NQFVKSAGSRIEEGVPQH	ORF062221; ORF062223; ORF062226; ORF062227; ORF062229; ORF062232; ORF062233; ORF062236	1	32.53	12.10	12.10	1	None	ORF014920
NQGGNFVVTAAGK	ORF044453	1	25.86	-10.80	10.80	1	None	ORF008679
NQYLGGGGGGGGGFGGRGFG	ORF073021	1	43.54	19.80	19.80	1	None	ORF017885
NRWEIGDVTTAA	ORF079438; ORF079444	3	38.88	-2.50	2.50	2	None	ORF020265; ORF079440
NSVDQVFVNVQ	ORF069950	3	32.81	-0.50	1.30	3	None	ORF016799; ORF069954; ORF069958; ORF069960
NSVDQVFVNVQK	ORF069950	6	47.41	-0.20	9.00	6	None	ORF016799; ORF069954; ORF069958; ORF069960
NTDLSRILK	ORF050709	1	31.82	1.20	1.20	1	None	ORF009765; ORF010196; ORF037399; ORF037400
NTDLSRILKSQEIQK	ORF050709	1	26.55	-4.80	4.80	1	None	ORF009765; ORF010196; ORF037399; ORF037400
NTDLSRILKSQEIQKA	ORF050709	1	42.78	2.30	2.30	1	None	ORF009765; ORF010196; ORF037399; ORF037400
NTKDVDM(+15.99)KIGV	ORF078612	8	46.45	-2.55	17.50	6	Oxidation (M)	ORF019339; ORF078617; ORF078626; ORF078629; ORF078634
NTKDVDMKIGV	ORF078612	8	46.45	-2.55	17.50	6	Oxidation (M)	ORF019339; ORF078617; ORF078626; ORF078629; ORF078634
NTLIRPDGEK	ORF067090	1	39.29	13.60	13.60	1	None	ORF006342; ORF016564
NVDSEEANKLT	ORF062221; ORF062223; ORF062226; ORF062227; ORF062229; ORF062232; ORF062233; ORF062236	7	47.42	4.50	6.90	6	None	ORF014920
NVKELEVLMMSNK	ORF066964	1	27.17	-1.40	1.40	1	None	ORF010381; ORF016315
NVKGPVREGDVLT	ORF041048	5	46.25	-2.50	4.70	4	None	ORF003580
NVKGPVREGDVLTLLE	ORF041048	5	38.05	-0.30	2.80	4	None	ORF003580
NVKGPVREGDVLTLLESE	ORF041048	4	40.33	-1.20	3.60	3	None	ORF003580
NVKGPVREGDVLTLLESER	ORF041048	10	46.98	-4.95	15.10	4	None	ORF003580
NVKGPVREGDVLTLLESEREA	ORF041048	30	47.45	-0.10	5.10	6	None	ORF003580
NVKGPVREGDVLTLLESEREAR	ORF041048	7	47.30	0.10	4.70	3	None	ORF003580
NVKGPVREGDVLTLLESEREARR	ORF041048	4	44.22	-0.35	1.90	3	None	ORF003580
NVQEELDRM(+15.99)TRQPDTVLPNSTPTENEA	ORF081161	6	46.63	-3.25	6.20	5	Oxidation (M)	ORF019667
NVQEELDRMTRQPDTVLPNSTPTENEA	ORF081161	6	46.63	-3.25	6.20	5	Oxidation (M)	ORF019667
NVRSMPIRKDDEVQVVR	ORF040982	1	45.73	5.70	5.70	1	None	ORF008943
PAADGKGVIVV	ORF091776; ORF091777	1	42.92	4.40	4.40	1	None	ORF021747; ORF022702
PAGSRVEEQVPQ	ORF062221; ORF062223; ORF062226; ORF062227; ORF062229; ORF062232; ORF062233; ORF062236	3	43.01	-0.80	12.80	3	None	ORF014920
PGAPAPGQPTDPK	ORF100266; ORF100267; ORF100268	1	44.45	-19.40	19.40	1	None	ORF024560
PGAPAPGQPTDPKVAG	ORF100266; ORF100267; ORF100268	6	47.15	-6.90	13.10	5	None	ORF024560
PGPQYPGAPAPGQPTDPKVAG	ORF100266; ORF100267; ORF100268	1	40.89	-12.00	12.00	1	None	ORF024560

Peptide sequence	Incomplete ORF(s)	PSMs (n)	Best −10LgP	Median ppm	Max ∣ppm∣	Replicates (n)	PTM	Complete ORF match(es)
PHWPARIDEILEPAA	ORF101777; ORF101779; ORF101782; ORF101784	3	33.06	-1.80	7.80	3	None	ORF025356
PIRKDDEVQVV	ORF040982	3	37.47	0.70	4.50	2	None	ORF008943
PIRKDDEVQVVR	ORF040982	11	47.16	0.60	3.00	6	None	ORF008943
PLAKDLL	ORF102745; ORF102746	4	28.35	-2.35	6.90	4	None	ORF000815; ORF008634; ORF024572
PLAKDLLHP	ORF102745; ORF102746	1	33.32	5.50	5.50	1	None	ORF000815; ORF008634; ORF024572
PLAKDLLHPSPEEE	ORF102745; ORF102746	1	27.59	5.20	5.20	1	None	ORF024572
PLAKDLLHPSPEEEK	ORF102745; ORF102746	22	47.46	1.85	10.20	6	None	ORF024572
PLAKDLLHPSPEEEKR	ORF102745; ORF102746	13	47.44	1.70	4.00	6	None	ORF024572
PLAKDLLHPSPEEEKRK	ORF102745; ORF102746	1	47.25	0.40	0.40	1	None	ORF024572
PLAKDLLHPSPEEEKRKH	ORF102745; ORF102746	1	27.84	1.20	1.20	1	None	ORF024572
PNAPDETNVVR	ORF087621; ORF087623	1	29.66	12.10	12.10	1	None	ORF021436
PRKIEEIKD	ORF098458	2	25.61	3.40	5.70	2	None	ORF023230
PRKIEEIKDF	ORF098458	3	41.66	-1.30	2.40	3	None	ORF023230
PRKIEEIKDFL	ORF098458	1	32.22	2.80	2.80	1	None	ORF023230
PRKIEEIKDFLL	ORF098458	1	29.53	2.20	2.20	1	None	ORF023230
PRKIEEIKDFLLT	ORF098458	5	43.96	1.00	2.20	4	None	ORF023230
PSAAASAGSSKVPTSQERT	ORF083445	1	40.14	-11.10	11.10	1	None	ORF001845
PSAAASAGSSKVPTSQERTM	ORF083445	1	38.68	-6.90	6.90	1	None	ORF001845
PVPGPAPIPDLK	ORF095365	1	32.62	-1.80	1.80	1	None	ORF024066; ORF095359; ORF095363; ORF095366
PVREGDVLTLLESEREA	ORF041048	1	47.02	-1.60	1.60	1	None	ORF003580
QADFLEGHSEQATQHVSG	ORF060338; ORF060364	1	35.78	13.00	13.00	1	None	ORF002404; ORF060337; ORF060344; ORF060345; ORF060349; ORF060350; ORF060353; ORF060354; ORF060356
QADFLEGHSEQATQHVSGLQ	ORF060338; ORF060364	1	47.02	2.10	2.10	1	None	ORF002404; ORF060337; ORF060344; ORF060345; ORF060349; ORF060350; ORF060353; ORF060354; ORF060356
QATQHVSGLQ	ORF060338; ORF060364	1	29.36	-2.50	2.50	1	None	ORF002404; ORF060337; ORF060344; ORF060345; ORF060349; ORF060350; ORF060353; ORF060354; ORF060356
QEITENETLTPELK	ORF061770	1	36.10	5.80	5.80	1	None	ORF015690; ORF061765; ORF061767
QELGPPYHDPFSDPYVPI	ORF080397	1	35.93	-0.10	0.10	1	None	ORF019503
QESPITPLIAPPRPK	ORF027357	3	46.58	-3.50	4.30	3	None	ORF001771
QGGVLPNIQAVLLPKK	ORF067172	1	46.46	0.30	0.30	1	None	ORF016539; ORF020361; ORF020365; ORF020604; ORF020626; ORF020632; ORF020636; ORF020638; ORF020641; ORF020717; ORF020720; ORF020908; ORF020913; ORF020951; ORF020954; ORF021008; ORF021012; ORF021015; ORF021020; ORF021386; ORF021497; ORF021500; ORF021528; ORF021529; ORF021532; ORF021534; ORF021542; ORF021563; ORF021731; ORF085760
QIGKVVQV	ORF040982	2	31.04	-3.05	5.80	2	None	ORF008943; ORF022249
QILMPNIGYGSNKK	ORF066964	1	40.99	2.00	2.00	1	None	ORF005131; ORF012718; ORF016315
QISKPTPEAPADVDNAADLLSQ	ORF055592	1	31.84	-1.30	1.30	1	None	ORF013351

Peptide sequence	Incomplete ORF(s)	PSMs (n)	Best −10LgP	Median ppm	Max ∣ppm∣	Replicates (n)	PTM	Complete ORF match(es)
QISSGGSSSGGGAANPPR	ORF072898; ORF072899; ORF072900; ORF072906; ORF072907	6	47.45	-1.90	10.40	4	None	ORF017928; ORF072897; ORF072901; ORF072902; ORF072903; ORF072904; ORF072905
QNDIRNTKDVDMKIGV	ORF078612	2	47.21	-0.70	1.70	2	None	ORF019339; ORF078617; ORF078626; ORF078629; ORF078634
QNGDTVKSQEMVQLVS	ORF078612	2	28.41	3.40	5.40	2	None	ORF019339; ORF078617; ORF078626; ORF078629; ORF078634
QPRPATAPDM(+15.99)KNIR	ORF053999	11	47.43	3.30	11.60	4	Oxidation (M)	ORF012810; ORF054010; ORF054012
QPRPATAPDMKNIR	ORF053999	11	47.43	3.30	11.60	4	Oxidation (M)	ORF012810; ORF054010; ORF054012
QPSLELLSPTDPVSNIK	ORF079438; ORF079444	3	35.58	0.10	2.60	3	None	ORF020265; ORF079440
QRVVEGLDVDKDKIR	ORF062221; ORF062223; ORF062226; ORF062227; ORF062229; ORF062232; ORF062233; ORF062236	2	46.57	-3.80	5.10	2	None	ORF014920
QTGELGDQDEPLTQNDIRNTKDVDMKIGV	ORF078612	1	33.92	-3.50	3.50	1	None	ORF019339; ORF078617; ORF078626; ORF078629; ORF078634
QTGVPDKVKDIYDQAAEK	ORF090968	4	47.46	-0.05	2.10	4	None	ORF021892
QTGVPDKVKDIYDQAAEKVS	ORF090968	1	36.06	0.60	0.60	1	None	ORF021892
QTPAVKPKDSEVKKAEPEAVISASSSSSVQ	ORF083443	5	47.28	4.50	4.70	4	None	ORF001845
QVFVNVQKIFDREG	ORF069950	1	46.63	-2.90	2.90	1	None	ORF016799; ORF069954; ORF069958; ORF069960
QVRVEFMDDTNRSIIR	ORF041048	1	41.14	-2.20	2.20	1	None	ORF003580
QVRVEFMDDTNRSIIRNVK	ORF041048	2	44.85	-3.80	4.10	2	None	ORF003580
QVYSTEPNNLK	ORF091776; ORF091777	2	26.70	8.20	12.30	2	None	ORF021747; ORF022702
QYPGAPAPGQPTDPK	ORF100266; ORF100267; ORF100268	5	46.83	-0.90	8.40	5	None	ORF024560
QYPGAPAPGQPTDPKVA	ORF100266; ORF100267; ORF100268	2	46.39	-16.30	18.80	2	None	ORF024560
QYPGAPAPGQPTDPKVAG	ORF100266; ORF100267; ORF100268	11	47.46	3.90	7.10	6	None	ORF024560
QYPGAPAPGQPTDPKVAGQSGP	ORF100266; ORF100267; ORF100268	6	47.14	-2.45	8.90	4	None	ORF024560
RAGELTEDEVERVVT	ORF071696	2	47.24	-5.00	6.10	2	None	ORF018184
RAGELTEDEVERVVTIM	ORF071696	2	46.77	-2.90	3.00	2	None	ORF018184
RAGELTEDEVERVVTIMQNPR	ORF071696	10	47.46	-1.50	8.10	5	None	ORF018184
RAGELTEDEVERVVTIMQNPRQ	ORF071696	3	46.94	-4.10	4.10	2	None	ORF018184
RDQKLSELDDRADALQ	ORF044627	2	36.27	-0.80	3.90	2	None	ORF009505
REGDVLTLLESER	ORF041048	14	44.40	-1.30	5.20	6	None	ORF003580
REGDVLTLLESEREA	ORF041048	16	45.75	-2.60	5.30	6	None	ORF003580
REGDVLTLLESEREAR	ORF041048	3	46.66	0.30	0.80	3	None	ORF003580
REGDVLTLLESEREARR	ORF041048	9	43.90	-1.50	3.80	6	None	ORF003580
REGDVLTLLESEREARRL	ORF041048	2	43.89	-3.20	6.30	2	None	ORF003580
RGAGGQGGGGAASNI	ORF089382	1	40.72	-2.70	2.70	1	None	ORF021680; ORF089369; ORF089372; ORF089373; ORF089377; ORF089379; ORF089383
RGETSGRVDDNEETIK	ORF076196	1	32.44	-3.30	3.30	1	None	ORF018471; ORF076197; ORF076198
RGSTDTIVKPLLVQPGGV	ORF087621; ORF087623	2	39.65	-7.20	10.10	2	None	ORF021436

Peptide sequence	Incomplete ORF(s)	PSMs (n)	Best −10LgP	Median ppm	Max ∣ppm∣	Replicates (n)	PTM	Complete ORF match(es)
RGSTDTIVKPLLVQPGGVLVE	ORF087621; ORF087623	2	40.56	-0.55	1.90	2	None	ORF021436
RIDEILEPAA	ORF101777; ORF101779; ORF101782; ORF101784	4	27.59	-1.00	3.20	4	None	ORF025356
RIEEGVPQH	ORF062221; ORF062223; ORF062226; ORF062227; ORF062229; ORF062232; ORF062233; ORF062236	2	40.26	4.25	6.80	2	None	ORF014920
RIEEGVPQHLVLLTGGKSVDDVRGAA	ORF062221; ORF062223; ORF062226; ORF062227; ORF062229; ORF062232; ORF062233; ORF062236	1	47.44	-3.00	3.00	1	None	ORF014920
RKDDEVQVVR	ORF040982	6	40.86	1.35	4.10	5	None	ORF008943
RKGFSEGLWEIENNPTVK	ORF101777; ORF101779; ORF101782; ORF101784	5	46.33	-2.50	3.50	4	None	ORF025356
RNDEELNKLLGGVT	ORF067172	1	43.60	-5.40	5.40	1	None	ORF016539; ORF020361; ORF020365; ORF020604; ORF020632; ORF020636; ORF020638; ORF020641; ORF020717; ORF020720; ORF020908; ORF020913; ORF020951; ORF020954; ORF021008; ORF021012; ORF021015; ORF021020; ORF021386; ORF021497; ORF021500; ORF021528; ORF021529; ORF021532; ORF021534; ORF021537; ORF021542; ORF021563; ORF021731; ORF025126; ORF026134; ORF085760
RPAARASPVAPKL	ORF053999	2	26.83	-11.75	15.00	2	None	ORF012810; ORF054010; ORF054012
RPIIDLYEK	ORF069950	4	40.74	0.65	7.60	4	None	ORF016799; ORF069954; ORF069958; ORF069960
RQPDTVLPNSTPTENEA	ORF081161	1	42.88	4.80	4.80	1	None	ORF019667
RQPSLELLSPTDPVSNIK	ORF079438; ORF079444	10	46.96	-3.40	4.20	6	None	ORF020265; ORF079440
RSEPPLPIHKIDDRLE	ORF079438; ORF079444	6	46.95	-0.25	4.70	6	None	ORF020265; ORF079440
RTEIETIVTDPR	ORF062221; ORF062223; ORF062226; ORF062227; ORF062229; ORF062232; ORF062233; ORF062236	4	43.40	-0.20	5.10	4	None	ORF014920
RVEPSQGSVGAVTKPVSK	ORF051130; ORF051137	1	47.38	-1.40	1.40	1	None	ORF012252
RVPNAPDETNVVR	ORF087621; ORF087623	1	47.13	-3.40	3.40	1	None	ORF021436
RVSELKTDDIEILR	ORF062221; ORF062223; ORF062226; ORF062227; ORF062229; ORF062232; ORF062233; ORF062236	1	44.41	-1.40	1.40	1	None	ORF014920
RVVEGLDVDKDK	ORF062221; ORF062223; ORF062226; ORF062227; ORF062229; ORF062232; ORF062233; ORF062236	1	46.76	0.80	0.80	1	None	ORF014920
RVVEGLDVDKDKIR	ORF062221; ORF062223; ORF062226; ORF062227; ORF062229; ORF062232; ORF062233; ORF062236	1	47.15	-3.10	3.10	1	None	ORF014920
RVVEGLDVDKDKIRIG	ORF062221; ORF062223; ORF062226; ORF062227; ORF062229; ORF062232; ORF062233; ORF062236	1	46.78	0.40	0.40	1	None	ORF014920
SAAGGTAPKPNVIT	ORF053999	1	43.04	-16.00	16.00	1	None	ORF012810; ORF054010; ORF054012
SAAGGTAPKPNVITK	ORF053999	3	46.65	-2.90	4.10	2	None	ORF012810; ORF054010; ORF054012
SAAPVEANVAQPK	ORF027357	5	47.46	-0.60	9.00	5	None	ORF001771

Peptide sequence	Incomplete ORF(s)	PSMs (n)	Best −10LgP	Median ppm	Max ∣ppm∣	Replicates (n)	PTM	Complete ORF match(es)
SAGSRIEEGVPQH	ORF062221; ORF062223; ORF062226; ORF062227; ORF062229; ORF062232; ORF062233; ORF062236	10	46.75	0.35	8.10	6	None	ORF014920
SAGSRIEEGVPQHL	ORF062221; ORF062223; ORF062226; ORF062227; ORF062229; ORF062232; ORF062233; ORF062236	2	42.36	3.20	5.90	2	None	ORF014920
SAGSRIEEGVPQHLV	ORF062221; ORF062223; ORF062226; ORF062227; ORF062229; ORF062232; ORF062233; ORF062236	4	45.94	-1.85	9.50	3	None	ORF014920
SAGSRIEEGVPQHLVL	ORF062221; ORF062223; ORF062226; ORF062227; ORF062229; ORF062232; ORF062233; ORF062236	8	46.39	-1.30	5.80	6	None	ORF014920
SAGSRIEEGVPQHLVLLT	ORF062221; ORF062223; ORF062226; ORF062227; ORF062229; ORF062232; ORF062233; ORF062236	3	44.84	-5.50	5.90	3	None	ORF014920
SAGSRIEEGVPQHLVLLTGGK SVDDVR	ORF062221; ORF062223; ORF062226; ORF062227; ORF062229; ORF062232; ORF062233; ORF062236	5	47.15	1.80	5.50	4	None	ORF014920
SAGSRIEEGVPQHLVLLTGGK SVDDVRGAA	ORF062221; ORF062223; ORF062226; ORF062227; ORF062229; ORF062232; ORF062233; ORF062236	10	47.46	0.40	3.40	5	None	ORF014920
SAHPLPKTTEKF	ORF032526	12	46.66	0.60	5.10	6	None	ORF004880; ORF005253
SAIPEIYRTDTQVVQLD	ORF061770	1	32.25	6.50	6.50	1	None	ORF015690; ORF061765; ORF061767
SAPPRDVVIDVEDTKPL	ORF087579	3	36.98	-3.90	7.40	3	None	ORF021453
SAPPRDVVIDVEDTKPLL	ORF087579	3	44.87	-2.20	2.60	3	None	ORF021453
SAPSAAASAGSSKVPTSQ	ORF083445	4	47.46	-4.20	6.20	4	None	ORF001845
SAPSAAASAGSSKVPTSQE	ORF083445	3	47.25	-6.60	6.60	3	None	ORF001845
SAPSAAASAGSSKVPTSQER	ORF083445	6	47.14	0.50	4.30	3	None	ORF001845
SAPSAAASAGSSKVPTSQERT	ORF083445	1	46.63	-1.70	1.70	1	None	ORF001845
SAPSYDGSADAAALDKAIKT	ORF060338; ORF060339; ORF060340; ORF060352; ORF060355; ORF060357; ORF060358; ORF060364; ORF060367; ORF060370; ORF060374; ORF060375	1	39.65	-3.70	3.70	1	None	ORF000781; ORF002404; ORF060337; ORF060344; ORF060345; ORF060349; ORF060350; ORF060353; ORF060354; ORF060356
SASADAKKVPTTRPAPL	ORF053999	1	47.13	-2.20	2.20	1	None	ORF012810; ORF054010; ORF054012
SAYSSITSVADGLYDK	ORF090968	2	44.94	3.75	4.80	2	None	ORF021892
SDDLGLTEPPLK	ORF087796; ORF087805; ORF087831	5	41.87	-1.30	10.80	5	None	ORF021301; ORF087775; ORF087778; ORF087823
SDGYGNNPPYSGNR	ORF031536; ORF031542	8	47.44	3.95	5.20	6	None	ORF004221
SDNIDEGARQIK	ORF062221; ORF062223; ORF062226; ORF062227; ORF062229; ORF062232; ORF062233; ORF062236	11	46.41	1.70	5.40	6	None	ORF014920
SDQLYHWTGSD	ORF090968	3	47.44	6.00	6.30	3	None	ORF021892
SDQTNQGGNFVVT	ORF044453	3	41.01	1.10	3.50	3	None	ORF008679
SDQTNQGGNFVVTAAGK	ORF044453	9	47.46	5.00	5.20	4	None	ORF008679

Peptide sequence	Incomplete ORF(s)	PSMs (n)	Best −10LgP	Median ppm	Max |ppm|	Replicates (n)	PTM	Complete ORF match(es)
SDQTNQGGNFVVTAAGKGE GTLS	ORF044453	2	46.66	-3.00	3.10	2	None	ORF008679
SDTTRPNFQ	ORF062221; ORF062223; ORF062226; ORF062227; ORF062229; ORF062232; ORF062233; ORF062236	2	46.61	3.55	4.40	2	None	ORF014920
SEALTYMTKGYQR	ORF087614	1	33.04	3.10	3.10	1	None	ORF021439
SEAPSAFRVPTVQ	ORF062221; ORF062223; ORF062226; ORF062227; ORF062229; ORF062232; ORF062233; ORF062236	2	46.98	2.90	4.90	2	None	ORF014920
SEAPSAFRVPTVQALS	ORF062221; ORF062223; ORF062226; ORF062227; ORF062229; ORF062232; ORF062233; ORF062236	1	47.11	-7.80	7.80	1	None	ORF014920
SEASKVSEPTTEAKVSQPSE	ORF057182	6	46.65	5.70	7.60	4	None	ORF012631; ORF057183
SEDPKVEF	ORF062221; ORF062223; ORF062226; ORF062227; ORF062229; ORF062232; ORF062233; ORF062236	6	45.93	1.50	6.20	5	None	ORF014920
SEDPKVEFL	ORF062221; ORF062223; ORF062226; ORF062227; ORF062229; ORF062232; ORF062233; ORF062236	3	33.38	1.40	1.80	3	None	ORF014920
SEEPEQLR	ORF031536	7	42.91	3.70	6.30	6	None	ORF004221
SEGLWEIENNPTVK	ORF101777; ORF101779; ORF101782; ORF101784	13	47.18	-1.10	4.80	6	None	ORF025356
SEGLWEIENNPTVKA	ORF101777; ORF101779; ORF101782; ORF101784	2	44.25	-5.00	5.00	1	None	ORF025356
SEKLDFKERIQ	ORF095365	1	44.06	3.90	3.90	1	None	ORF024066; ORF095359; ORF095363; ORF095366
SEKTEASSEAKAEPQS	ORF057182	5	47.05	-6.90	9.80	4	None	ORF012631; ORF057183
SELKTDDIEI	ORF062221; ORF062223; ORF062226; ORF062227; ORF062229; ORF062232; ORF062233; ORF062236	2	27.67	5.30	10.10	2	None	ORF014920
SELKTDDIEILR	ORF062221; ORF062223; ORF062226; ORF062227; ORF062229; ORF062232; ORF062233; ORF062236	7	46.10	1.80	4.80	6	None	ORF014920
SEPPLPIHK	ORF079438; ORF079444	5	32.92	0.70	2.50	4	None	ORF020265; ORF079440
SEPPLPIHKIDDRLE	ORF079438; ORF079444	18	47.02	-1.80	9.70	6	None	ORF020265; ORF079440
SEPPLPIHKIDDRLEQ	ORF079438; ORF079444	1	39.37	-4.70	4.70	1	None	ORF020265; ORF079440
SEQATQHVSGLQ	ORF060338; ORF060364	3	45.94	1.10	6.20	3	None	ORF002404; ORF060337; ORF060344; ORF060345; ORF060349; ORF060350; ORF060353; ORF060354; ORF060356
SFETTDESLR	ORF031536; ORF031542	3	38.21	-2.60	11.60	3	None	ORF004221
SFIQRVVEGLDVDKD	ORF062221; ORF062223; ORF062226; ORF062227; ORF062229; ORF062232; ORF062233; ORF062236	1	38.49	0.20	0.20	1	None	ORF014920

Peptide sequence	Incomplete ORF(s)	PSMs (n)	Best −10LgP	Median ppm	Max |ppm|	Replicates (n)	PTM	Complete ORF match(es)
SFIQRVVEGLDVDKDK	ORF062221; ORF062223; ORF062226; ORF062227; ORF062229; ORF062232; ORF062233; ORF062236	7	47.33	0.20	5.20	6	None	ORF014920
SFIQRVVEGLDVDKDKIR	ORF062221; ORF062223; ORF062226; ORF062227; ORF062229; ORF062232; ORF062233; ORF062236	9	47.45	-1.30	3.30	6	None	ORF014920
SFSDIEKHAQAVDK	ORF043012	2	45.99	-3.80	11.20	2	None	ORF007631; ORF043008
SGADRTEIETIVTD	ORF062221; ORF062223; ORF062226; ORF062227; ORF062229; ORF062232; ORF062233; ORF062236	1	25.95	11.30	11.30	1	None	ORF014920
SGADRTEIETIVTDP	ORF062221; ORF062223; ORF062226; ORF062227; ORF062229; ORF062232; ORF062233; ORF062236	2	26.09	-0.70	0.70	1	None	ORF014920
SGADRTEIETIVTDPR	ORF062221; ORF062223; ORF062226; ORF062227; ORF062229; ORF062232; ORF062233; ORF062236	17	46.87	0.80	2.20	6	None	ORF014920
SGADRTEIETIVTDPRY	ORF062221; ORF062223; ORF062226; ORF062227; ORF062229; ORF062232; ORF062233; ORF062236	2	46.00	1.00	2.00	2	None	ORF014920
SGADRTEIETIVTDPRYL	ORF062221; ORF062223; ORF062226; ORF062227; ORF062229; ORF062232; ORF062233; ORF062236	5	43.35	-2.90	5.30	5	None	ORF014920
SGERSGYSSPGSPSTPTGR	ORF095365	2	44.31	4.55	11.20	2	None	ORF024066; ORF095359; ORF095363; ORF095366
SGFGFVEFEDHRDA	ORF100927	2	34.92	-9.30	9.30	1	None	ORF025409; ORF100925; ORF100933
SGFPGYPGYPAGDPAYAPGGQQ	ORF086771	8	47.09	2.80	10.10	6	None	ORF020965
SGGEVLPSKVIDYGHSS	ORF085019	7	47.25	-1.50	6.30	5	None	ORF001870; ORF085013
SGGEVLPSKVIDYGHSSK	ORF085019	2	47.42	4.80	6.40	2	None	ORF001870; ORF085013
SGGGGGGSGFGGGNGNFGGGSGGGGGSN	ORF031536; ORF031542	2	47.46	-4.35	7.30	2	None	ORF004221
SGGGGGGSGFGGGNGNFGGGSGGGGGSNFN	ORF031536; ORF031542	1	47.27	-3.00	3.00	1	None	ORF004221
SGGGGGISLQNPV	ORF072898; ORF072899; ORF072900; ORF072906; ORF072907	1	38.12	10.80	10.80	1	None	ORF017928; ORF072897; ORF072901; ORF072902; ORF072903; ORF072904; ORF072905
SGIDDIKTLEEKLR	ORF043114	1	34.02	-5.20	5.20	1	None	ORF007666; ORF043103; ORF043107; ORF043110
SGKQVPQEQEASVSLEK	ORF087796; ORF087805; ORF087831	1	47.38	3.40	3.40	1	None	ORF021301; ORF087775; ORF087778; ORF087823
SGPSSDNIDEGARQIK	ORF062221; ORF062223; ORF062226; ORF062227; ORF062229; ORF062232; ORF062233; ORF062236	6	47.04	-1.25	5.20	3	None	ORF014920
SGQAGGAGKFIK	ORF089382	4	47.38	2.80	5.40	4	None	ORF021680; ORF089369; ORF089372; ORF089373; ORF089377; ORF089379; ORF089383
SGQGGGGGGGVNVGNLIGGLS	ORF089382	1	43.71	1.30	1.30	1	None	ORF021680; ORF089369; ORF089372; ORF089373; ORF089377; ORF089379; ORF089383
SGQSRGAGGQGGGGAASNI	ORF089382	5	47.45	6.30	14.80	5	None	ORF021680; ORF089369; ORF089372; ORF089373; ORF089377; ORF089379; ORF089383

Peptide sequence	Incomplete ORF(s)	PSMs (n)	Best −10LgP	Median ppm	Max ∣ppm∣	Replicates (n)	PTM	Complete ORF match(es)
SGSEEIVVYSK	ORF083443	4	47.18	2.85	5.90	4	None	ORF001845
SGSEEIVVYSKVS	ORF083443	2	42.42	-14.80	17.00	2	None	ORF001845
SGSEEIVVYSKVSSVQ	ORF083443	6	47.44	-1.15	17.10	5	None	ORF001845
SGSEEIVVYSKVSSVQS	ORF083443	4	46.44	-1.40	6.10	2	None	ORF001845
SGSTRIRLNNQNALNA	ORF044453	2	31.81	-0.70	3.10	2	None	ORF008679
SIGIGTISNEEVTK	ORF062221; ORF062223; ORF062226; ORF062227; ORF062229; ORF062232; ORF062233; ORF062236	10	47.15	0.40	6.20	6	None	ORF014920
SIGIGTISNEEVTKIA	ORF062221; ORF062223; ORF062226; ORF062227; ORF062229; ORF062232; ORF062233; ORF062236	10	47.15	2.05	2.50	5	None	ORF014920
SIGIGTISNEEVTKIAS	ORF062221; ORF062223; ORF062226; ORF062227; ORF062229; ORF062232; ORF062233; ORF062236	2	47.15	1.55	1.90	2	None	ORF014920
SIGIGTISNEEVTKIASEAPSA	ORF062221; ORF062223; ORF062226; ORF062227; ORF062229; ORF062232; ORF062233; ORF062236	3	47.02	-7.30	8.90	3	None	ORF014920
SIGIGTISNEEVTKIASEAPSAF R	ORF062221; ORF062223; ORF062226; ORF062227; ORF062229; ORF062232; ORF062233; ORF062236	1	47.15	-4.40	4.40	1	None	ORF014920
SIGIGTISNEEVTKIASEAPSAF RVPTVQ	ORF062221; ORF062223; ORF062226; ORF062227; ORF062229; ORF062232; ORF062233; ORF062236	1	26.76	-0.40	0.40	1	None	ORF014920
SIIRNVKGPVREGDVLTLLESE REA	ORF041048	3	45.13	-8.90	8.90	2	None	ORF003580
SIPKEEVKTE	ORF083445; ORF083446	2	45.29	0.40	2.70	2	None	ORF001845
SIPKEEVKTEK	ORF083445; ORF083446	3	46.76	4.80	7.30	3	None	ORF001845
SIVPFLIGKNVDSEEANK	ORF062221; ORF062223; ORF062226; ORF062227; ORF062229; ORF062232; ORF062233; ORF062236	2	46.33	-5.60	5.80	2	None	ORF014920
SIYTGVLSDQLYHWTGSD	ORF090968	3	46.57	2.20	3.40	3	None	ORF021892
SKEQTGELGDQDEPLTQNDIR NTKDVDM(+15.99)K	ORF078612	2	47.26	-3.35	7.70	2	Oxidation (M)	ORF019339; ORF078617; ORF078626; ORF078629; ORF078634
SKEQTGELGDQDEPLTQNDIR NTKDVDM(+15.99)KIGV	ORF078612	11	47.26	2.00	3.10	5	Oxidation (M)	ORF019339; ORF078617; ORF078626; ORF078629; ORF078634
SKEQTGELGDQDEPLTQNDIR NTKDVDMK	ORF078612	2	47.26	-3.35	7.70	2	Oxidation (M)	ORF019339; ORF078617; ORF078626; ORF078629; ORF078634
SKEQTGELGDQDEPLTQNDIR NTKDVDMKIGV	ORF078612	11	47.26	2.00	3.10	5	Oxidation (M)	ORF019339; ORF078617; ORF078626; ORF078629; ORF078634
SKGDDWLPAGTEDSIHI	ORF095859	1	40.86	-2.20	2.20	1	None	ORF023722
SKGDDWLPAGTEDSIHIR	ORF095859	4	46.97	-1.05	6.80	4	None	ORF023722
SKIGSTDNLK	ORF053999	2	36.92	6.90	7.30	2	None	ORF012810; ORF054001; ORF054006; ORF054010; ORF054012; ORF054013; ORF054018

Peptide sequence	Incomplete ORF(s)	PSMs (n)	Best −10LgP	Median ppm	Max ∣ppm∣	Replicates (n)	PTM	Complete ORF match(es)
SLAGSGQGGGGGGGVNVGNLIGGLS	ORF089382	2	44.47	-4.40	8.00	2	None	ORF021680; ORF089369; ORF089372; ORF089373; ORF089377; ORF089379; ORF089383
SLAGSGQSRGAGGQGGGGAASN	ORF089382	2	47.46	-4.25	9.40	2	None	ORF021680; ORF089369; ORF089372; ORF089373; ORF089377; ORF089379; ORF089383
SLAGSGQSRGAGGQGGGGAASNI	ORF089382	1	45.19	-9.90	9.90	1	None	ORF021680; ORF089369; ORF089372; ORF089373; ORF089377; ORF089379; ORF089383
SLAVATSGADRTEIETIVTDPR	ORF062221; ORF062223; ORF062226; ORF062227; ORF062229; ORF062232; ORF062233; ORF062236	11	47.46	0.50	5.50	6	None	ORF014920
SLAVATSGADRTEIETIVTDPRY	ORF062221; ORF062223; ORF062226; ORF062227; ORF062229; ORF062232; ORF062233; ORF062236	2	46.65	4.50	4.50	1	None	ORF014920
SLAVATSGADRTEIETIVTDPRYL	ORF062221; ORF062223; ORF062226; ORF062227; ORF062229; ORF062232; ORF062233; ORF062236	6	47.15	-3.00	6.20	5	None	ORF014920
SLELLSPTDPVSNIK	ORF079438; ORF079444	4	43.85	2.50	4.80	4	None	ORF020265; ORF079440
SLVTAEIEAHEPVT	ORF079333	1	41.27	-3.70	3.70	1	None	ORF020230
SM(+15.99)PIRKDDEVQVVR	ORF040982	13	47.40	1.20	9.50	6	Oxidation (M)	ORF008943
SMPIRKDDEVQVVR	ORF040982	13	47.40	1.20	9.50	6	Oxidation (M)	ORF008943
SNDPASLFYLK	ORF062221; ORF062223; ORF062226; ORF062227; ORF062229; ORF062232; ORF062233; ORF062236	1	25.78	2.30	2.30	1	None	ORF014920
SNIFGSSTEPENMPR	ORF063827	4	45.87	6.55	15.30	4	None	ORF015212
SNSVDQVFVNVQ	ORF069950	4	44.37	1.00	6.40	4	None	ORF016799; ORF069954; ORF069958; ORF069960
SNSVDQVFVNVQK	ORF069950	6	47.38	0.00	6.70	5	None	ORF016799; ORF069954; ORF069958; ORF069960
SNSVDQVFVNVQKIFDREG	ORF069950	2	45.26	-3.35	4.90	2	None	ORF016799; ORF069954; ORF069958; ORF069960
SPAPPEEKKPKLE	ORF083445; ORF083446	2	35.25	-0.85	1.10	2	None	ORF001845
SPFVTSDRSKNR	ORF040982	1	38.56	-0.30	0.30	1	None	ORF008943; ORF022249
SPNSYFMDVK	ORF102745; ORF102746	3	40.72	5.20	5.30	3	None	ORF008634; ORF024572
SPPLPPVKPT	ORF029551; ORF029554; ORF029560; ORF029561; ORF029562	1	35.52	-4.20	4.20	1	None	ORF005045; ORF029552; ORF029553; ORF029555; ORF029563; ORF029564; ORF029566
SRGAGGQGGGGAASNI	ORF089382	1	43.54	-4.10	4.10	1	None	ORF021680; ORF089369; ORF089372; ORF089373; ORF089377; ORF089379; ORF089383
SRIEEGVPQH	ORF062221; ORF062223; ORF062226; ORF062227; ORF062229; ORF062232; ORF062233; ORF062236	2	43.24	2.80	5.40	2	None	ORF014920
SRIEEGVPQHLVLLTGGKSVDDVR	ORF062221; ORF062223; ORF062226; ORF062227; ORF062229; ORF062232; ORF062233; ORF062236	4	46.47	-1.30	3.90	4	None	ORF014920
SRIEEGVPQHLVLLTGGKSVDDVRGAA	ORF062221; ORF062223; ORF062226; ORF062227; ORF062229; ORF062232; ORF062233; ORF062236	4	46.66	2.15	2.60	4	None	ORF014920
SRLQPVPGPAPIPDLK	ORF095365	2	39.62	-2.45	2.80	2	None	ORF024066; ORF095359; ORF095363; ORF095366

Peptide sequence	Incomplete ORF(s)	PSMs (n)	Best −10LgP	Median ppm	Max |ppm|	Replicates (n)	PTM	Complete ORF match(es)
SSAAGGTAPKPNVITK	ORF053999	1	47.46	3.70	3.70	1	None	ORF012810; ORF054010; ORF054012
SSAYSSITSVADGLYDK	ORF090968	2	44.08	3.25	4.50	2	None	ORF021892
SSDNIDEGARQI	ORF062221; ORF062223; ORF062226; ORF062227; ORF062229; ORF062232; ORF062233; ORF062236	1	33.45	-5.00	5.00	1	None	ORF014920
SSDNIDEGARQIK	ORF062221; ORF062223; ORF062226; ORF062227; ORF062229; ORF062232; ORF062233; ORF062236	17	47.45	2.60	5.10	6	None	ORF014920
SSDNIDEGARQIKA	ORF062221; ORF062223; ORF062226; ORF062227; ORF062229; ORF062232; ORF062233; ORF062236	2	47.14	1.50	2.40	2	None	ORF014920
SSDNIDEGARQIKASSIVPF	ORF062221; ORF062223; ORF062226; ORF062227; ORF062229; ORF062232; ORF062233; ORF062236	1	42.21	-2.10	2.10	1	None	ORF014920
SSDSVDNAAIK	ORF062221; ORF062223; ORF062226; ORF062227; ORF062229; ORF062232; ORF062233; ORF062236	1	33.95	2.40	2.40	1	None	ORF014920
SSFHGSSGGSSSGGSGSFHGSSGGGTG	ORF072898; ORF072899; ORF072900; ORF072906; ORF072907	1	47.26	1.70	1.70	1	None	ORF017928; ORF072897; ORF072901; ORF072902; ORF072903; ORF072904; ORF072905
SSGPSSDNIDEGARQIK	ORF062221; ORF062223; ORF062226; ORF062227; ORF062229; ORF062232; ORF062233; ORF062236	12	47.45	1.45	4.80	6	None	ORF014920
SSGPSSDNIDEGARQIKASSIVPF	ORF062221; ORF062223; ORF062226; ORF062227; ORF062229; ORF062232; ORF062233; ORF062236	1	27.37	-0.60	0.60	1	None	ORF014920
SSGVIPFAIGVRNADTTELQ	ORF062221; ORF062223; ORF062226; ORF062227; ORF062229; ORF062232; ORF062233; ORF062236	4	47.14	-4.65	5.20	3	None	ORF014920
SSIPKEEVKTEK	ORF083445; ORF083446	6	47.03	4.25	10.60	5	None	ORF001845
SSITSVADGLYDK	ORF090968	1	44.54	-0.60	0.60	1	None	ORF021892
SSIVPFLIGKNVDSEEANKLT	ORF062221; ORF062223; ORF062226; ORF062227; ORF062229; ORF062232; ORF062233; ORF062236	5	46.48	-3.00	4.10	3	None	ORF014920
SSPLSKELR	ORF040982	1	29.05	5.80	5.80	1	None	ORF008943; ORF022249
SSPPTAPSGLSV	ORF101777; ORF101779; ORF101782; ORF101784	1	25.32	-14.30	14.30	1	None	ORF025356
SSQAQLQEIEQSLTK	ORF059178	2	30.22	-1.10	4.40	2	None	ORF013234; ORF059172; ORF059175
SSVDEPREAAPETIAETTLEISTVK	ORF100075; ORF100078; ORF100079	1	47.22	-1.10	1.10	1	None	ORF025240
SSVGGKAAAATVG	ORF083445; ORF083446	1	32.42	2.50	2.50	1	None	ORF001845
SSYEKITINK	ORF091776; ORF091777	1	46.97	5.30	5.30	1	None	ORF021747; ORF022702
STAPASKPIAAPK	ORF053999	3	45.97	5.30	8.50	3	None	ORF012810; ORF054010; ORF054012
STAPTAPSQSQSVK	ORF083445; ORF083446	1	42.94	-7.30	7.30	1	None	ORF001845

Peptide sequence	Incomplete ORF(s)	PSMs (n)	Best −10LgP	Median ppm	Max ∣ppm∣	Replicates (n)	PTM	Complete ORF match(es)
STASILATQESEITVGPFK	ORF053166	2	43.57	-2.70	4.90	2	None	ORF003049; ORF053159; ORF053173; ORF053180
STKDEVQSAVR	ORF062221; ORF062223; ORF062226; ORF062227; ORF062229; ORF062232; ORF062233; ORF062236	3	32.94	0.90	4.40	3	None	ORF014920
STNAVSKPTEEIE	ORF059178	1	37.62	-12.50	12.50	1	None	ORF013234; ORF059172; ORF059175
STRPIIDLYEK	ORF069950	1	29.65	9.70	9.70	1	None	ORF016799; ORF069954; ORF069958; ORF069960
STTEEVDASMNARPH	ORF031536; ORF031542	5	47.46	-7.50	9.40	5	None	ORF004221
STTEEVDASMNARPHR	ORF031536; ORF031542	1	44.90	5.00	5.00	1	None	ORF004221
STTEEVDASMNARPHRVD	ORF031536; ORF031542	3	45.95	-3.70	6.80	3	None	ORF004221
STTEEVDASMNARPHRVDGR MVEPK	ORF031536; ORF031542	5	46.68	3.80	6.90	5	None	ORF004221
SVDDVRGAA	ORF062221; ORF062223; ORF062226; ORF062227; ORF062229; ORF062232; ORF062233; ORF062236	1	27.66	5.70	5.70	1	None	ORF014920
SVDQVFVNVQK	ORF069950	3	41.21	3.90	10.60	3	None	ORF016799; ORF069954; ORF069958; ORF069960
SVDQVFVNVQKIFDREG	ORF069950	4	42.33	-0.65	4.50	4	None	ORF016799; ORF069954; ORF069958; ORF069960
SVKEVFQGIEEHASGL	ORF103626	2	37.66	-4.20	4.20	1	None	ORF025742; ORF103624; ORF103625
SVKNDVKPADTKKPNLT	ORF053999	1	45.62	1.00	1.00	1	None	ORF012810; ORF054010; ORF054012
SVSDGQSAALALASKGVK	ORF062221; ORF062223; ORF062226; ORF062227; ORF062229; ORF062232; ORF062233; ORF062236	1	44.98	-2.60	2.60	1	None	ORF014920
SVSLQEITENETLTPELK	ORF061770	1	36.28	-0.40	0.40	1	None	ORF015690; ORF061765; ORF061767
SVTTLDQDKIK	ORF062221; ORF062223; ORF062226; ORF062227; ORF062229; ORF062232; ORF062233; ORF062236	5	46.83	6.10	11.20	5	None	ORF014920
SVTTLDQDKIKV	ORF062221; ORF062223; ORF062226; ORF062227; ORF062229; ORF062232; ORF062233; ORF062236	1	47.06	5.60	5.60	1	None	ORF014920
SYDSNTLPSDVPK	ORF061770	1	46.40	10.90	10.90	1	None	ORF015690; ORF061765; ORF061767
SYEKITINK	ORF091776; ORF091777	2	44.86	2.45	2.70	2	None	ORF021747; ORF022702
TAAKEAISPTLE	ORF100055; ORF100073; ORF100075; ORF100076; ORF100077; ORF100078; ORF100079	4	43.45	-0.95	5.00	4	None	ORF025240; ORF100065
TAALIQTK	ORF044453	4	35.79	1.75	4.40	4	None	ORF008679; ORF008680
TAATSSIPKEEVKTE	ORF083445; ORF083446	1	45.53	-10.50	10.50	1	None	ORF001845
TAATSSIPKEEVKTEK	ORF083445; ORF083446	4	47.32	1.95	2.30	4	None	ORF001845
TAPASKPIAAPK	ORF053999	2	47.36	5.65	6.50	2	None	ORF012810; ORF054010; ORF054012
TAPTAPSQSQSVK	ORF083445; ORF083446	1	46.38	1.60	1.60	1	None	ORF001845
TAPTAPSQSQSVKKEAQKAS	ORF083445; ORF083446	3	46.37	2.00	2.00	2	None	ORF001845
TASAPSAAASAGSSKVPTSQ	ORF083445	1	45.66	-12.50	12.50	1	None	ORF001845

Peptide sequence	Incomplete ORF(s)	PSMs (n)	Best −10LgP	Median ppm	Max |ppm|	Replicates (n)	PTM	Complete ORF match(es)
TAYLGPKDIVAYEETKA	ORF101777; ORF101779; ORF101782; ORF101784	1	42.47	1.30	1.30	1	None	ORF025356
TDADLIQLLQNYGIK	ORF076580; ORF076584	3	32.46	5.30	10.70	3	None	ORF018429; ORF076577; ORF076579; ORF076582
TDAIALNPNNAIYYGNR	ORF072258; ORF072259	1	34.75	-5.30	5.30	1	None	ORF018021
TDDIEILR	ORF062221; ORF062223; ORF062226; ORF062227; ORF062229; ORF062232; ORF062233; ORF062236	10	37.52	0.40	1.60	6	None	ORF014920
TDEPVVASLR	ORF043012	4	42.09	2.15	5.20	4	None	ORF007631; ORF043008
TDPVSNIK	ORF079438; ORF079444	3	34.14	1.40	2.50	3	None	ORF020265; ORF079440
TDVETIVTVQ	ORF044453	4	26.89	-1.90	4.10	3	None	ORF008677; ORF008679
TDVETIVTVQGK	ORF044453	2	46.50	-4.55	14.70	2	None	ORF008679
TDVETIVTVQGKSISQLVE	ORF044453	2	43.83	-1.85	9.50	2	None	ORF008679
TDWALYTYEGNSNDIR	ORF036644; ORF036647	4	46.04	1.10	10.10	4	None	ORF008344; ORF036655; ORF036666; ORF036670; ORF036673
TEAVVSREEQKSEPAPK	ORF051130; ORF051137	5	47.46	1.70	7.30	4	None	ORF012252
TEDEVERVVT	ORF071696	2	25.11	6.90	8.60	2	None	ORF018184
TEEVDASM(+15.99)NARPH	ORF031536; ORF031542	4	47.05	-0.85	3.60	3	Oxidation (M)	ORF004221
TEEVDASMNARPH	ORF031536; ORF031542	4	47.05	-0.85	3.60	3	Oxidation (M)	ORF004221
TEEVDASMNARPHR	ORF031536; ORF031542	4	32.96	3.60	9.70	4	None	ORF004221
TEEVDASMNARPHRVDGRM(+15.99)VEPK	ORF031536; ORF031542	4	42.70	4.85	7.20	4	Oxidation (M)	ORF004221
TEEVDASMNARPHRVDGRMVEPK	ORF031536; ORF031542	4	42.70	4.85	7.20	4	Oxidation (M)	ORF004221
TEIETIVTDPR	ORF062221; ORF062223; ORF062226; ORF062227; ORF062229; ORF062232; ORF062233; ORF062236	11	46.92	3.00	3.60	6	None	ORF014920
TELHGQQISVEK	ORF038388; ORF038393	1	46.81	-6.60	6.60	1	None	ORF007177; ORF038385; ORF038389
TEPVIALYEGR	ORF076196	1	27.78	-4.90	4.90	1	None	ORF018471; ORF076197; ORF076198
TESAMKKIEDNNT	ORF067090	2	47.14	9.70	10.40	2	None	ORF006342; ORF016564
TESAMKKIEDNNTLV	ORF067090	1	31.37	-5.30	5.30	1	None	ORF006342; ORF016564
TESAMKKIEDNNTLVFIVDVK	ORF067090	7	47.15	-0.10	4.60	2	None	ORF006342; ORF016564
TESSVDEPREAAPETIAETTLEISTVK	ORF100075; ORF100078; ORF100079	3	47.26	-2.40	4.30	3	None	ORF025240
TGDFNWVPGTIPGGR	ORF029551; ORF029560; ORF029561; ORF029562	1	27.79	2.70	2.70	1	None	ORF005045; ORF029552; ORF029553; ORF029563; ORF029564; ORF029566
TGELGDQDEPLTQNDIR	ORF078612	5	47.26	-3.40	3.50	3	None	ORF019339; ORF078617; ORF078626; ORF078629; ORF078634
TGELGDQDEPLTQNDIRNTKDVDM(+15.99)K	ORF078612	5	47.37	-0.30	11.50	4	Oxidation (M)	ORF019339; ORF078617; ORF078626; ORF078629; ORF078634
TGELGDQDEPLTQNDIRNTKDVDM(+15.99)KIGV	ORF078612	23	47.46	1.20	4.50	6	Oxidation (M)	ORF019339; ORF078617; ORF078626; ORF078629; ORF078634
TGELGDQDEPLTQNDIRNTKDVDMK	ORF078612	5	47.37	-0.30	11.50	4	Oxidation (M)	ORF019339; ORF078617; ORF078626; ORF078629; ORF078634

Peptide sequence	Incomplete ORF(s)	PSMs (n)	Best −10LgP	Median ppm	Max ∣ppm∣	Replicates (n)	PTM	Complete ORF match(es)
TGELGDQDEPLTQNDIRNTKDVDMKIGV	ORF078612	23	47.46	1.20	4.50	6	Oxidation (M)	ORF019339; ORF078617; ORF078626; ORF078629; ORF078634
TGGKSVDDVRGAA	ORF062221; ORF062223; ORF062226; ORF062227; ORF062229; ORF062232; ORF062233; ORF062236	8	47.40	-1.05	8.20	6	None	ORF014920
TGPAESSYESR	ORF076580; ORF076584	2	40.03	4.80	8.90	2	None	ORF018429; ORF076577; ORF076579; ORF076582
TGSDGLVNIR	ORF062221; ORF062223; ORF062226; ORF062227; ORF062229; ORF062232; ORF062233; ORF062236	3	43.52	-3.80	10.40	3	None	ORF014920
TGSDGLVNIRDFIIK	ORF062221; ORF062223; ORF062226; ORF062227; ORF062229; ORF062232; ORF062233; ORF062236	3	45.99	-2.70	2.70	2	None	ORF014920
TGSPGGSPGGPSPAQNSTK	ORF081161	2	35.27	-5.50	5.60	2	None	ORF019667
TGVLSDQLYHWTGSD	ORF090968	6	47.46	1.50	3.40	3	None	ORF021892
TGVPDKVKDIYDQAAEK	ORF090968	4	46.61	4.20	4.40	3	None	ORF021892
TGVPQVAFIITGAKSVSDGQSAA	ORF062221; ORF062223; ORF062226; ORF062227; ORF062229; ORF062232; ORF062233; ORF062236	1	44.36	2.20	2.20	1	None	ORF014920
THETAYLGPKDIVA	ORF101777; ORF101779; ORF101782; ORF101784	1	27.14	-3.20	3.20	1	None	ORF025356
THETAYLGPKDIVAYE	ORF101777; ORF101779; ORF101782; ORF101784	1	27.81	0.00	0.00	1	None	ORF025356
TIENVKAKI	ORF082494; ORF082495; ORF082496; ORF082497; ORF082498	2	45.19	4.40	4.40	1	None	ORF001027; ORF001195; ORF005506; ORF011164; ORF107844; ORF107845; ORF107846; ORF107847; ORF107848; ORF107849; ORF107850; ORF107851; ORF107852; ORF107858; ORF107859
TIKEFKELPSIE	ORF062221; ORF062223; ORF062226; ORF062227; ORF062229; ORF062232; ORF062233; ORF062236	6	47.11	1.15	11.60	5	None	ORF014920
TIKEFKELPSIESI	ORF062221; ORF062223; ORF062226; ORF062227; ORF062229; ORF062232; ORF062233; ORF062236	2	44.10	-1.50	2.00	2	None	ORF014920
TIKEFKELPSIESIL	ORF062221; ORF062223; ORF062226; ORF062227; ORF062229; ORF062232; ORF062233; ORF062236	2	45.63	1.85	4.20	1	None	ORF014920
TIKEFKELPSIESILFK	ORF062221; ORF062223; ORF062226; ORF062227; ORF062229; ORF062232; ORF062233; ORF062236	1	39.09	-9.30	9.30	1	None	ORF014920
TIKLVPGQGGAIGGAASGGSFG	ORF072898; ORF072899; ORF072900; ORF072906; ORF072907	2	44.77	-7.75	14.10	2	None	ORF017928; ORF072897; ORF072901; ORF072902; ORF072903; ORF072904; ORF072905
TIKWFINEEERPL	ORF065205; ORF065206	1	32.30	4.70	4.70	1	None	ORF000973; ORF065202
TIKWFINEEERPLT	ORF065205; ORF065206	1	44.90	-3.40	3.40	1	None	ORF000973; ORF065202
TISGLAGSGQAGGAGK	ORF089382	5	47.44	1.50	14.20	4	None	ORF021680; ORF089369; ORF089372; ORF089373; ORF089377; ORF089379; ORF089383
TISGLAGSGQAGGAGKFIK	ORF089382	11	47.46	3.80	10.80	6	None	ORF021680; ORF089369; ORF089372; ORF089373; ORF089377; ORF089379; ORF089383

Peptide sequence	Incomplete ORF(s)	PSMs (n)	Best −10LgP	Median ppm	Max ∣ppm∣	Replicates (n)	PTM	Complete ORF match(es)
TITTVQTAAKEAISPTLE	ORF100055; ORF100073; ORF100075; ORF100076; ORF100077; ORF100078; ORF100079	3	46.44	-5.20	7.70	3	None	ORF025240
TIVERAAQ	ORF066964	1	25.15	-4.10	4.10	1	None	ORF016315
TIVERAAQLA	ORF066964	2	38.47	-0.90	2.40	2	None	ORF016315
TIVERAAQLAIK	ORF066964	3	33.53	-0.80	4.30	3	None	ORF016315
TKVKEEVKEEEEIEAPDRFQ	ORF066694	1	41.63	-3.00	3.00	1	None	ORF016200; ORF066716
TLIRPDGEK	ORF067090	2	42.86	2.25	3.80	2	None	ORF006342; ORF016564
TLIRPDGEKKA	ORF067090	2	45.35	2.10	2.30	2	None	ORF006342; ORF016564
TLIRPDGEKKAYVR	ORF067090	4	46.56	1.40	4.90	2	None	ORF006342; ORF016564
TLSSPTGQSAPLSVQK	ORF061770	2	46.44	1.20	2.10	2	None	ORF015690; ORF061765; ORF061767
TLTTVQGIADDYDK	ORF095859	5	47.26	-0.40	3.40	4	None	ORF013742; ORF023722; ORF058081; ORF058082
TLTTVQGIADDYDKK	ORF095859	6	47.08	2.80	3.40	3	None	ORF013742; ORF023722; ORF058081; ORF058082
TLVITDKEKAE	ORF098458	1	39.20	10.90	10.90	1	None	ORF023230
TLVITDKEKAEK	ORF098458	14	47.36	0.55	4.90	6	None	ORF023230
TLVITDKEKAEKL	ORF098458	14	46.43	-0.30	10.90	6	None	ORF023230
TLVITDKEKAEKLK	ORF098458	15	47.45	0.60	7.30	6	None	ORF023230
TNIPREAVDYIVYGTVIQEVK	ORF045937; ORF045938; ORF045939; ORF045940; ORF045941; ORF045942; ORF045943	5	45.13	-2.60	5.20	4	None	ORF011267; ORF011880
TNKLDLLN	ORF062221; ORF062223; ORF062226; ORF062227; ORF062229; ORF062232; ORF062233; ORF062236	1	26.45	-0.50	0.50	1	None	ORF014920
TNKLDLLNNVR	ORF062221; ORF062223; ORF062226; ORF062227; ORF062229; ORF062232; ORF062233; ORF062236	6	44.17	-1.90	5.20	3	None	ORF014920
TNLKSSGVIPFAIGV	ORF062221; ORF062223; ORF062226; ORF062227; ORF062229; ORF062232; ORF062233; ORF062236	1	32.76	0.10	0.10	1	None	ORF014920
TNQGGNFVVTAAGK	ORF044453	2	46.47	-0.70	4.90	2	None	ORF008679
TPAVKPKDSEVKKAEPEAVISASSSSSVQ	ORF083443	1	27.87	0.40	0.40	1	None	ORF001845
TPLTSMVVTKPE	ORF068100	1	40.65	1.20	1.20	1	None	ORF016152; ORF017153
TPTPVGTTPRPAA	ORF053999	2	46.03	-6.35	14.50	2	None	ORF012810; ORF054010; ORF054012
TRKEDPDENKPVVDLVK	ORF083443	2	46.53	3.70	6.20	1	None	ORF001845
TRPAGSRVEEQVPQ	ORF062221; ORF062223; ORF062226; ORF062227; ORF062229; ORF062232; ORF062233; ORF062236	1	25.24	2.20	2.20	1	None	ORF014920
TRVSELKTDDIEIL	ORF062221; ORF062223; ORF062226; ORF062227; ORF062229; ORF062232; ORF062233; ORF062236	3	39.34	-1.90	2.80	2	None	ORF014920

Peptide sequence	Incomplete ORF(s)	PSMs (n)	Best −10LgP	Median ppm	Max ∣ppm∣	Replicates (n)	PTM	Complete ORF match(es)
TRVSELKTDDIEILR	ORF062221; ORF062223; ORF062226; ORF062227; ORF062229; ORF062232; ORF062233; ORF062236	2	47.02	-0.55	5.00	2	None	ORF014920
TSGADRTEIETIVTD	ORF062221; ORF062223; ORF062226; ORF062227; ORF062229; ORF062232; ORF062233; ORF062236	2	39.46	-0.05	3.90	2	None	ORF014920
TSGADRTEIETIVTDPR	ORF062221; ORF062223; ORF062226; ORF062227; ORF062229; ORF062232; ORF062233; ORF062236	18	47.39	-0.60	3.70	6	None	ORF014920
TSGADRTEIETIVTDPRY	ORF062221; ORF062223; ORF062226; ORF062227; ORF062229; ORF062232; ORF062233; ORF062236	3	45.48	1.90	10.70	3	None	ORF014920
TSGADRTEIETIVTDPRYL	ORF062221; ORF062223; ORF062226; ORF062227; ORF062229; ORF062232; ORF062233; ORF062236	7	45.02	-1.10	3.70	4	None	ORF014920
TSGADRTEIETIVTDPRYLFTIK	ORF062221; ORF062223; ORF062226; ORF062227; ORF062229; ORF062232; ORF062233; ORF062236	5	46.69	-0.90	3.00	3	None	ORF014920
TSGADRTEIETIVTDPRYLFTIK E	ORF062221; ORF062223; ORF062226; ORF062227; ORF062229; ORF062232; ORF062233; ORF062236	1	47.07	-8.00	8.00	1	None	ORF014920
TSIVHLFEWR	ORF068995; ORF069000; ORF069006; ORF069010; ORF069012	10	44.67	-1.25	4.80	6	None	ORF016023; ORF016608
TSSGPSSDNIDEGARQIK	ORF062221; ORF062223; ORF062226; ORF062227; ORF062229; ORF062232; ORF062233; ORF062236	1	41.76	8.30	8.30	1	None	ORF014920
TSSIPKEEVKTEK	ORF083445; ORF083446	4	47.02	3.85	7.20	4	None	ORF001845
TSSTSKGDISPTHSEVLLEDAT GSLRPK	ORF053166	2	47.34	2.30	2.30	1	None	ORF003049; ORF053159; ORF053173; ORF053180
TSVTTLDQDKIK	ORF062221; ORF062223; ORF062226; ORF062227; ORF062229; ORF062232; ORF062233; ORF062236	7	47.08	-1.80	2.30	4	None	ORF014920
TSVTTLDQDKIKV	ORF062221; ORF062223; ORF062226; ORF062227; ORF062229; ORF062232; ORF062233; ORF062236	1	25.19	-1.00	1.00	1	None	ORF014920
TTATAGVEGIGM(+15.99)EHR	ORF080397	4	47.42	4.85	7.90	3	Oxidation (M)	ORF019503
TTATAGVEGIGMEHR	ORF080397	4	47.42	4.85	7.90	3	Oxidation (M)	ORF019503
TTATAGVEGIGMEHRLNPAIR	ORF080397	1	43.25	5.30	5.30	1	None	ORF019503
TTPRPAARASPVAPK	ORF053999	3	38.37	3.10	3.20	3	None	ORF012810; ORF054010; ORF054012
TTVQGIADDYDK	ORF095859	2	45.82	9.85	10.20	2	None	ORF013742; ORF023722; ORF058081; ORF058082
TTVQGIADDYDKK	ORF095859	1	47.12	2.10	2.10	1	None	ORF013742; ORF023722; ORF058081; ORF058082
TVDVKNIVPK	ORF044453	1	44.70	-6.80	6.80	1	None	ORF008679
TVGVEPAADGKGVI	ORF091776; ORF091777	1	28.05	-17.40	17.40	1	None	ORF021747; ORF022702

Peptide sequence	Incomplete ORF(s)	PSMs (n)	Best −10LgP	Median ppm	Max ∣ppm∣	Replicates (n)	PTM	Complete ORF match(es)
TVGVEPAADGKGVIV	ORF091776; ORF091777	6	47.22	1.70	8.30	4	None	ORF021747; ORF022702
TVGVEPAADGKGVIVV	ORF091776; ORF091777	13	47.45	1.80	2.80	6	None	ORF021747; ORF022702
TVGVEPAADGKGVIVVL	ORF091776; ORF091777	4	47.28	-3.05	6.30	4	None	ORF021747; ORF022702
TVGVEPAADGKGVIVVLK	ORF091776; ORF091777	33	47.46	-1.40	3.70	6	None	ORF021747; ORF022702
TVGVEPAADGKGVIVVLKK	ORF091776; ORF091777	2	46.82	5.70	5.90	2	None	ORF021747; ORF022702
TVQGIADDYDK	ORF095859	5	46.40	2.00	5.20	5	None	ORF013742; ORF023722; ORF058081; ORF058082
TVQGIADDYDKK	ORF095859	1	47.12	2.40	2.40	1	None	ORF013742; ORF023722; ORF058081; ORF058082
TVQGIADDYDKKKL	ORF095859	1	41.66	-3.60	3.60	1	None	ORF013742; ORF023722; ORF058081; ORF058082
TVQTAAKEAISPTLE	ORF100055; ORF100073; ORF100075; ORF100076; ORF100077; ORF100078; ORF100079	4	45.01	-2.65	4.60	4	None	ORF025240
VAFIITGAKSVSDGQSAALA	ORF062221; ORF062223; ORF062226; ORF062227; ORF062229; ORF062232; ORF062233; ORF062236	2	43.30	-1.00	1.00	1	None	ORF014920
VANKIGII	ORF067090	3	31.84	-2.10	2.10	2	None	ORF006342; ORF016564
VAQLVGAIAGAGILHA	ORF032526	1	26.09	-6.00	6.00	1	None	ORF004880; ORF005253
VAQLVGAIAGAGILHAVAPANIR	ORF032526	1	45.12	-15.40	15.40	1	None	ORF004880; ORF005253
VATSGADRTEIETIVTDPR	ORF062221; ORF062223; ORF062226; ORF062227; ORF062229; ORF062232; ORF062233; ORF062236	13	47.46	1.10	1.80	6	None	ORF014920
VATSGADRTEIETIVTDPRY	ORF062221; ORF062223; ORF062226; ORF062227; ORF062229; ORF062232; ORF062233; ORF062236	3	47.11	-0.70	3.70	2	None	ORF014920
VATSGADRTEIETIVTDPRYL	ORF062221; ORF062223; ORF062226; ORF062227; ORF062229; ORF062232; ORF062233; ORF062236	2	46.60	3.95	4.20	2	None	ORF014920
VATSGADRTEIETIVTDPRYLFT	ORF062221; ORF062223; ORF062226; ORF062227; ORF062229; ORF062232; ORF062233; ORF062236	1	46.27	5.60	5.60	1	None	ORF014920
VATSGADRTEIETIVTDPRYLFTIK	ORF062221; ORF062223; ORF062226; ORF062227; ORF062229; ORF062232; ORF062233; ORF062236	1	32.11	-1.40	1.40	1	None	ORF014920
VAVIQFSEDPKVE	ORF062221; ORF062223; ORF062226; ORF062227; ORF062229; ORF062232; ORF062233; ORF062236	3	44.32	-4.00	5.30	3	None	ORF014920
VAVIQFSEDPKVEF	ORF062221; ORF062223; ORF062226; ORF062227; ORF062229; ORF062232; ORF062233; ORF062236	1	33.62	-7.30	7.30	1	None	ORF014920
VDASNSVDQVFVNVQ	ORF069950	6	46.67	-3.70	6.00	4	None	ORF016799; ORF069954; ORF069958; ORF069960
VDASNSVDQVFVNVQK	ORF069950	14	47.46	-0.30	4.50	6	None	ORF016799; ORF069954; ORF069958; ORF069960
VDEPLIEPSVYIPL	ORF100296; ORF100297; ORF100298; ORF100299; ORF100300	1	40.01	2.70	2.70	1	None	ORF024865; ORF100294; ORF100295

Peptide sequence	Incomplete ORF(s)	PSMs (n)	Best −10LgP	Median ppm	Max |ppm|	Replicates (n)	PTM	Complete ORF match(es)
VDGRMVEPK	ORF031536; ORF031542	6	42.66	5.85	12.60	6	None	ORF004221
VDGSTRTGSDGLVNIR	ORF062221; ORF062223; ORF062226; ORF062227; ORF062229; ORF062232; ORF062233; ORF062236	2	47.11	-2.60	6.10	2	None	ORF014920
VDGSTRTGSDGLVNIRDFIIK	ORF062221; ORF062223; ORF062226; ORF062227; ORF062229; ORF062232; ORF062233; ORF062236	1	27.80	0.90	0.90	1	None	ORF014920
VDSEEANKLT	ORF062221; ORF062223; ORF062226; ORF062227; ORF062229; ORF062232; ORF062233; ORF062236	4	47.04	-11.50	13.10	4	None	ORF014920
VEANVAQPK	ORF027357	2	36.73	0.90	1.40	2	None	ORF001771
VEFM(+15.99)DDTNRSIIR	ORF041048	8	42.38	0.60	3.60	5	Oxidation (M)	ORF003580
VEFM(+15.99)DDTNRSIIRNVK	ORF041048	4	45.24	-1.65	3.90	2	Oxidation (M)	ORF003580
VEFMDDTNRSII	ORF041048	2	43.01	0.55	2.50	2	None	ORF003580
VEFMDDTNRSIIR	ORF041048	8	42.38	0.60	3.60	5	Oxidation (M)	ORF003580
VEFMDDTNRSIIRNVK	ORF041048	4	45.24	-1.65	3.90	2	Oxidation (M)	ORF003580
VEGLDVDKDKIR	ORF062221; ORF062223; ORF062226; ORF062227; ORF062229; ORF062232; ORF062233; ORF062236	5	47.05	-2.40	3.00	5	None	ORF014920
VEKTITTVQ	ORF100055; ORF100073; ORF100075; ORF100076; ORF100077; ORF100078; ORF100079	1	27.56	4.60	4.60	1	None	ORF025240
VEPAADGKGVIV	ORF091776; ORF091777	2	47.05	-0.90	3.10	2	None	ORF021747; ORF022702
VEPAADGKGVIVV	ORF091776; ORF091777	8	46.87	0.50	3.00	6	None	ORF021747; ORF022702
VEPAADGKGVIVVL	ORF091776; ORF091777	1	39.13	0.70	0.70	1	None	ORF021747; ORF022702
VEPAADGKGVIVVLK	ORF091776; ORF091777	10	47.44	0.95	5.50	6	None	ORF021747; ORF022702
VEPSQGSVGAVTKPVS	ORF051130; ORF051137	1	46.19	0.10	0.10	1	None	ORF012252
VEREQEGTSSTPPPPLVPAGKLS	ORF086801	2	37.03	4.40	4.60	2	None	ORF020966; ORF086789; ORF086800
VEVGLAKEDQLK	ORF095859	1	42.90	8.10	8.10	1	None	ORF023722
VEVQEIVTEEVDVDKESPTEGMKVK	ORF100055; ORF100073; ORF100075; ORF100076; ORF100077	3	47.11	6.30	6.30	1	None	ORF025240; ORF100065
VGLVQFSNDPASLFYLK	ORF062221; ORF062223; ORF062226; ORF062227; ORF062229; ORF062232; ORF062233; ORF062236	1	27.85	-3.10	3.10	1	None	ORF014920
VGNLGNNGNKTELE	ORF027075; ORF027076; ORF027079	1	46.78	6.30	6.30	1	None	ORF004248
VGNLGNNGNKTELER	ORF027075; ORF027076; ORF027079	4	47.38	-0.05	3.50	4	None	ORF004248
VGVEPAADGKGVIV	ORF091776; ORF091777	5	47.25	2.10	4.00	3	None	ORF021747; ORF022702
VGVEPAADGKGVIVV	ORF091776; ORF091777	8	47.26	0.00	3.30	6	None	ORF021747; ORF022702
VGVEPAADGKGVIVVLK	ORF091776; ORF091777	8	47.46	-0.35	3.70	5	None	ORF021747; ORF022702
VGVEPAADGKGVIVVLKK	ORF091776; ORF091777	1	25.10	4.10	4.10	1	None	ORF021747; ORF022702

Peptide sequence	Incomplete ORF(s)	PSMs (n)	Best −10LgP	Median ppm	Max |ppm|	Replicates (n)	PTM	Complete ORF match(es)
VIHVTNLPDSGYTDQDILK	ORF085019	1	46.49	-6.10	6.10	1	None	ORF001870; ORF085013; ORF085017
VIQFSEDPKVE	ORF062221; ORF062223; ORF062226; ORF062227; ORF062229; ORF062232; ORF062233; ORF062236	1	46.45	-1.40	1.40	1	None	ORF014920
VIQFSEDPKVEF	ORF062221; ORF062223; ORF062226; ORF062227; ORF062229; ORF062232; ORF062233; ORF062236	1	29.60	0.60	0.60	1	None	ORF014920
VIQFSEDPKVEFL	ORF062221; ORF062223; ORF062226; ORF062227; ORF062229; ORF062232; ORF062233; ORF062236	1	28.97	1.90	1.90	1	None	ORF014920
VITDKEKAEKLK	ORF098458	1	30.11	-4.70	4.70	1	None	ORF023230
VIYNENNGIIK	ORF050709	1	29.86	3.10	3.10	1	None	ORF009765; ORF010196; ORF037399; ORF037400
VKATETPAANSDQTVAVQE	ORF057182	2	47.39	-3.25	10.40	2	None	ORF012631; ORF057183
VKGPVREGDVLTLLESEREA	ORF041048	2	42.39	-4.75	5.30	2	None	ORF003580
VKQTGVPDKVKDIYDQAAEK	ORF090968	6	47.40	3.40	4.50	2	None	ORF021892
VLETADDIQERR	ORF076172; ORF076177	4	37.29	1.05	2.50	4	None	ORF018463
VLNTNIDGRRKIA	ORF071696	1	37.28	4.90	4.90	1	None	ORF018184
VPNAPDETNVVR	ORF087621; ORF087623	5	45.00	0.50	6.60	5	None	ORF021436
VPPPPPIA	ORF069609	3	27.38	1.30	4.40	2	None	ORF001221; ORF069605; ORF069606; ORF069607; ORF069608
VPPPPPIARAVVPS	ORF069609	3	42.69	-3.80	4.30	3	None	ORF001221; ORF069605; ORF069606; ORF069607; ORF069608
VQRVEPSQGSVGAVTKPVS	ORF051130; ORF051137	1	47.46	3.30	3.30	1	None	ORF012252
VREGDVLTLLESER	ORF041048	20	46.76	-2.35	5.70	6	None	ORF003580
VREGDVLTLLESEREA	ORF041048	31	47.41	-1.20	5.00	6	None	ORF003580
VREGDVLTLLESEREAR	ORF041048	15	47.45	-1.20	6.90	6	None	ORF003580
VREGDVLTLLESEREARR	ORF041048	10	47.14	-0.95	1.50	6	None	ORF003580
VREGDVLTLLESEREARRL	ORF041048	6	45.80	-0.55	2.50	4	None	ORF003580
VRPTEEVMELEKERIELIK	ORF053166	5	47.04	1.60	2.10	5	None	ORF003049; ORF053159; ORF053173; ORF053180
VRPTEEVMELEKERIELIKSQ	ORF053166	1	27.71	-0.90	0.90	1	None	ORF003049; ORF053159; ORF053173; ORF053180
VRVEFMDDTNRSII	ORF041048	1	44.73	-3.70	3.70	1	None	ORF003580
VRVEFMDDTNRSIIRNVK	ORF041048	1	45.71	5.40	5.40	1	None	ORF003580
VSELKTDDIEILR	ORF062221; ORF062223; ORF062226; ORF062227; ORF062229; ORF062232; ORF062233; ORF062236	12	46.97	-4.10	10.80	5	None	ORF014920
VSEPIVESVVVEGQE	ORF100055; ORF100073	1	34.96	0.50	0.50	1	None	ORF025240; ORF100065
VSEPTTEAKVSQPSE	ORF057182	1	37.63	-11.40	11.40	1	None	ORF012631; ORF057183
VSIYTGVLSDQLYHWTGSD	ORF090968	3	47.18	-2.20	3.30	3	None	ORF021892
VSKEQTGELGDQDEPLTQNDIRNTKDVDMKIGV	ORF078612	1	29.69	1.90	1.90	1	None	ORF019339; ORF078617; ORF078626; ORF078629; ORF078634
VSKPSQISKPTPEAPADVDNAADLLSQ	ORF055592	2	32.31	-2.00	3.70	2	None	ORF013351

Peptide sequence	Incomplete ORF(s)	PSMs (n)	Best −10LgP	Median ppm	Max ∣ppm∣	Replicates (n)	PTM	Complete ORF match(es)
VSLQEITENETLTPELK	ORF061770	2	44.67	-4.25	7.50	2	None	ORF015690; ORF061765; ORF061767
VTINARADEDVEPDVIVDK	ORF036644; ORF036647	2	46.93	1.85	2.40	2	None	ORF008344; ORF036655; ORF036666; ORF036670; ORF036673
VTINARADEDVEPDVIVDKVA	ORF036644; ORF036647	1	42.38	-11.60	11.60	1	None	ORF008344; ORF036655; ORF036666; ORF036670; ORF036673
VTREEGSTIPYSGTLLEQAEVTK	ORF103626; ORF103627; ORF103630	1	44.13	-10.80	10.80	1	None	ORF020600; ORF020628; ORF020724; ORF021390; ORF025739; ORF025742; ORF026119; ORF026136; ORF103624; ORF103625; ORF103631; ORF103655; ORF103657; ORF103659
VTTESSVDEPREAAPETIAETTLEISTVK	ORF100075; ORF100078; ORF100079	1	25.63	-2.30	2.30	1	None	ORF025240
VVDLIAYIATKE	ORF088248; ORF088249	4	43.54	3.00	7.50	4	None	ORF021197
VVEGLDVDK	ORF062221; ORF062223; ORF062226; ORF062227; ORF062229; ORF062232; ORF062233; ORF062236	3	38.69	-6.90	11.30	3	None	ORF014920
VVEGLDVDKDKIR	ORF062221; ORF062223; ORF062226; ORF062227; ORF062229; ORF062232; ORF062233; ORF062236	8	47.26	1.55	3.30	6	None	ORF014920
VVEGLDVDKDKIRIG	ORF062221; ORF062223; ORF062226; ORF062227; ORF062229; ORF062232; ORF062233; ORF062236	1	43.40	3.70	3.70	1	None	ORF014920
VVIDVEDTKPL	ORF087579	1	33.17	-7.20	7.20	1	None	ORF021453
WEIENNPTVK	ORF101777; ORF101779; ORF101782; ORF101784	6	37.93	0.40	3.30	6	None	ORF025356
WLPAGTEDSIHIR	ORF095859	3	41.36	-3.50	6.30	2	None	ORF023722
WPARIDEILEPAA	ORF101777; ORF101779; ORF101782; ORF101784	1	26.34	-2.60	2.60	1	None	ORF025356
YDDNQVHTLK	ORF076584	1	46.63	-2.00	2.00	1	None	ORF018429; ORF076582
YEKITINK	ORF091776; ORF091777	3	45.57	2.10	3.10	3	None	ORF021747; ORF022702
YEKITINKN	ORF091776; ORF091777	2	46.18	7.10	8.20	2	None	ORF021747; ORF022702
YGEVIQLQGDQR	ORF095859	1	45.99	-3.20	3.20	1	None	ORF013742; ORF023722; ORF058081
YGFGGGDGFDDRLR	ORF086823	1	32.13	-1.50	1.50	1	None	ORF020980; ORF086821
YGGGQSYGGGQGGYGGSSGGYDG	ORF031536; ORF031542	1	47.46	-7.70	7.70	1	None	ORF004221
YKEETIEKM(+15.99)QE	ORF040982	7	47.38	2.80	8.60	4	Oxidation (M)	ORF008943
YKEETIEKMQE	ORF040982	7	47.38	2.80	8.60	4	Oxidation (M)	ORF008943
YKESLNPLDLEILKEK	ORF086999	2	45.64	3.25	7.00	2	None	ORF021062
YLASVKEVFQGIEEHASGL	ORF103626	1	30.34	-0.60	0.60	1	None	ORF025742; ORF103624; ORF103625
YLYTLVITDKEKAE	ORF098458	1	47.25	-8.40	8.40	1	None	ORF023230
YLYTLVITDKEKAEK	ORF098458	8	47.28	2.40	7.30	4	None	ORF023230
YLYTLVITDKEKAEKL	ORF098458	4	47.04	-1.45	1.60	3	None	ORF023230
YLYTLVITDKEKAEKLK	ORF098458	5	45.71	4.00	4.50	4	None	ORF023230
YPGAPAPGQPTDPK	ORF100266; ORF100267; ORF100268	2	44.05	9.30	13.30	2	None	ORF024560
YPGAPAPGQPTDPKV	ORF100266; ORF100267; ORF100268	2	29.16	-14.50	18.90	2	None	ORF024560

Peptide sequence	Incomplete ORF(s)	PSMs (n)	Best −10LgP	Median ppm	Max ∣ppm∣	Replicates (n)	PTM	Complete ORF match(es)
YPGAPAPGQPTDPKVA	ORF100266; ORF100267; ORF100268	2	46.28	-3.35	4.40	2	None	ORF024560
YPGAPAPGQPTDPKVAG	ORF100266; ORF100267; ORF100268	8	47.45	2.05	5.30	6	None	ORF024560
YPHWPARIDEILEPAA	ORF101777; ORF101779; ORF101782; ORF101784	14	46.99	-3.10	4.10	6	None	ORF025356
YSTTEEVDASMNARPH	ORF031536; ORF031542	2	43.19	-3.50	6.40	2	None	ORF004221
YSTTEEVDASMNARPHRVDG RMVEPK	ORF031536; ORF031542	3	46.52	0.70	7.40	3	None	ORF004221
YTGVLSDQLYHWTGSD	ORF090968	1	26.85	-0.10	0.10	1	None	ORF021892
YTLVITDKEKAEK	ORF098458	5	47.11	-7.50	17.60	3	None	ORF023230
YTLVITDKEKAEKL	ORF098458	2	46.89	0.60	3.20	2	None	ORF023230
YTLVITDKEKAEKLK	ORF098458	4	46.73	-0.55	7.10	4	None	ORF023230
YVGNLGNNGNKTELER	ORF027075; ORF027076; ORF027079	1	44.68	-6.20	6.20	1	None	ORF004248

Note: Table S4B contains 1,099 unique peptide sequences. PSM count and confidence/mass-error summaries are shown once per unique peptide. All listed peptides also matched complete ORFs. PTM denotes the identified peptide modification; "None" indicates that no modification was assigned.

Table S5. Representative transcript IDs, candidate annotations, sample-level TPM values, group mean TPM values, descriptive expression ratios, and available gene-level differential-expression statistics underlying Figure 6C.

Gene ID	Transcript ID	ORF ID	NR annotation	Immune-effector class	QS_C_1	QS_C_2	QS_C_3	QS_I_1	QS_I_2	QS_I_3	Control mean TPM	Infection mean TPM	Descriptive log_2 ratio	Mean TPM direction	DESeq2 log_2FC (Infection/ Control)	P value	Adjusted P	Gene-level DE statistics available
QSP_Contig1211 _0003	QSP_Contig1211 _0003	ORF021800	PREDICTED: hepcidin [Nanorana parkeri]	Defense-peptide precursor	8.65	0.00	0.00	0.00	0.00	0.00	2.88	0.00	-4.899	Higher in control	-1.627	0.687	1.000	Yes
MSTRG.9309	MSTRG.9309.1	ORF105958	pleskein-1 antimicrobial peptide precursor [Nanorana pleskei]	Defense-peptide precursor	18.58	235.49	21.85	52.51	33.20	27.93	91.97	37.88	-1.278	Higher in control				No
QSP_Contig828_ 0015	QSP_Contig828_ 0015	ORF013948	Atonp2 precursor [Nanorana ventripunctata]	Defense-peptide precursor	43.57	21.71	17.16	19.85	20.01	27.53	27.48	22.46	-0.290	Higher in control	-0.198	0.727	0.999	Yes
QSP_Contig950_ 0023	MSTRG.12214.1	ORF033395	beta-defensin [Quasipaa spinosa]	Defense-peptide precursor	21.68	61.40	43.73	44.00	17.06	80.17	42.27	47.08	0.155	Near-balanced	0.290	0.667	0.999	Yes
A17034	MSTRG.8894.2	ORF104211	PREDICTED: temporin-H-like [Nanorana parkeri]	Defense-peptide precursor	0.48	12.52	1.07	10.65	0.45	5.90	4.69	5.67	0.268	Higher in infection	0.405	0.748	0.999	Yes
MSTRG.8892	MSTRG.8892.1	ORF104207	pleskein-3 [Nanorana pleskei]	Defense-peptide precursor	2.31	10.48	0.52	12.08	0.11	4.54	4.44	5.58	0.323	Higher in infection				No
QSP_Contig300_ 0067	MSTRG.17202.1	ORF050918	PREDICTED: cystatin-F [Nanorana parkeri]	Defense-peptide precursor	0.51	0.99	0.25	1.01	0.89	0.46	0.58	0.79	0.376	Higher in infection	0.505	0.439	0.999	Yes
MSTRG.8891	MSTRG.8891.1	ORF104205	pleskein-1 antimicrobial peptide precursor [Nanorana pleskei]	Defense-peptide precursor	2.98	19.07	1.13	31.12	17.50	16.33	7.73	21.65	1.475	Higher in infection				No
MSTRG.10278	MSTRG.10278.1	ORF027052	PREDICTED: preprofallaxidin-5-like [Nanorana parkeri]	Defense-peptide precursor	40.27	2021.89	282.23	3657.60	1800.37	3806.54	781.46	3088.17	1.982	Higher in infection				No
MSTRG.9310	MSTRG.9310.1	ORF105960	PREDICTED: temporin-H-like [Nanorana parkeri]	Defense-peptide precursor	32.91	145.29	116.10	1242.94	573.10	1591.64	98.10	1135.89	3.532	Higher in infection				No

Gene ID	Transcript ID	ORF ID	NR annotation	Immune-effector class	QS_C_1	QS_C_2	QS_C_3	QS_I_1	QS_I_2	QS_I_3	Control mean TPM	Infection mean TPM	Descriptive log_2 ratio	Mean TPM direction	DESeq2 log_2FC (Infection/ Control)	P value	Adjusted P	Gene-level DE statistics available
QSP_Contig571_0044	MSTRG.1197.1	ORF032579	C-X-C motif chemokine 10-2 [Quasipaa spinosa]	Chemokine	9.08	5.51	7.80	4.15	6.16	2.60	7.46	4.30	-0.780	Higher in control	-0.371	0.418	0.999	Yes
QSP_Contig106_0002	MSTRG.3404.1	ORF085087	PREDICTED: C-C motif chemokine 19-like [Nanorana parkeri]	Chemokine	2.88	2.75	2.92	2.00	1.78	1.72	2.85	1.83	-0.610	Higher in control	-0.462	0.209	0.999	Yes
QSP_Contig33_0001	MSTRG.1198.1	ORF032612	C-X-C motif chemokine 10-3 [Quasipaa spinosa]	Chemokine	3.19	5.21	7.12	6.70	3.09	1.98	5.17	3.92	-0.390	Higher in control	0.226	0.664	0.999	Yes
QSP_Contig312_0001	MSTRG.14523.1	ORF041573	C-X-C motif chemokine 14 [Mantella aurantiaca]	Chemokine	41.22	30.22	25.39	25.28	21.39	28.18	32.28	24.95	-0.370	Higher in control	-0.168	0.532	0.999	Yes
QSP_Contig106_0004	MSTRG.3409.1	ORF085145	C-C motif chemokine 27 [Mantella aurantiaca]	Chemokine	5.40	3.11	19.37	2.05	3.26	17.90	9.29	7.74	-0.261	Higher in control	1.059	0.136	0.999	Yes
QSP_Contig662_0017	QSP_Contig662_0017	ORF011898	PREDICTED: C-C motif chemokine 20-like [Nanorana parkeri]	Chemokine	17.51	57.71	22.17	48.77	69.09	37.35	32.46	51.74	0.671	Higher in infection	0.843	0.097	0.999	Yes
QSP_Contig571_0043	QSP_Contig571_0043	ORF002907	C-X-C motif chemokine 10-like [Aquarana catesbeiana]	Chemokine	3.38	3.82	1.04	5.94	10.31	4.17	2.75	6.81	1.279	Higher in infection	1.482	0.213	0.999	Yes
MSTRG.3402	MSTRG.3402.1	ORF085085	PREDICTED: C-C motif chemokine 21-like [Nanorana parkeri]	Chemokine	0.99	0.42	0.14	1.70	1.27	1.34	0.52	1.44	1.317	Higher in infection				No
QSP_Contig662_0018	QSP_Contig662_0018	ORF011899	PREDICTED: C-C motif chemokine 2-like [Nanorana parkeri]	Chemokine	0.00	0.00	0.00	1.29	0.00	2.03	0.00	1.11	3.593	Higher in infection	0.181	0.676	0.999	Yes
QSP_Contig256_0002	QSP_Contig256_0002	ORF001646	PREDICTED: interferon lambda-3-like [Nanorana parkeri]	Interferon	0.42	0.00	0.12	0.00	0.00	0.00	0.18	0.00	-1.485	Higher in control	-2.257	0.572	1.000	Yes
QSP_Contig256_0006	QSP_Contig256_0006	ORF001650	PREDICTED: interferon lambda-3-like [Nanorana parkeri]	Interferon	0.00	0.00	0.51	0.00	0.00	0.00	0.17	0.00	-1.433	Higher in control	-2.347	0.556	1.000	Yes
QSP_Contig823_0004	QSP_Contig823_0004	ORF003371	PREDICTED: interferon lambda-3-like [Nanorana parkeri]	Interferon	0.00	0.18	0.12	0.00	0.00	0.00	0.10	0.00	-1.000	Higher in control	-1.674	0.678	1.000	Yes
QSP_Contig256_0003	QSP_Contig256_0003	ORF001647	PREDICTED: interferon lambda-3-like [Nanorana parkeri]	Interferon	0.21	0.00	0.00	0.00	0.00	0.00	0.07	0.00	-0.766	Higher in control	-0.761	0.852	1.000	Yes
QSP_Contig256_0004	QSP_Contig256_0004	ORF001648	PREDICTED: interferon lambda-3-like [Nanorana parkeri]	Interferon	0.00	0.09	0.00	0.00	0.10	0.00	0.03	0.03	0.037	Near-balanced	0.201	0.961	1.000	Yes
QSP_Contig823_0002	QSP_Contig823_0002	ORF003369	PREDICTED: interferon lambda-3-like [Nanorana parkeri]	Interferon	0.00	0.09	0.00	0.00	0.10	0.00	0.03	0.03	0.037	Near-balanced	0.201	0.961	1.000	Yes
QSP_Contig240_0035	QSP_Contig240_0035	ORF008195	interferon gamma [Aquarana catesbeiana]	Interferon	0.22	0.57	1.37	0.43	1.43	0.40	0.72	0.75	0.057	Near-balanced	0.178	0.897	0.999	Yes
MSTRG.957	MSTRG.957.1	ORF106524	interleukin-15 isoform X1 [Pyxicephalus adspersus] >XP_072261965.1 interleukin-15 isoform X1 [Pyxicephalus adspersus]	Interleukin/cytokine	3.14	6.54	10.93	0.00	8.92	0.00	6.87	2.97	-1.181	Higher in control				No
QSP_Contig988_0005	MSTRG.25991.1	ORF082298	unnamed protein product [Staurois parvus]	Interleukin/cytokine	1.71	1.60	1.38	1.60	1.44	0.81	1.56	1.28	-0.266	Higher in control	-0.244	0.467	0.999	Yes
QSP_Contig160_0003	MSTRG.11767.2	ORF032009	PREDICTED: interleukin-17D [Nanorana parkeri]	Interleukin/cytokine	5.58	4.75	1.86	6.35	3.20	1.54	4.06	3.70	-0.133	Near-balanced	0.126	0.768	0.999	Yes
QSP_Contig211_0027	MSTRG.13371.1	ORF038004	PREDICTED: pro-interleukin-16 [Nanorana parkeri]	Interleukin/cytokine	1.10	2.38	0.75	1.43	1.24	1.33	1.41	1.33	-0.075	Near-balanced	0.112	0.802	0.999	Yes

Gene ID	Transcript ID	ORF ID	NR annotation	Immune-effector class	QS_C_1	QS_C_2	QS_C_3	QS_I_1	QS_I_2	QS_I_3	Control mean TPM	Infection mean TPM	Descriptive log_2 ratio	Mean TPM direction	DESeq2 log_2FC (Infection/ Control)	P value	Adjusted P	Gene-level DE statistics available
QSP_Contig674_0013	QSP_Contig674_0013	ORF003147	PREDICTED: cytokine-like protein 1 [Nanorana parkeri]	Interleukin/cytokine	0.00	0.00	0.87	0.00	0.00	0.88	0.29	0.29	0.012	Near-balanced	0.176	0.965	1.000	Yes
QSP_Contig48_0001	MSTRG.7020.1	ORF097513	PREDICTED: interleukin-17D-like [Nanorana parkeri]	Interleukin/cytokine	85.84	46.46	36.07	59.34	53.16	93.65	56.12	68.72	0.292	Higher in infection	0.627	0.140	0.999	Yes
QSP_Contig29_0012	MSTRG.1218.1	ORF033281	PREDICTED: interleukin-8-like [Nanorana parkeri]	Interleukin/cytokine	7.58	8.54	8.35	10.24	9.58	14.01	8.16	11.28	0.462	Higher in infection	0.650	0.067	0.999	Yes
A29000	MSTRG.15746.2	ORF046116	interleukin-17A/F1alpha [Quasipaa spinosa]	Interleukin/cytokine	0.35	0.20	4.83	1.57	1.08	19.51	1.79	7.39	1.983	Higher in infection	2.169	1.000	1.000	Yes
QSP_Contig531_0024	QSP_Contig531_0024	ORF005748	interleukin-10 [Aquarana catesbeiana]	Interleukin/cytokine	0.21	0.00	0.00	0.42	0.00	1.97	0.07	0.80	2.399	Higher in infection	3.731	0.133	0.999	Yes
QSP_Contig466_0030	MSTRG.25693.4	ORF080970	PREDICTED: LOW QUALITY PROTEIN: T-cell immunomodulatory protein [Nanorana parkeri]	Other immune-related	1.07	1.34	0.96	1.30	1.00	1.09	1.12	1.13	0.008	Near-balanced	0.146	0.516	0.999	Yes

Note: TPM values are transcript-level values for the representative transcript shown. Control and infection means are calculated from three biological replicates per group. The descriptive ratio is log2[(infection mean TPM + 0.1)/(control mean TPM + 0.1)]; a pseudocount of 0.1 TPM was added to both group means to avoid undefined values when a group mean was zero. "Near-balanced" denotes |descriptive log2 ratio| < 0.25. Gene-level DESeq2 statistics are shown where available and are distinct from the descriptive TPM ratio. Descriptive log_2 ratios were calculated from unrounded group means; displayed TPM means are rounded for presentation.
